# Type catalogue of the class Diplopoda from the Zoological Institute of the Russian Academy of Sciences, St. Petersburg

**DOI:** 10.3897/zookeys.1289.198005

**Published:** 2026-08-10

**Authors:** Dmitri V. Logunov, Sergei I. Golovatch

**Affiliations:** 1 Zoological Institute of the Russian Academy of Sciences, Universitetskaya Embankment 1, St Petersburg, 199034, Russia A.N. Severtsov Institute of Ecology and Evolution, Russian Academy of Sciences Moscow Russia; 2 A.N. Severtsov Institute of Ecology and Evolution, Russian Academy of Sciences, Leninsky prospekt 33, Moscow 119071 Russia Zoological Institute of the Russian Academy of Sciences St Petersburg Russia

**Keywords:** Collection, Myriapoda, museology, nomenclature, type locality, type specimens

## Abstract

A complete catalogue of the type specimens of Diplopoda housed in the Zoological Institute of the Russian Academy of Sciences, St. Petersburg (as of May of 2026) is provided. Species of 29 millipede families in nine orders are represented by types or type series: a total of 157 species (977 specimens), of which 80 are represented by primary types (holotypes or lectotypes). The taxonomic status of ten ancient species described by JF Brandt is clarified based on a re-examination of the types, and the following new combinations are proposed: ?*Allawrencius capensis* (Brandt, 1841), **comb. nov**. ex *Polydesmus* Latreille, 1802/03; *Anadenobolus grandis* (Brandt, 1840), **comb. nov**. ex *Spirobolus* Brandt, 1833; *Anadenobolus marcgravii* (Brandt, 1840), **comb. nov**. ex *Spirobolus* Brandt, 1833; *Anadenobolus olfersii* (Brandt, 1833), **comb. nov**. ex *Spirobolus* Brandt, 1833; *Camaricoproctus laticollis* (Brandt, 1840), **comb. nov**. ex *Julus* Linnaeus, 1758; *Iulomorpha rotundata* (Brandt, 1840), **comb. nov**. ex *Julus* Linnaeus, 1758; *Poratophilus capensis* (Brandt, 1840), **comb. nov**. ex *Julus* Linnaeus, 1758; *Spinotarsus bicuspidatus* (Brandt, 1840), **comb. nov**. ex *Julus* Linnaeus, 1758; *Spinotarsus flavotaeniatus* (Brandt, 1840), **comb. nov**. ex *Julus* Linnaeus, 1758; *Orthoporoides erythropareius* (Brandt, 1840) = *Orthoporoides trigonyger* (Brandt, 1840), **syn. nov. et comb. nov**. ex *Julus* Linnaeus, 1758.

## Introduction

The present paper is a detailed catalogue of the Diplopoda types held at the Zoological Institute of the Russian Academy of Sciences, St. Petersburg, Russia (hereafter ZISP; Table 1), compiled and presented here for the first time. The nomenclature and taxonomic status of ten ancient species described by JF Brandt, primarily from South Africa, have been more or less clarified based on a re-examination of the relevant type material; all these species have also been illustrated for the first time, their new combinations allotted based solely on the expert opinion of one of us (SIG), often thereby inviting revision.

**Figure 1.**
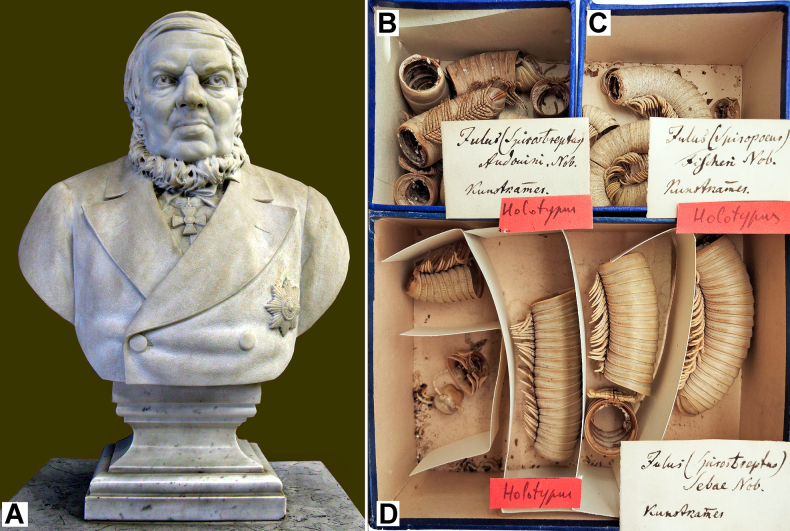
A sculptural portrait of Johann Friedrich Brandt (1802–1879) and Diplopoda specimens from the original collection of Kunstkammer. **A**. Bust of JF Brandt decorating one of the halls of the Zoological Museum, created by LA Bernshtam and donated by SA Kun. Photographed by Vladimir Azaryan; **B**. Holotype ♀ of *Spirostreptus audouini* (Brandt, 1833); **C**. Holotype ♀ of *Spiropoeus fischeri* (Brandt, 1833); **D**. Holotype ♀ of *Spirostreptus sebae* (Brandt, 1833). **B, C, D**. Photographed by DVL.

The accumulation of millipede types in Russia began, following the due credentials of Alexander von Humboldt (1769–1859) and Karl Ernst von Baer (1792–1876), with the invitation of Johann Friedrich Brandt (1802–1879; Fig. 1A) to St. Petersburg in 1830, his election as Ordinary Academician in 1831, and appointment as the Director of the Zoological Museum of the St. Petersburg Academy of Sciences in 1832 (Strauch 1889; Sokolov and Shishkin 2005; Slepkova 2016, 2017). Brandt immediately became russified as Fedor Fedorovich Brandt and, being the first director of the newly established Zoological Museum, he incorporated therein most of the animal items from the so-called Kunstkammer (= Kunstkamera, = Chamber of Curiosities), Russia’s first natural history state collection. That collection was founded in 1714 by Tsar Peter-the-Great. A century later (in the 1820–30s) it was split and transformed into several specialised museums, including the Zoological Museum (1832–1931), which then became ZISP (1931–present day) (Slepkova 2008, 2017; Slepkova and Yusupova 2018). Based on the catalogues of the Zoological Museum from Brandt’s personal archive (Slepkova 2013), the collection of Myriapoda, or ‘thousand-legged’ as they were called at the time, consisted of 218 specimens belonging to 120 species. This number obviously included both Diplopoda and Chilopoda. Most of that material, including types (e.g., Fig. 1B–D), is still available at ZISP.

Since Brandt moved from Berlin to St. Petersburg in 1831, and until 1841, he mainly focused his research on Myriapoda and mammals (see Sokolov and Shishkin 2005), both anatomy and taxonomy, in fact being among the pioneers of myriapodology. As regards the Diplopoda alone, altogether he published 12 papers, becoming the sole author of four orders and families, 15 generic categories, and 81 species (Golovatch and Hoffman 2000). At the end of his myriapodological career, Brandt (1841a) published verbatim a separate monograph combining all of his contributions that previously had appeared in the Bulletin Scientifique published by l‘Académie Impériale des Sciences de Saint-Pétersbourg. Currently, Brandt's diplopod types are about equally shared between the collections of the Berlin and St. Petersburg museums, the ZISP share being updated and considered in detail below.

Originally, all Brandt’s myriapod specimens were pinned and dried. They had been kept untouched in a separate cabinet since the late 1840s up to 1879, when an occasional audit of his collection revealed a significant damage by pests (Strauch 1889). Therefore, most of his material was then moistened and preserved in alcohol. Yet, some of his type specimens are still kept dried and pinned specimens (see below for details). By July 1882, the ZISPMyriapoda collection consisted of 104 jars of largely undetermined spirit-preserved samples and three drawers of dried specimens, which were assembled and primarily identified/described by Brandt (Strauch 1889).

Further advance in the taxonomic study of Diplopoda in the Russian Empire, however modest, was associated with the activities of Victor Ivanovich Motschoulsky (1810–1871) and Vyacheslav Stepanovich Muralewicz (1881–1942?). Each described only a single new millipede species from the Caucasus, but left no type material in ZISP (Golovatch 2014b; Golovatch et al. 2022b). Further two sporadic contributions belonged to Eduard Iwanowitsch von Gerstfeldt (1798–1878) and Erich Haase (1857–1894). Each likewise described a new species of Diplopoda from Siberia (Gerstfeldt 1858; Haase 1880), Gerstfeldt’s type still remaining housed in the ZISP collection (e.g., Mikhaljova 2017). Stuxberg (1876a, 1876b) described as many as five diplopod species from Siberia, but their type material is deposited in the Stockholm Museum, Sweden. Among the Russian contributors of that time, the taxonomic paper of 1897, by Gavriil Efimovich Timotheew (= Timofeyev) (1881–1926), that beautifully described and illustrated two new diplopod species, both still valid, from the southern parts of European Russia is outstanding.

A much more important progress in the study of Diplopoda systematics, mostly from Crimea, the Caucasus, Central Asia and Ukraine, was achieved thanks to the works of Russian myriapodologist Nikolai Georgievich (= Yegorovich) Lignau (1873–1940) (see Bogachik et al. 2005; Anonymous 2026). Lignau actively participated in the development of the ZISP millipede collections (e.g., Lignau 1903, 1907, 1915, 1929, 1933) and also deposited types of some newly described species in the museum (Lignau 1924). Unfortunately, of the approximately 16 new millipede species he altogether described, type specimens of only three are currently in the collection (see below for details).

**Figure 2.**
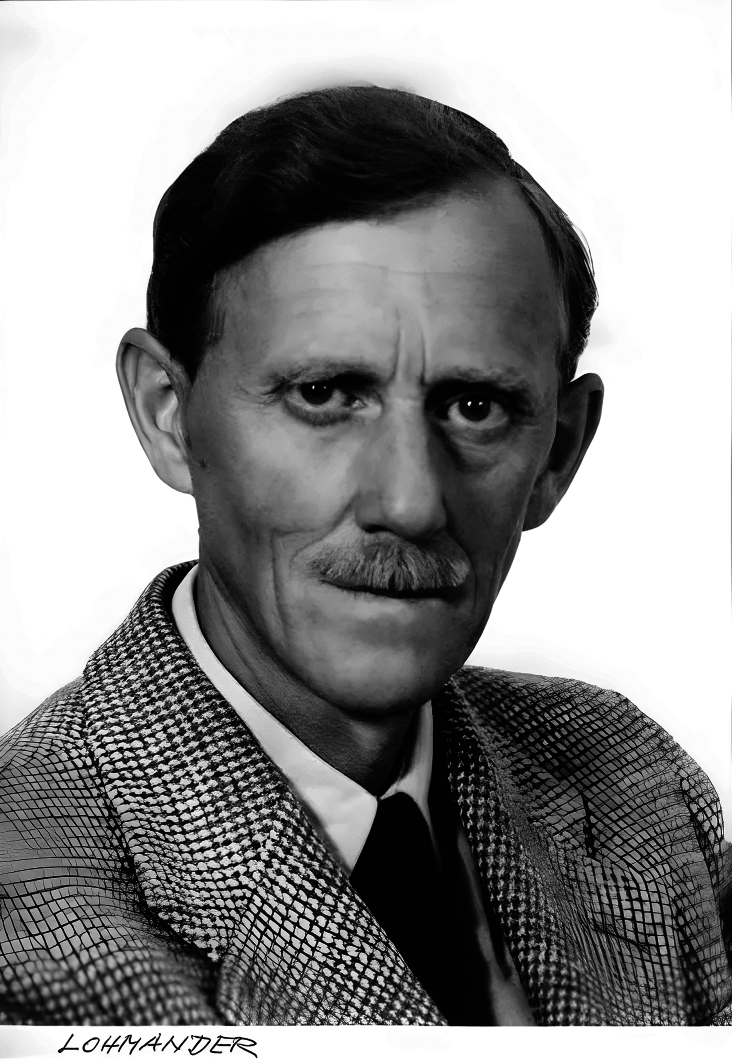
Hans Gustaf Lohmander (1896–1961), a prominent Swedish myriapodologist, the late 1950s. Family archive of Otto Kraus, photographer unknown. Credit: Beate Kraus, the daughter of Otto Kraus.

The next stage in the development of the ZIPS Diplopoda collection was largely linked to the taxonomic research of Hans Gustaf Lohmander (1896–1961; Fig. 2), a prominent Swedish zoologist and taxonomist who specialised in Myriapoda (millipedes and centipedes), as well as land molluscs, woodlice, and false-scorpions; see Lindroth (1961) and Hagström (1992).

Since 1927, Lohmander started publishing, always high-standard for his time, on the diplopod material he received from various parts of the Soviet Union, his descriptions always being very clear and nicely illustrated. The opportunity to study the rich ZISP collections of Diplopoda was provided to Lohmander by Prof. Vladimir Vladimirovich Redikorzev (1873–1942), a versatile zoologist who headed the First Department of Invertebrates at ZISP (1913–1933) and was a recognised expert on false-scorpions and ascidians (Olenev and Popov 1945). Most of the research materials that Lohmander borrowed from the ZISP collection and published in the form of monographs still constitute the basis of what we know about the Diplopoda diversity of Iran, Central Asia, and the Caucasus, respectively (Lohmander 1932a, 1933, 1936). In all these papers, Redikorzev was mentioned as the main provider of the studied material. At present, the majority of Lohmander’s types are held at GMNH and ZISP (details below), but some are also housed at FMNH and the National Museum of Natural History in Kyiv (Ukraine) (e.g., Martynov et al. 2021).

Lohmander apparently mainly made and used temporary slides (at least for the material he borrowed from ZISP) to study the details of morphological structures, including gonopods, after which he placed them back to alcohol in long glass microvials. Unfortunately, since the jars in which such microvials were stored, previously kept at IPEE and now at ZMMU, were only provided with brief coded labels, they are practically useless for further work. Yet, permanent micropreparations made by Lohmander are definitely present in GMNH (O. Gran, pers. comm., 26.I.2026); for further details, see below in the accounts of individual species. Based on results from an audit of the ZISPDiplopoda apparently conducted in the late 1960s, there were 391 identified species in 83 genera (Logunov, unpublished data). Such a significant increase in the number of Diplopoda species in the ZISP collection became possible, to a large extent, due to the research of HG Lohmander with the active assistance and support from VV Redikorzev.

Jan Gulička (1925–2009), a Slovak diplopodologist from the Bratislava University, made two important contributions to the millipede fauna of the former Soviet Union (Gulička 1963, 1972). Altogether, based on the material received on loan from IPEE (courtesy Professor MS Ghilarov), he described 13 new sub/species from five new sub/genera, as well as two new families of Diplopoda, mainly from Siberia. Both these papers explicitly stated that the types were kept at IPEE, but in fact Gulička returned only some of the types, all presently in the ZISP collection, and only after a scandal with the involvement of his higher university officials. Moreover, he returned only torsos of a few types, keeping the dissected gonopods at Bratislava and thus very strongly reducing the scientific value of those types. Fortunately, the identities of all of Gulička’s millipede taxa from the former Soviet Union, many of which were initially quite mysterious because his descriptions were not sufficiently detailed, have since been fully clarified (e.g., Enghoff 1985; Jeekel 1988; Mikhaljova 2017; VandenSpiegel and Golovatch 2023). His main collection of Diplopoda is presently housed in SMNPS, including the samples still embezzled from IPEE.

Regrettably, a similarly sad story concerned the diplopod samples from the Caucasus that Prof. MS Ghilarov shipped on loan for identification to Jaroslav Lang (1910–1972), a Czech myriapodologist from Prague. Not only has nothing from that material ever been returned to Moscow as due, but, from among the abundant samples from the Caucasus, Lang (1959) described only a single new species, albeit misidentified even at the generic and family levels. That material did not concern the ZISP collection and its identity has long been rectified (Antić and Makarov 2016).

The latest period of development of the ZISPDiplopoda collections (especially in the 1980s) is primarily associated with the scholarly work and support of one of us (SIG), who all his life is engaged in millipede systematics, biology, ecology, and geography. Until now SIG has published nearly 500 papers, largely taxonomic and faunistic, and authored or co-authored ~ 500 taxa of Diplopoda. The ZISP collection remained his target to revise the main repository until a curator appeared at ZMMU in 1991. Therefore, most of SIG’s diplopod type and non-type collections have since been deposited in ZMMU. However, now that ZISP has recently (since late 2022) appointed DVL as a curator of arachnids and millipedes, it has become possible to compile the first complete catalogue of Diplopoda types housed there.

The primary and secondary types of Diplopoda in the ZISP collection (as of May 2026) are listed below, arranged alphabetically by orders, families and then species names as originally described. A summary of ZISPDiplopoda types is given in Table 1. As evidenced from this table, a total of 157 species are represented by type specimens, of which 80 species (51%) are represented by primary types (= name-bearing types, sensu ICZN 1999: article 72.1.2): holotypes (71 species; 45%) or lectotypes (8; 5%). Four millipede families are the richest in types: Julidae (38 species; 24%), Polydesmidae (23; 15%), Paradoxosomatidae (17; 11%), and Spirostreptidae (14; 9%), which combined account for more than half of all millipede species represented by types (92 species; 59%). The ZISP collection contains the types of millipede species described mainly by three authors, whose contribution to the formation of this collection is therefore the most significant: JF Brandt (43 species; 27%), H Lohmander (37; 24%), and SIG (50; 32%) describing a total of 130 species (83%). Yet, the types of some contemporary authors, such as Henrik Enghoff (13 types; 8%), appeared in ZISP thanks to the active assistance of SIG.

The aim of the present paper is twofold: (1) to provide, for the first time, a complete catalogue of all Diplopoda types available in the ZISP collection; and (2) to clarify the nomenclature and taxonomic status of ten species described by JF Brandt, primarily from South Africa.

**Table 1.** Number of millipede (Diplopoda) species and specimens represented by types at the Zoological Institute RAS (ZISP), by family; the nomenclature mainly follows Sierwald et al. (2026).

**Order/Family**	**Number of species**								**Number of specimens**
	**Primary types**		**Primary and secondary type(s)**			**Secondary types**		**Total**	
	**HT**	**LT**	**HT+PT**	**ST**	**LT+PLT**	**PT**	**PLT**		
** Callipodida **									
** Callipodidae **	1							1	1
** Caspiopetalidae **	1		1	1				3	8
** Chordeumatida **									
** Entomobielziidae **			1					1	43
** Kashmireumatidae **						1		1	1
** Megalotylidae **						1		1	2
** Metopidiotrichidae **			1			1		2	14
** Glomerida **									
** Glomeridae **	1		3			2		6	15
** Julida **									
** Blaniulidae **	2		4	1		1		8	32
** Julidae **	12		4	20		2		38	215
** Mongoliulidae **			2					2	101
** Nemasomatidae **			3	1	1			5	61
**PLATYDESMIDA**									
** Platydesmidae **			1					1	10
** Polydesmida **									
** Dalodesmidae **	1	1						2	2
** Opisotretidae **						1		1	2
** Paradoxosomatidae **	4		1			12		17	29
** Platyrhacidae **			1					1	2
** Polydesmidae **	7		3	6	1	6		23	373
** Trichopolydesmidae **						4		4	6
** Xystodesmidae **	1					1		2	2
** Polyzoniida **									
** Polyzoniidae **				1				1	1
** Sphaerotheriida **									
** Sphaerotheriidae **	1	3			2		2	8	16
** Zephroniidae **		1						1	1
**Spirolobida**									
** Pachybolidae **					1			1	2
** Rhinocricidae **	1	1		1				3	5
** Spirobolidae **				1				1	2
** Spirostreptida **									
** Iulomorphidae **	1							1	1
** Harpagophoridae **	2	1			2			5	9
** Odontopygidae **	1	1			1			3	4
** Spirostreptidae **	10	1		2			1	14	16
**Total**	**46**	**9**	**25**	**34**	**8**	**32**	**3**	**157**	**977**

Abbreviations: HT – holotype; LT – lectotype; PLT – paralectotype; PT – paratype; St – syntype.

## Materials and methods

The following catalogue provides a complete list of the type specimens in the ZISP collection as of May 2026, arranged alphabetically by orders, families and, within the families, by species names as originally described, in the following format:

Species name, author, year of the description (genus in which the species was originally described).

Literature citation for the original description and indication of which sex(es) was/were described (D♂, D♀, or D♂♀).

**Type/s** Number, gender and type of specimens, accession number/s.

**Type locality** Extract from holotype/lectotype locality label. Approximate coordinates for type localities have been obtained via a Google map search (if it is possible). If the coordinates were given in the original description (e.g., Golovatch 2014a, 2015) or by subsequent researchers, they are reproduced and the corresponding reference is provided. All geographical names relating to type localities are given in their modern interpretation and spelling.

**Label data** Transcription of locality label/s of all type specimens. To ensure an unambiguous recognition of any label, its content is reproduced exactly, including all full stops/commas, typos, capital/lowercase letters, spelling mistakes, etc.

**Current nomenclature** Current nomenclature mainly follows Sierwald et al. (2026), and references to the papers in which this species’ name was synonymised and/or transferred to another genus.

**Remarks** Information on the current whereabouts of other type specimens, if they exist, and additional information (on the quality/condition of type specimens in the ZISP collection; discrepancies between the information on labels and that given in the original descriptions, etc.). If no supplementary information is available, this section is omitted.

The following notation is used: (/)—new line; (//)— separate data label; [ ]— editorial text or comment; {}— English translation of the original Russian text.

Abbreviations used: D— described; nr— near; R. — river; Res.— reserve; Vil.— village.

In all cases, the accession numbers for type samples are given in a shortened format; for instance, the number MYR_DIP_0000001 reflects the full content of the actual label such as Zoological Institute / St. Petersburg / MYR_DIP_0000001.

Museum acronyms used in the text:

**FMNH** Finnish Museum of Natural History, Helsinki, Finland (curator: Rodrigo Salvador);

**GMNH** Gothenburg Natural History Museum, Gothenburg, Sweden (curator: Charlotte Jonsson);

**HMNH** Hungarian Museum of Natural History, Budapest, Hungary (curator: Zoltán Korsós);

**IPEE** A.N. Severtsov Institute of Evolution and Ecology of the Russian Academy of Sciences, Moscow, Russia (former curator: Sergei Golovatch); presently merged with the ZMMU collection;

**MIIZ** Museum and Institute of Zoology, Polish Academy of Sciences, Warsaw, Poland (former curator: Jolanta Wytwer);

**MNHG** Muséum d'histoire naturelle, Gèneve, Switzerland (curator: John Hollier);

**MNHN** Muséum national d'Histoire naturelle, Paris, France (former curator: Jean-Jacques Geoffroy);

**NHMD** Natural History Museum of Denmark, Copenhagen University, Denmark (curator: Nikolaj Scharff);

**NMNH** The Rijksmuseum van Natuurlijke Historie (= National Museum of Natural History), Leiden, Netherlands (curator: Jeroen Goud);

**SMF** Naturmuseum und Forschungsinstitutt Senckenberg, Frankfurt am Main, Germany (curator: Peter Jäger);

**SMNPS** Slovak Museum of Nature Protection and Speleology, Liptovský Mikuláš, Slovakia (curators: Ján Lakota and Andrej Mock);

**ZISP** Zoological Institute of the Russian Academy of Sciences, St. Petersburg, Russia (curator: Dmitri Logunov);

**ZMB** Museum für Naturkunde in Berlin, Germany (curator: Jason Dunlop);

**ZMMU** Zoological Museum of the Moscow University, Moscow, Russia (curator: Arkady Schileyko).

## Type catalogue

### Order Callipodida

**Family Callipodidae**

***carinatum* Brandt, 1840 (*Lysiopetalum* )**

*Lysiopetalum carinatum*Brandt 1840b: 42 (D♀).

**Type. *Holotype***: (♀), MYR_DIP_0000030.

**Type locality**. Dalmatia, a coastal region and islands in modern-day Croatia along the Adriatic Sea coast (no exact locality).

**Label data**. *Lisiopetalum* / *carinatum* Brandt / Опр. {Det.} Brandt / Сбор {Collected} / holotype ♀ // *Lisiopetalum carinatum* / Brandt / holotype // Julus (Julus) crenatus (*crenatus*) Kllr. (Kllr.) Dalmat. (Dalmat).

**Current nomenclature**. *Acanthopetalum carinatum* (Brandt, 1840) (Sierwald et al. 2026). Transferred to the genus *Acanthopetalum* Verhoeff, 1900 by Strasser (1971).

**Remarks**. This species was described from the holotype ♀ alone, and it was described twice, by Brandt (1840b: 322) and Brandt (1841a: 42), in both cases as ‘*Lysiopetalum carinatum* Nob.’ and with the identical short text. The holotype is broken.

### Family Caspiopetalidae

***oblonga* Golovatch, 1979 (*Bollmania* )**

*Bollmania oblonga* Golovatch, 1979a: 990, 1.13–15 (D♂♀).

**Types. *Holotype***: (♂), MYR_DIP_0000110. ***Paratypes***: (3♀), same number.

**Type locality**. Tajikistan, ca 31 km N of Dushanbe, Kondara Canyon (ca 38°48'N, 68°49'E).

**Label data**. *Bollmania* / *oblonga* Golovatch / det. С. Головач {S. Golovatch} / Сбор {Collected} Таджикистан, Варзобский / р-н, ущелье Кондары (напротив / на лев. берегу р. Варзоб) {Tajikistan, Varzob Distr., Kondary Canyon (opposite on the left bank of Varzob River)}: 29.IV.1944 / leg. Е.С. Кирьянова E.S. Kir’yanova} / голотип / holotype / 21 // № 71–1947 // *Bollmania* / *oblonga* Golovatch / 1♂ голотип / (с препаратом) / 3♀ паратипы // №71–1947 / 28.IV.1944 г. № 10 / Таджикистан / Варзобский район / близ ущ. Кондары / (напротив на левом / берегу р. Варзоб) {Tajikistan, Varzob Distr., Kondary Canyon (opposite on the left bank of Varzob River)} / Сб. Е.С. Кирьяновой {E.S. Kir’yanova}.— Micropreparation: *Bollmania oblonga* / Golovatch 1♂ / голотип / holotypus / 21 / гоноподии {gonopods} // Таджикистан / Варзобский р-н / близ ущелья Кондары / (напротив на левом / берегу р. Варзоб) {Tajikistan, Varzob Distr., Kondary Canyon (opposite on the left bank of Varzob River)} / 28.IV.1944 / leg. Е.С. Кирьянова {E.S. Kir’yanova} / (колл. ЗИН № 71–1947) // ЗИН № 71–1947 // № 21 // 0000110.

**Current nomenclature**. *Bollmania oblonga* Golovatch, 1979 (Sierwald et al. 2026).

**Remarks**. The entire type series is in ZISP.

***nodifrons* Lohmander, 1933 (*Bollmania* )**

*Bollmania nodifrons*Lohmander 1933: 49, figs 22, 28–29, 45–47 (D♂).

**Type. *Holotype***: (♂), MYR_DIP_0000268.

**Type locality**. Uzbekistan, Baba-Tau Mts [= Bobotog Mts] (ca 37°59'N, 68°10'E).

**Label data**. Зоол. Муз. АН {Zoological Museum of the Academy of Sciences} / №90–97 г. / 574. *Bollmania* / *nodifrons* Lohm. / Опр. {Det.} spec. orig. ♂ / Сбор {Collected} Бухара, Баба- / тау {Bukhara, Baba-Tau [Mts]} 1897 г. / А. Казнаков {A. Kaznakov} // А. Казнаков {A. Kaznakov} / Баба-Тау {Baba-Tau [Mts]} / [on the opposite side of the label]: №90–97 // *Caspiopetalum nodifrons* / n.sp. / Hans Lohmander.

**Current nomenclature**. *Bollmania nodifrons* (Lohmander, 1933) (Sierwald et al. 2026). Transferred to the genus *Bollmania* Silvestri, 1896 by Lohmander (1933: 44).

**Remarks**. The species was described from the holotype alone. Although this species name was first introduced by Lohmander (1931: 5), who not only referred to its terra typica (as ‘aus Bukhara stammend’), but also provided an illustration of its gonopod in order to compare with *B. orientalis* Silvestri, 1895 (Ibid.: fig. 2), it was later considered a nomen nudum (Stoev and Enghoff 2005). This is why the reference to Lohmander (1933) is provided here as the original description of this species.

***serrata* Lohmander, 1933 (*Bollmania* )**

*Bollmania serrata* Lohmander, 1933: 47, fig. 3 (D♂).

**Types. *Syntypes***: (2♂ 1♀), MYR_DIP_0000260.

**Type locality**. Uzbekistan, Shar-Dary Vil. [= Sharshara Vil.] (ca 39°30'N, 66°38'E).

**Label data**. Зоол. Муз. АН {Zoological Museum of the Academy of Sciences} / №90–97 г. / 577. *Bollmania* / *serrata* Lohm. / Опр. {Det.} spec. orig. 2♂ / Сбор {Collected} Бухара, дер. / Шах-дары, {Bukhara, Shakh-Dary Vil.} 1897 г. / А. Казнаков {A. Kaznakov} // Дер. Шар-Дары {Shar-Dary Vil.} / А. Казнаков {A. Kaznakov} / [on the opposite side of the label]: №90–97 // *Caspiopetalum serratum* / n.sp. / Hans Lohmander.

**Current nomenclature**. *Bollmania serrata* Lohmander, 1932 (Sierwald et al. 2026).

**Figure 3.**
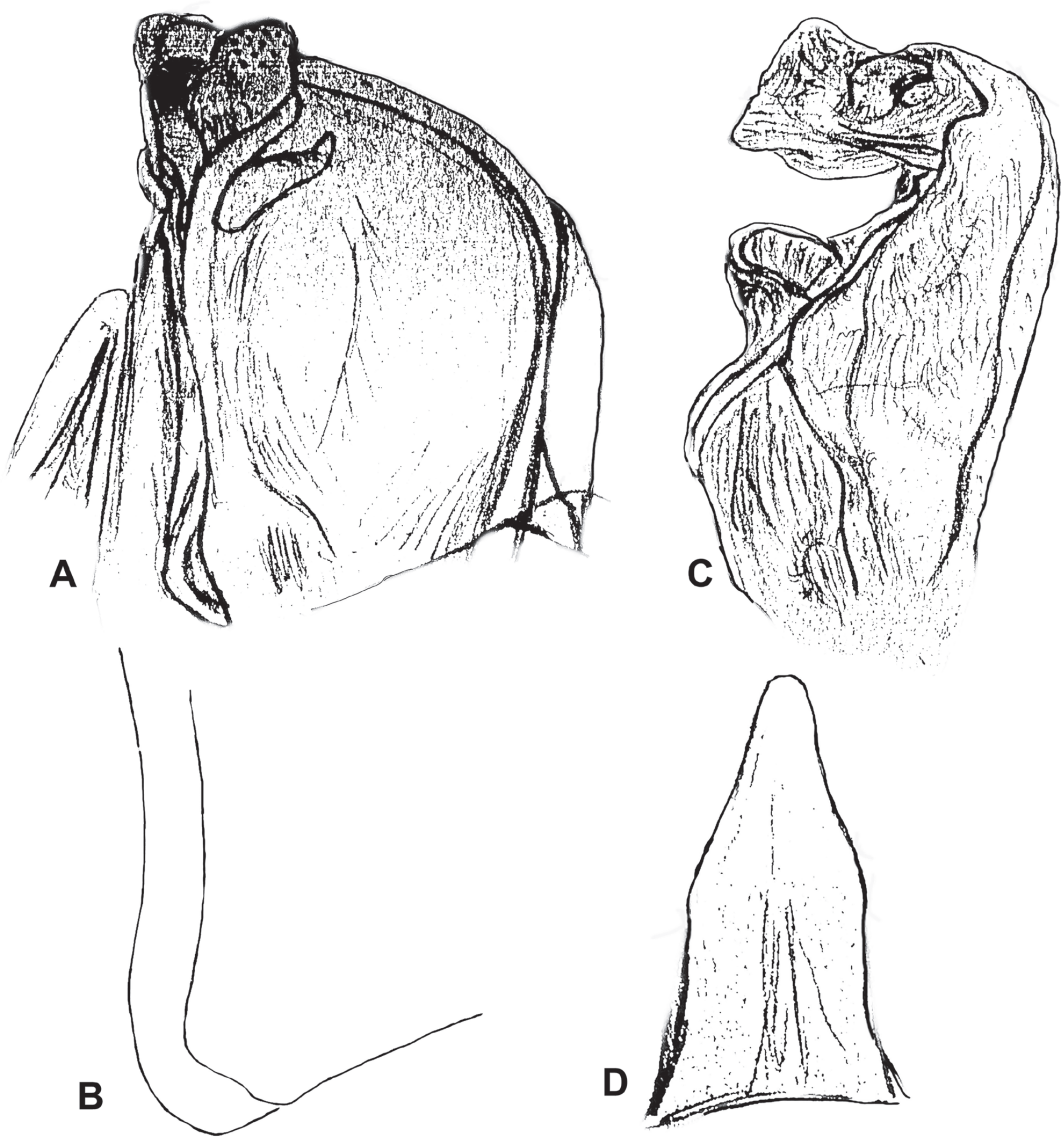
*Centrobolus elegans* (Brandt, 1840) (= Julus (Spirobolus) elegans Brandt, 1840), ♂ lectotype with 42 body rings + telson. **A**. Left anterior gonopod, caudal view; **B**. Ventro-lateral corner of collum; **C**. Right posterior gonopod, oral view; **D**. Sternum of anterior gonopods. Drawn not to scale. SIG del.

**Remarks**. Although the label states that 2♂ are available in this sample, actually there are three specimens inside the sample tube: two are devoid of heads, and the third specimen seems to be a female. Yet, the original description (Lohmander 1933: 47) was based on two males only, omitting the corresponding female. As with *B. nodifrons*, this name was first introduced by Lohmander (1931: 5), who also referred to its terra typica (as ‘aus Bukhara stammend’) and provided illustrations of the gonopods (Ibid.: fig. 3). However, this original introduction does not correspond to the requirements of availability (ICZN 1999: article 13.1) and is to be considered a nomen nudum.

### Order Chordeumatida

**Family Entomobielziidae**

***ornata* Golovatch, 1979 (*Tianella* )**

*Tianella ornata* Golovatch, 1979a: 987, figs 1.1–12 (D♂♀).

**Types. *Holotype***: (♂), MYR_DIP_0000100. ***Paratypes***: (1 juv.), MYR_DIP_0000102; (1♂ 1♀ 5 juv.), MYR_DIP_0000103; (1♂ 1♀ 1 juv.), MYR_DIP_0000104; (2♂ 7♀ 13 juv.), MYR_DIP_0000105.

**Type locality**. Kazakhstan, the coordinates are given for the modern Ranger Station Dzhalansh situated in the valley of Zhalanash River (ca 45°29'N, 80°31'E).

**Label data**. MYR_DIP_0000100: *Tianella* / *ornata* Golovatch / № 232–1937 / Опр. {Det.} det. Головач С.И. {S.I. Golovatch} / Сбор {Collected} Восточный Казахстан, / Джунгарский Алатау, / долины рек Джаланаш и / Теректы {East Kazakhstan, Dzhungarian Alatau Mt. Range, valleys of Dzhalanash and Terekty rivers}: 16–22.VIII.1936 / leg. В.Н. Шнитников {V.N. Shnitnikov} / голотип / holotype / 20 // *Tianella ornata* / Golovatch / ♂ голотип / holotypus // Дол. р. Джаланаш / и Теректы, Джунг. / Алатау {valleys of Dzhalansh and Terekty rivers, Dzhungarian Alatau Mt. Range}, 16–22.VIII.1936 / leg. В. Шнитников {V. Shnitnikov} / № 232–1937 // голотип № 20 {holotype} / Diplopoda.

MYR_DIP_0000102: *Tianella ornata* / Golovatch, 1979. / Опр. {Det.} С. Головач {S. Golovatch} / Сбор {Collected} В.Н. Шнитников {V.N. Shnitnikov} / Джунгарский Алатау {Dzhungarian Alatau Mt. Range} / паратипы {paratypes} // *Tianella ornata* / Gol. / 1 juv. Паратип {paratype} // Jus. р. Теректы. / Джунг. Алатау {Terekty River, Dzhungarian Alatau Mt. Range} / нач. {beginning} VIII.1936 г. / В. Шнитников {V. Shnitnikov} / № 232–1937 // 7.

MYR_DIP_0000103: *Tianella ornata* / Golovatch, 1979 / Опр. {Det.} С. Головач {S. Golovatch} / Сбор {Collected} В.Н. Шнитников {V.N. Shnitnikov} / Джунгарский Алатау {Dzhungarian Alatau Mt. Range} / паратипы {paratypes} // *Tianella ornata* / Golovatch. / паратипы / paratypes. 1 ♂ (+ препараты), / 1♀, 5 juv // Дол. р. Джала- /наш Джунгар. / Алатау {valley of Dzhalanash River, Dzhungarian Alatau Mt. Range}, 16–17.VIII. / 1936 г. / В. Шнитников {V. Shnitnikov} / № 232–1937 // 1♂ 1♀, 5 juv.— Micropreparation: *Tianella ornata* / Golovatch. 1 ♂ паратип / гоноподии, ноги // Казахстан / Джунгарский Алатау / долина р. Джаланаш {Kazakhstan, Dzhungarian Alatau Mt. Range, valley of Dzhalansh River} / 16–17.III.1936 / leg. В.Н. Шнитников {V.N. Shnitnikov} / (колл. ЗИН № 232–1937 / 0000103.

MYR_DIP_0000104: *Tianella ornata* / Golovatch, 1979 / Опр. {Det.} С. Головач (S. Golovatch} / Сбор {Collected} В.Н. Шнитников {V.N. Shnitnikov} / Джунгарский Алатау {Dzhungarian Alatau Mt. Range} / паратипы {paratypes} // *Tianella ornata* / Golovatch / паратипы {paratypes}, 1♂ 1♀, 1 juv. // Дол. р. Теректы / Джунг. Алатау {valley of Terekty River, Dzhungarian Alatau Mt. Range} / 11–16. VIII.1936. / В. Шнитников {V. Shnitnikov} / № 232–1937.

MYR_DIP_0000105: *Tianella ornata* / Golovatch, 1979. / Опр. {Det.} С. Головач. / Сбор {Collected} В.Н. Шнитников {V.N. Shnitnikov} / Джунгарский Алатау {Dzhungarian Alatau Mt. Range} / паратипы // *Tianella ornata* / Golovatch. / Паратипы {paratypes} / paratypes, 2♂ (+ препараты {preparations}), / 7♀, 13 juv. // Дол. р. Джала- / наш и Теректы / Джунг. Алатау {valleys of Dzhalansh and Terekty rivers, Dzhungarian Alatau Mt. Range} / 16–22. VIII.1936 г. / В. Шнитников {V. Shnitnikov} / № 232–1937.— Micropreparation: *Tianella ornata* / Golovatch. 1 ♂ паратип / гоноподии // Казахстан / Джунгарский Алатау / долина р. Джаланаш / и Теректы {Kazakhstan, Dzhungarian Alatau Mt. Range, valley of Dzhalanash and Terekty Rivers}, 16–17.III.1936 / leg. В.Н. Шнитников {V.N. Shnitnikov} / (колл. ЗИН № 232–1937 / 105.

**Current nomenclature**. *Tianella ornata* Golovatch, 1979 (Sierwald et al. 2026).

**Remarks**. The entire type series is in ZISP.

### Family Kashmireumatidae

***topali* Golovatch, 1984 (*Vieteuma* )**

*Vieteuma topali* Golovatch, 1984b: 74, figs 36–45 (D♂♀).

**Type. *Paratype***: (1♂), MYR_DIP_0000135.

**Type locality**. Vietnam, pass between Lao Cai and Lai Chau provinces (ca 22°21'N, 103°46'E).

**Label data**. 1♂ паратип / *Vieteuma topali* / Golovatch / Опр. {Det.} Golovatch / Сбор {Collected} Vietnam, № 117, / leg. Topál-Matskási, / 1971 // Vietnam, № 117, / 1971, leg. Topál-Matskási // *Vieteuma topali* / Golovatch. n. sp. / 1♂ paratype / Паратип {paratype} / Взято за обработку / из Будапештского / музея ест. истории {retained from the Budapest Natural History Museum for help with ID}.

**Current nomenclature**. *Vieteuma topali* Golovatch, 1984 (Sierwald et al. 2026).

**Remarks**. The holotype ♂ and all other paratypes (6♂ 2♀) are in HMNH.

### Family Megalotylidae

***vietnamica* Golovatch, 1984 (*Nepalella* )**

*Nepalella vietnamica* Golovatch, 1984a: 123, figs 1–6 (D♂♀).

**Types. *Paratypes***: (1♂ 1♀), MYR_DIP_0000124.

**Type locality**. Vietnam, Yên Bái Prov., Lục Yên Distr. (ca 22°10'N, 104°38'E).

**Label data**. *Nepalella vietnamica* Golovatch / 1♂ 1♀ паратипы {paratypes} / Опр. {Det.} / Сбор {Collected} Vietnam, Prov. Jen Bai / № 238, leg. Topál-Matskási, 1971 // *Nepalella vietnamica* / Golovatch, 1982 / 1♂ 1♀ паратипы / paratypes / Взято за обработку из / коллекции Венгерского / музея ест. истории / (Будапешт) {retained from the Hungarian Natural History Museum (Budapest) for help with ID}, 1981 // Vietnam, № 238 / 1971, / leg. Topál-Matskási.

**Current nomenclature**. *Nepalella vietnamica* Golovatch, 1984 (Sierwald et al. 2026).

**Remarks**. The holotype ♂ and all other paratypes (3♂ 7♀) are in HMNH.

### Family Metopidiotrichidae

**melanocephala Golovatch, 1984 (*Metopidiothrix* )**

*Metopidiothrix melanocephala* Golovatch, 1984b: 71, figs 3–35 (D♂♀).

**Types. *Holotype***: (♂), MYR_DIP_0000140. ***Paratypes***: (1♂ 1♀ 2 juv.), MYR_DIP_0000138; (1♂ 1♀ 4 juv.), MYR_DIP_0000139; (1♀), MYR_DIP_0000141.

**Type locality**. Vietnam, Gia Lai – Kon Tum Prov., 35 km N of Ah Khe Town, Buon Luoi Vil. (ca 14°20'N, 108°36'E).

**Label data**. MYR_DIP_0000140: *Metopidiothrix* / *melanocephala* / Golovatch n. sp. / 1♂ голотип / holotype // Вьетнам, Тай-Нгуен, / Буон-Лыой, 35 км сев. / Анкхе, дожд. троп. / лес, подст. {Vietnam, Tay-Nguyen [Plateau], Buon Luoi [Vil.], 35 km N of An Khe [Town], tropical rain forest, litter}, 4.1.1981 / г. leg. А.Я. Друк {A.Ya. Druk} // Vietnam, Prov. Thai Nguejen, / Buon luoi [Vil.], 35 km N An Khe [Town], / tropical rain forest, litter, / 4 January 1981, leg. A. Druk.

MYR_DIP_0000138: паратипы {paratypes} / *Metopidiothrix* / *melanocephala* / Golovatch. n. sp. / 1♂ 1♀, 2 juv. / ♂ dissected // Вьетнам, Тай-Нгуен, / Буон-Лыой, 35 км сев. / Анкхе, троп. лес, пр. 9 / 1 м^2^ подст. {Vietnam, Tay-Nguyen [Plateau], Buon Luoi [Vil.], 35 km N of An Khe [Town], tropical forest sample 9, litter}, 4.I.81 Друк {Druk}.— Micropreparation: *Metopidiothrix* / *melanocephala* n. sp. / 1 ♂ paratype / det. S. Golovatch, 1982 / Vietnam, Thai Nguyen / Prov., Buon luoi [Vil.], 35 km N / An Khe [Town], tropical forest, / 4.1.1981, leg. A. Druk // № 2 // 0000_138.

MYR_DIP_0000139: *Metopidiothrix* / *melanocephala* Gol. / 1♂ 1♀, 4 juv. / паратипы / paratypes / ♂ dissected // Вьетнам, Тай-Нгуен, / Буон-Лыой, 35 км сев. / Анкхе, троп. лес {Vietnam, Tay-Nguyen [Plateau], Buon luoi, 35 km N of An Khe [Town], tropical forest}, А0. / пр. 1 {sample 1}, 1 м^2^, 30.XII.1980 / Сергеева {Sergeeva}.— Micropreparation: *Metopidiothrix* / *melanocephala* n. sp. / 1♂ paratype–паратип / det. С.И. Головач {S.I. Golovatch} / Вьетнам, пров. Тай / Нгуен, 35 км сев. Ан- / кхе, Буон-Лыой, троп. / лес. {Vietnam, Prov. Thai Nguejen, Buon Luoi [Vil.], 35 km N of An Khe [Town], tropical forest}, 30.XII.1980, Серг. {Sergeeva} // *Metopidiothrix* // № 1 // 0000_139.

MYR_DIP_0000141♀: *Metopidiothrix* / *melanocephala* / Golovatch. n. sp. / 1♀ паратип / paratype // Вьетнам, Тай-Нгуен, / Буон-Лыой, 35 км сев. Анкхе, / троп. лес, пр. 1.24, 1/6 м^2^, подст. {Vietnam, Tay-Nguyen [Plateau], Buon Luoi [Vil.], 35 km N of An Khe [Town], tropical forest, sample 1.24, litter} / 9.1.1981, Сергеева {Sergeeva}.

**Current nomenclature**. *Metopidiothrix melanocephala* Golovatch, 1984 (Sierwald et al. 2026).

**Remarks**. The remaining paratypes are in HMNH (1♂ 1♀). Although Golovatch (1984b: 71) mentioned that some paratypes would be transferred to ZMMU, this never happened. Even though the original labels stated that the samples were collected in Vietnam’s province of ‘Thai Nguejen’, the actual collecting locality lies in An Khe District of Gia Lai – Kon Tum Province. The actual Province Thai Nguyen (correct spelling!) lies just north of Hanoi.

***simile* Golovatch, 1986 (*Australeuma* )**

*Australeuma simile*Golovatch 1986b: 257, fig. 3A–G (D♂♀).

**Types. *Paratypes***: (1♂ 1♀), MYR_DIP_0000144.

**Type locality**. Tasmania, Cynthia Bay (ca 42°07'S, 146°10'E).

**Label data**. *Australeuma simile* / Golovatch, 1986 / Опр. {Det.} С. Головач {S. Golovatch} / C.A.W. & A.M. JEEKEL / Tasmania /1♂ 1♀ паратипы / paratypes // *Australeuma simile* / Golovatch, n. sp. / 1♂ 1♀ paratypes / паратипы / взято за обработку / С.И. Головач, 1984 {taken for identification, S. Golovatch, 1984} // Tasmania, Lake St. Clair / National Park, near Cynthia / Bay, 5 km WNW of Derwent / Bridge, wet *Eucalyptus* forest / under logs, leg. C.A.W. & A.M. JEEKEL. / Zoölogisch Museum Australia / Expedition 1980 / 26.XI.1980.

**Current nomenclature**. *Australeuma simile* Golovatch, 1986 (Sierwald et al. 2026).

**Remarks**. The holotype ♂ and all other paratypes (4♂ 2♀ 3 juv.) are in NMNH.

### Order Glomerida

**Family Glomeridae**

***awchasica* Brandt, 1840 (*Glomeris* )**

*Glomeris awchasica* Brandt, 1840a: 42.

**Types. *Holotype***: (1♀), MYR_DIP_0000018.

**Type locality**. Apparently Abkhazia (no exact locality).

**Label data**. *Glomeris awchasica* / Brandt / Опр. {Det.} Brandt / Сбор {Collected} Nordmann, Awcharia / Holotype / голотип // Awhasia / ad. lastellun / Gelendjetrick(?) [label illegible].

**Current nomenclature**. *Hyleoglomeris awchasica* (Brandt, 1840) (Sierwald et al. 2026). Transferred to the genus *Hyleoglomeris* Verhoeff, 1910 by Golovatch (1989).

**Remarks**. The type locality remains a bit unclear. Brandt (1840a: 42) wrote that this specimen had been collected ”au voyage de M. le prof. Normann à Odessa un individu de *Glomeris* recueillei par lui en Awhasie” – apparently in Abkhazia (see Golovatch 1989; Golovatch and Hoffman 2000). However, although the original label is rather illegible, it contains the word ‘Gelendjetrick’, which could be interpreted as Gelendzhik (ca 44°34'N, 38°05'E), a city in the Krasnodar Territory of Russia.

***conspicua* Golovatch, 1983 (*Hyperglomeris* )**

*Hyperglomeris conspicua* Golovatch, 1983: 110, figs 11–14 (D♂♀).

**Types. *Holotype***: (♂), MYR_DIP_0000136. ***Paratypes***: (5♀), MYR_DIP_0000137.

**Type locality**. Vietnam, Hoa-Binh Prov., Vạn Mai Vil. (ca 20°35'N, 105°02'E).

**Label data**. MYR_DIP_0000136: *Hyperglomeris conspicua* / Golovatch. / Опр. {Det.} Golovatch / Сбор {Collected} Вьетнам, пров. Хоа-бинь, / уезд Май-Тяо, дер. Вон-Май, / вторичн. тропич. лес. 7.XII.1981. {Vietnam, Hoa-Binh Prov., Mai Châu Distr., Vạn Mai Vil., secondary tropical forest} / leg. Д. Криволуцкий и Л. Рыбалов {leg. D. Krivolutsky and L. Rybalov} / голотип ♂ {holotype} / паратипы 5♀ {paratypes} // Вьетнам, пров. Хоа-бинь, / уезд Май-Тяо, дер. Вон-Май, / вторичный тропический лес {Vietnam, Hoa-Binh Prov., Mai Châu Distr., Vạn Mai Vil., secondary tropical forest} / 6.XII.1981, leg. Д.А. Криволуцкий / и Л.Б. Рыбалов {leg. D.A. Krivolutsky and L.B. Rybalov} // *Hyperglomeris conspicua* Golovatch 1♂ голотип holotype. det. С. Головач {S. Golovatch}, 1982.

MYR_DIP_0000137: *Hyperglomeris conspicua* / Golovatch. / Опр. {Det.} Golovatch. / Сбор {Collected} Вьетнам, пров. Хоа-бинь, / уезд Май-Тяо, дер. Вон-Май, / вторичн. тропич. лес. 7.XII.1981. {Vietnam, Hoa-Binh Prov., Mai-Tyao Co, Vạn Mai Vil., secondary tropical forest} / leg. Д. Криволуцкий и Л. Рыбалов {leg. D. Krivolutsky and L. Rybalov} / голотип ♂ {holotype} / паратипы 5♀ {paratypes} // Вьетнам, Пров. Хоа-бинь, / уезд Май-Тяо, дер. Вон-Май, / Вторичн. тропич. лес, {Vietnam, Hoa-Binh Prov., Mai Châu Distr., Vạn Mai Vil., secondary tropical forest} / 7.XII.1981, leg. Д. Криволуцкий, / Л. Рыбалов {leg. D. Krivolutsky and L. Rybalov} / *Hyperglomeris conspicua* / Golovatch 5♀, паратипы. paratypes. / det. С. Головач, 1982.— Micropreparation: *Hyperglomeris* / *conspicua* Golovatch / 1♂ паратип paratype / пары ног {leg pairs} 17–19 / Вьетнам, пров. Хоа-Бинь / уезд Май-Тяо, дер. Вон-Май / вторичный тропический лес, {Vietnam, Hoa-Binh Prov., Mai Châu Distr., Vạn Mai Vil., secondary tropical forest} / 6.XII.1981, leg. Криволуцкий / и Рыбалов {leg. Krivolutsky and Rybalov} // № 4 // 0000137.

**Current nomenclature**. *Hyperglomeris conspicua* Golovatch, 1983 (Sierwald et al. 2026).

**Remarks**. The remaining paratypes are in ZMMU (3♂ 11♀), HMNH (1♂ 1♀), and NHMD (1♀).

***cremea* Golovatch, 1983 (*Hyleoglomeris* )**

*Hyleoglomeris cremea* Golovatch, 1983: 114, figs 23–25 (D♂♀).

**Type. *Paratype***: (1♂), MYR_DIP_0000145.

**Type locality**. Thailand, Chiang Dao (ca 19°24'N, 98°50'E).

**Label data**. *Hyleoglomeris cremea* / Golovatch / Опр. С. Головач {Det. S. Golovatch} / N Thailand / Chiang Dao, 1800 m / 1958–59, leg. Birgit / Degerbøl // *Hyleoglomeris cremea* Golovatch / N. Thailand: Chiang Dao 1800 m / 1958–59. Birgit Degerbøl / S. Golovatch det., 1982 / Zool. Mus. København ded. 1983 // 1♂ paratype.

**Current nomenclature**. *Hyleoglomeris cremea* Golovatch, 1983 (Sierwald et al. 2026).

**Remarks**. The holotype ♂ and several paratypes (1♂ 4♀) are in NHMD, and one paratype (1♀) in HMNH.

***koreana* Golovatch, 1978 (*Hyleoglomeris* )**

*Hyleoglomeris koreana* Golovatch, 1978: 678, figs 2.1–5 (D♂♀).

**Types. *Holotype***: (♂), MYR_DIP_0000125. ***Paratypes***: (1♂ 2♀), MYR_DIP_0000108.

**Type locality**. South Korea, S of Gangneung-si (= Kannyn) (ca 37°41'N, 128°54'E).

**Label data**. MYR_DIP_0000125: голотип ♂ /№ 250-02 / *Hyleoglomeris* / *koreana* Golovatch. Опр. {Det.} S.I. Golovatch / Сбор {Collected} Южная Корея {South Korea}/ leg. П.Ю. Шмидт {P.Yu. Schmidt} / 20.VII.1900 // *Hyleoglomeris* / *koreana* Golovatch / голотип ♂ / det. С. Головач {S.I. Golovatch} 1975 // Корейско-Сахалинская / экспедиция 1900–01 гг. / Южная Корея, Олочонса / (южнее Каннына) / leg. П.Ю. Шмидт {Korean-Sakhalin expedition 1900–01, South Korea, Olochonsa (S of Kannyn), leg. P.Yu. Schmidt} / 20.VII.1900.— Micropreparation: *Hyleoglomeris* / *koreana* Golovatch. / ♂ (телоподии) {telopods} / голотип {holotype} / Корейско-Сахалинская / эксп. П.Ю. Шмидта. Южная Корея, Олочонса (южнее Канны- / на) 20.VII.1900 {Korean-Sakhalin by P.Yu. Schmidt, South Korea, Olochonsa (S of Kannyn)} // 0000125.

MYR_DIP_0000108: *Hyleoglomeris* / *koreana* Golovatch / № 250-02 / Опр. {Det.} S.I. Golovatch / Сбор {Collected} Южная Корея / leg. П.Ю. Шмидт {leg. P.Yu. Schmidt} / 20.VII.1900 // Корейско-Сахалинская / экспедиция 1900–01 гг. / Олочонса (южнее / Каннына (Южная Корея)) leg. П.Ю. Шмидт {Korean-Sakhalin expedition 1900–01, South Korea, Olochonsa (S of Kannyn), leg. P.Yu. Schmidt} / 20.VII.1900 // *Hyleoglomeris koreana* / Golovatch / паратипы {paratypes}: 2♀, 1♂. / det. С. Головач 1975.

**Current nomenclature**. *Hyleoglomeris koreana* Golovatch, 1978 (Sierwald et al. 2026).

**Remarks**. The entire type series is in ZISP.

***maxima* Golovatch, 1983 (*Hyperglomeris* )**

*Hyperglomeris maxima* Golovatch, 1983: 108, figs 6–10 (D♂).

**Types. *Holotype***: (♂), MYR_DIP_0000112. ***Paratype***: (1♂), MYR_DIP_0000111.

**Type locality**. Vietnam, Hoa-Binh Prov., Vạn Mai Vil. (ca 20°35'N, 105°02'E).

**Label data**. MYR_DIP_0000112: *Hyperglomeris maxima* / Golovatch / Опр. {Det.} Golovatch. / Сбор {Collected} Вьетнам, пров. Хоа-Бинь / уезд Май-Тяо, дер. Вон-Май / вторичн. тропич. лес. 12.XII.1981 {Vietnam, Hoa-Binh Prov., Mai-Tyao Co, Vạn Mai Vil., secondary tropical forest}/ leg. Д. Криволуцкий и Л. Рыбалов {D. Krivolutsky and L. Rybalov} / ♂ голотип. / ♂ паратип // *Hyperglomeris maxima* sp. n. / 1 ♂ голотип. holotype. / det. С. Головач, 1982 // Вьетнам, пров. Хоа-бинь, / уезд Май-Тяо, дер. Вон-Май, / вторичный тропический лес. / 12.XII.1981. leg. Д.А. Криволуцкий, / Л.Б. Рыбалов {Vietnam, Hoa-Binh Prov., Mai-Tyao Co, Vạn Mai Vil., secondary tropical forest, leg. D. Krivolutsky and L. Rybalov}.

MYR_DIP_0000111: *Hyperglomeris maxima* / Golovatch / Опр. {Det.} Golovatch. / Сбор {Collected} Вьетнам, пров. Хоа-Бинь / уезд Май-Тяо, дер. Вон-Май / вторичн. тропич. лес. 12.XII.1981 {Vietnam, Hoa-Binh Prov., Mai-Tyao Co, Vạn Mai Vil., secondary tropical forest} / leg. Д. Криволуцкий и Л. Рыбалов {D. Krivolutsky and L. Rybalov} / ♂ голотип {holotype} / ♂ паратип (paratype} // *Hyperglomeris maxima* / sp. n. / 1♂ паратип. paratype. / Вьетнам, пров. Хоа-Бинь, уезд / Май-Тяо, дер. Вон-Май, вторичн. / тропич. лес. 12.XII.1981. leg. / Д.А. Криволуцкий и Л.Б. Рыбалов {Vietnam, Hoa-Binh Prov., Mai-Tyao Co, Vạn Mai Vil., secondary tropical forest, leg. D. Krivolutsky and L. Rybalov}.— Micropreparation: *Hyperglomeris* / *maxima* Golovatch / 1♂ паратип paratype / пары ног 17–19 {leg pairs 17–19) / Вьетнам, пров. Хоа-Бинь, / уезд Май-Тяо, дер. Вон-Май / вторичный тропич. лес, / 12.XII.1981. leg. Криволуц- / кий и Рыбалов {Vietnam, Hoa-Binh Prov., Mai-Tyao Co, Vạn Mai Vil., secondary tropical forest, leg. D. Krivolutsky and L. Rybalov} // № 3 // 0000111.

**Current nomenclature**. *Hyperglomeris maxima* Golovatch, 1983 (Sierwald et al. 2026).

**Remarks**. The remaining paratypes are in ZMMU (1♂) and HNHM (1♂). The paratype (MYR_DIP_0000111) dried out and was moistened in lactic acid on 11.V.2023.

***montana* Golovatch, 1983 (*Hyleoglomeris* )**

*Hyleoglomeris montana* Golovatch, 1983: 112, figs 16–19 (D♂♀).

**Type. *Paratype***: (1♀), MYR_DIP_0000106.

**Type locality**. Thailand, Chiang Mai Prov., Doi Inthanon (ca 18°35'N, 98°29'E).

**Label data**. *Hyleoglomeris montana* / Golovatch / Опр. {Det.} Golovatch. / Сбор {Collected} N Thailand / Chiang Mai Prov., Doi Inthanon, / summit, 2500 m, 7.X.1981. / Zool. Muz. Copenhagen leg. / 1 ♀ паратип // Thailand: Chiang Mai Province, / Doi Inthanon, summit, / 2500 m, 7.X.1981 / Zool. Museum Copenhagen leg. // *Hyleoglomeris montana* Golovatch / ♀ paratype. S. Golovatch det., 1982. / Zool. Mus. København leg. 1983.

**Current nomenclature**. *Hyleoglomeris montana* Golovatch, 1983 (Sierwald et al. 2026).

**Remarks**. The holotype ♂ and the remaining paratypes (1♂ 3♀ 1 juv.) are in NHMD.

### Order Julida

**Family Blaniulidae**

***ammonites* Enghoff, 1984 (*Nopoiulus* )**

*Nopoiulus ammonites* Enghoff, 1984: 405, figs 12–19 (D♂♀).

**Types. *Holotype***: (♂), MYR_DIP_0000091. ***Paratypes***: (2♂ 8♀ 4 juv.♂ 1 juv.♀ 1 juv. asex), MYR_DIP_0000092.

**Type locality**. Russia, Krasnodar Territory, nr Shedok (ca 44°13'N, 40°51'E).

**Label data**. MYR_DIP_0000091: *Nopoiulus* (*Nopoiulus*) / *ammonites* Enghoff. / Опр. {Det.} Enghoff / Сбор {Collected} Краснодарский край, / Лабинский р-н, пещера Аммони- / товая, близ Щедока, окр. Псебая {Krasnodar Territory, Laba Distr., Ammonitovaya cave, nr Shedok, vicinity of Psebai}, / 4.VIII.1970, С.И. Лёвушкин и М. Снетков {[col.] S.I. Ljovuschkin and M. Snetkov} // Аммонитовая {Ammonitovaya} / 4.VIII.70 // USSR, Ciscaucasia, / Krasnodar Territory, / Laba region, Cave / Ammonitovaya / near Shedok, / Psebai district / 4.VIII.1970 / leg. S.I. Ljovuschkin & / M. Snetkov // the material was once dried! // *Nopoiulus (Nopoiulus) ammonites* n. sp. / ♂ HOLOTYPE / H. Enghoff det., 1982 // ♂ 34+2+T, L 12 mm, H 0, 80 mm, / sgm. XVII: 0,80 × 0,71 × 0,59 mm, / ca. 8 setae, L 0,04 mm. / P 1–2, left P 9, præp. G-11. / ant. P 28–29, præp. G-12.— Micropreparations (×2): *Nopoiulus ammon*- / *ites* ♂ HOLOTYPE / P 1-2, left P9 / Euparal // Præp. G-11 / H. Enghoff // Ciscaucasia, / Krasnodar Terr. / Ammonitovaya Cv. / 4.VIII.1970, Ljo- / vuschkin & Snetkov // 00000_91. – *Nopoiulus ammon*- / *ites* ♂ HOLOTYPE / right ant.-P28- / left ant.-P29, Euparal // Præp. G-12 H. Enghoff // Ciscaucasia / Krasnodar Terr. / Ammonitovaya Cv. / 4.viii.1970. Ljo- / vuschkin & Snetkov / № 22 // 00000_91.

MYR_DIP_0000092: *Nopoiulus (Nopoiulus)* / *ammonites* Enghoff. / Опр. {Det.} Enghoff / Сбор {Collected} Краснодарский край, / Лабинский р-н, пещера Аммони- / товая, близ Щедока, окр. Псебая {Krasnodar Territory, Laba Distr., Ammonitovaya cave, nr Schedok, vicinity of Psebai}, / 4. VIII.1970. С.И. Лёвушкин и М. Снетков {S.I. Ljovuschkin and M. Snetkov} // USSR, Ciscaucasia, Krasnodar Territory, / Laba region, Cave Ammonitovaya near by Shedok, / Psebai district. / 4.VIII.1970. / S.I. Ljovuschkin & M. Snetkov leg. // *Nopoiulus (Nopoiulus) ammonites* n. sp. / 2♂ 8♀, 4 j.♂ 1 j.♀, 1 j. asex. PARATYPES. / H. Enghoff det., 1982 // ♀, præp. G-30–31 – в маленькой пробирке {in small vial}.— Micropreparations (×2): *Nopoiulus ammon*- / *ites* ♀ PARATYPE / ♀P2, left vulva / Euparal // Præp. G-30 / H. Enghoff // Ciscaucasia: Kras- / nodar Terr. Ammo- / nitovaya Cave / 4.VIII.1970, Ljo- / vuschkin & Snetkov // 00000_92. – *Nopoiulus ammon*- / *ites* ♀ PARATYPE / ♀clypeus-mand. / right ant. -P36- / gnathoch.-left / ant.-P37.Euparal // Ciscaucasia: Kras- / nodar Terr. Ammo- / nitovaya Cave / 4.viii.1970, Ljo- / vuschkin & Snetkov / No.32 // Præp. G-31 / H. Enghoff // 00000_92.

**Current nomenclature**. *Nopoiulus ammonites* Enghoff, 1984 (Sierwald et al. 2026).

**Remarks**. The remaining paratypes are in ZMF (1♂ 1♀) and NHMD (1♂ 1♀).

***brevipilosus* Enghoff, 1984 (*Nopoiulus* )**

*Nopoiulus brevipilosus* Enghoff, 1984: 414, figs 19, 33–36, 54 (D♂).

**Type. *Holotype***: (♂), MYR_DIP_0000063.

**Type locality**. Abkhazia, nr Sukhumi, Kelasuri (= Aleksandrovskaya) cave (ca 43°01'N, 41°08'E).

**Label data**. *Nopoiulus (Paranopoiulus)* / *brevipilosus* Enghoff, 1983 / Опр. {Det.} Enghoff / Сбор {Collected} Абхазия, окрестн. Сухуми / пещера Келассурская {Abkhazia, nr Sukhumi, Kelassuri cave}, 23.IX.1969 / С.И. Левушкин leg. {S.I. Ljovuschkin} // Abkhasia / nr Sukhumi / Cave Kelassuri / leg. S.I. Ljovuschkin / 23.IX.1969— Micropreparations (×4): *Nopoilus brevipilo*- / *sus* Enghoff Holotype / P♂ oral, P2 abo- / ral, left ant., / left P9 lateral // Praep. G-5 / H. Enghoff // Abchasia, cave / Kelassuri near / Sukhumi. 23.ix. / 1969. Ljowusch- / kin. Euparal // 00000_63. – *Nopoilus brevipilo*- / *sus* Enghoff Holotype / ♂ right P9 mesal, / midbody leg. / No 25 // Abchasia, cave / Kelassuri near / Sukhumi. 23.ix. / 1969. Ljowusch- / kin. Euparal // Praep. G-6 / H. Enghoff // 00000_63. – *Nopoilus brevipi*- / *losus* Enghoff / ♂ P8 aboral / Holotype / No 26 // Praep. G-9 / H. Enghoff // Abchasia, cave / Kelassuri near / Sukhumi. 23.ix. / 1969. Ljowuschkin / 6–9 Euparal // 00000_63. – *Nopoilus brevipilo*- / *sus* Enghoff Holotype / ♂ P20 – gnathoch / P 21 Euparal / No 27 // Praep. G-16 / H. Enghoff // Abchasia, cave / Kelassuri near / Sukhumi. 23.ix. / 1969. Ljowuschkin // 00000_63.

**Current nomenclature**. *Nopoiulus brevipilosus* Enghoff, 1984 (Sierwald et al. 2026).

**Remarks**. The species was described from the holotype ♂ alone, which presently is fragmented into several pieces.

***coecus* Enghoff, 1984 (*Nopoiulus* )**

*Nopoiulus coecus* Enghoff, 1984: 416, figs 19, 37 (D♀).

**Type. *Holotype***: (♀), MYR_DIP_0000069.

**Type locality**. Armenia, Khosrov Forest Nature Res. (ca 40°02'N, 44°55'E).

**Label data**. *Nopoiulus (Paranopoiulus)* / *coecus* Enghoff / Опр. {Det.} Enghoff / Сбор {Collected} Армения, Хосровский / заповедник, смеш. широколиств. / лес. 8. V.1972. М.С. Гиляров / голотип ♀ // Caucasus, / Armenia, / Khosrov Reserv. / mixed broad- / leaved forest / 8.V.1972 / leg. M.S. Ghilarov // *Nopoiulus (Paranopoiulus) coecus* n. sp. / ♀ HOLOTYPE / H. Enghoff det., 1982 // præp. G-26–27— Micropreparations (×2): *Nopoiulus coecus* / Enghoff HOLOTYPE / ♀, P1, P2 (vestige- / al), right vulva / Euparal G-26 // Armenia: Khosrov / Reserv. 8.v.1972 / M.S. Ghilarov // Præp.: G-26 / H. Enghoff // № 9 / 00000_69. – *Nopoiulus coecus* / Enghoff ♀ HOLOTYPE / ♀ clypeus+anten- / nae-P42-gnatho- / chil.-P43 / Euparal G-27 // Armenia: Khasrov / Res. 8.V.1972 / M.S. Ghilarov // Præp.: G-27 / H. Enghoff // 00000_69.

**Current nomenclature**. *Nopoiulus coecus* Enghoff, 1984 (Sierwald et al. 2026).

**Remarks**. The species was described from the holotype ♀ only.

***densepilosus* Enghoff, 1984 (*Nopoiulus* )**

*Nopoiulus densepilosus* Enghoff, 1984: 411, figs 19, 24–26 (D♂♀).

**Types. *Holotype***: (♂), MYR_DIP_0000064. ***Paratypes***: (2♀), MYR_DIP_0000065.

**Type locality**. Azerbaijan, Zagatala State Nature Res. (ca 41°47'N, 46°33'E); the locality Agkemal was not found.

**Label data**. MYR_DIP_0000064: *Nopoiulus (Archinopoiulus)* / *densepilosus* Enghoff / Опр. {Det.} Enghoff / Сбор {Collected} Азербайджан, Закатальский зап-к, / Агкемаль {Azerbaijan, Zagatala Res., Agkemal} / 1800–2100 м, широколиственный лес {broad-leaved forest}, / leg. С. Головач и И. Мартенс {S. Golovatch and J. Martens} // 11 USSR Azerbaidzan, Zakatali / State Res. 1800–2100 m / Agkemal, 24–27 May 1981 / GOLOVATH & MARTENS leg. // clypeus-gnathochil – / P 44–45. præp. G-13 // right ant. P 1–2, / left P 9, præp. G-10 // *Nopoiulus (Archinopoiulus) densepilosus* n. sp. / ♂ HOLOTYPE // H. Enghoff det., 1982.— Micropreparations (×2): *Nopoiulus densepilo*- / *sus* Enghoff HOLOTYPE / ♂ Clypeus – P44 / (1) – gnathoch.- / P45 (2). Euparal // Præp.: G-13 / H. Enghoff // Azerbaidzan, Zaka- / tali State Res. / Agkemal 24–27.v. / 1981, Golovatch & Martens, G-13 // № 5 // 00000_64. – *Nopoiulus densepilo*- / *sus* Enghoff HOLOTYPE / ♂ right ant., P1 / oral, P2 aboral. / left P9 lateral / Euparal // Præp.: G-10 / H. Enghoff // Azerbaidzan, Zaka- / tali State Res. / Agkemal, 24–27.v. / 1981. Golovatch & Martens, G-10 // № 6 // 00000_64. –

MYR_DIP_0000065: *Nopoiulus (Archinopoiulus)* / *densepilosus* Enghoff / Опр. {Det.} Enghoff / Сбор {Collected} Азербайджан, Закатальский зап-к, / Агкемаль {Azerbaijan, Zakataly Distr., Agkemal} / 1800–2100 м, широколиственный лес {broad-leaved forest}, / leg. С. Головач и И. Мартенс {S. Golovatch and J. Martens} // *Nopoiulus (Archinopoiulus) densepilosus* n. sp. / 2 ♀ PARATYPES. H. Enghoff det., 1982 // præp. G-14, G-15 // 11 USSR Azerbaidzan, Zakatali / State Res., 1800–2100 m / Agkemal, 24–27 May 1981 / GOLOVATCH & MARTENS leg.— Micropreparations (×2): *Nopoiulus densepi*- / *losus* Enghoff / PARATYPE / ♀ gnathoch. / -right ant.-P40 (2) / left ant.-P41 (1) / Euparal // Præp.: G-15 / H. Enghoff // 11 USSR Azerbai- / dzan. Zakatali / State Res. Agke- / mal 24–27. v.1981 / Golovatch & Martens // № 7 // 00000_65. – *Nopoiulus densepilo*- / *sus* Enghoff PARATYPE / ♀, 1:P2+left vul / va, 2: right vulva / Euparal // Præp.: G-14 / H. Enghoff // 11 USSR Azerbai- / dzan. Zakatali / State Res. Ageke- / mal 24–27. v.1981 / Golovatch & Martens // № 8 // 00000_65.

**Current nomenclature**. *Nopoiulus densepilosus* Enghoff, 1984 (Enghoff 1984), vs *Archinopoiulus densepilosus* (Enghoff, 1984) (Sierwald et al. 2026); the latter combination has never been used but appears in Millibase.

**Remarks**. The remaining paratypes are in ZMF (1♀) and NHMD (1♀).

***extremus* Enghoff, 1984 (*Nopoiulus* )**

*Nopoiulus extremus* Enghoff, 1984: 417, figs 19, 38–50 (D♂♀).

**Types. *Holotype***: (♂), MYR_DIP_0000097. ***Paratypes***: (1♀), MYR_DIP_0000066; (1♀), MYR_DIP_0000095; (1♂), MYR_DIP_0000096; (1♀), MYR_DIP_0000204.

**Type locality**. Azerbaijan, Hirkan (= Hyrcan) Nature Res. (ca 38°28'N, 48°41'E).

**Label data**. MYR_DIP_0000097: *Nopoiulus (Paranopoiulus)* / *extremus* Enghoff / ♂ голотип и паратипы ♂ и ♀ / Опр. {Det.} Enghoff. / Сбор {Collected} Азербайджан, / Ленкорань, Гирканский / зап-к и Астара, leg. Р. Рахманов / и А. Покаржевский {Azerbaijan, Lenkoran, Hyrkan Res. and Astara, leg. R. Rakhmanov and A. Pokarzhevsky} // Ленкорань / лес / № 9. / Подстилка {Lenkoran, forest, litter} / 10.X.1969 // Azerbaidjan, Lenkoran, / Hyrcan forest (Reserve), / 10.X. 1969. leg. R. Rakhmanov // *Nopoiulus* sp. 1 ♂// *Nopoiulus (Paranopoiulus) extremus* n. sp. / ♂ HOLOTYPE / H. Enghoff det., 1982 // Praep G 1–3 / H. Enghoff.— Micropreparations (×3): *Nopoiulus extremus* / Enghoff Holotype / ♂ P1 oral, P2 ab- / oral, left P9 (par- / t) ventral. // Lenkoran, Hyrc- / an forest. 10.x. / 1969, Rakhmanov / Euparal // Præp.: G-1 H. Enghoff // №11 // 00000_97. – *Nopoiulus extre*- / *mus* Enghoff. ♂ HOLOTYPE // Præp.: G-2 / H. Enghoff // P10 d, P11 s; P12 d, P13 s; P14 d, P15 s; P16 s; P17 s; 56 M. / XVII // № 12 // 00000_97. – *Nopoiulus extremus* / Enghoff Holotype / ♂ P9 basis, oral, / right P9 (part) / dorsal // Lenkoran, Hyrcan / forest. 10.x.1969 / Rakhmanov. / Euparal // Præp.: G-3 / H. Enghoff // № 13 // 00000_97.

MYR_DIP_0000066: *Nopoiulus (Paranopoiulus)* / *extremus* Enghoff, / Паратипы ♀ / Опр. {Det.} Enghoff / Сбор {Collected} Азербайджан, / Ленкорань {Azerbaijan, Lenkoran}, 8 и {and} 10.X.1969. / leg. Р. Рахманов {R. Rakhmanov} // *Nopoiulus (Paranopoiulus) extremus* n. sp. / ♀ PARATYPE. / H. Enghoff det., 1982 // præp. G-21–22. H. Enghoff // Azerbajan / Lenkoran / Astara / forest litter / 10.X.1969, leg. R. Rakhmanov.

MYR_DIP_0000204: *Nopoiulus (Paranopoiulus) extremus* n. sp. / ♀ PARATYPE. / H. Enghoff det., 1982. / Ussr Azerbaijan, Lenkoran, Astara / forest litter. 8. X.1969. R. Rakhmanov.

MYR_DIP_0000095: *Nopoiulus (Paranopoiulus) */ *extremus* Enghoff / ♂ голотип и паратипы ♂ и ♀ / Опр. {Det.} Enghoff / Сбор {Collected} Азербайджан, / Ленкорань, Гирканский / зап.-к и Астара, leg. Р. Рахманов / и А. Покаржевский {Azerbaijan, Lenkoran, Hyrkan Res., and Astara, leg. R. Rakhmanov and A. Pokarzhevsky} // Azerbaijan, Lenkoran, / Astara, forest litter / leg. R.R. Rakhmanov / 8.X.1969 // *Nopoiulus (Paranopoiulus) extremus* n. sp. / ♀ PARATYPE / H. Enghoff det., 1982.— Micropreparations: *Nopoiulus extremus* / Enghoff PARATYPE / ♀ P2 oral, vulvae / Euparal // Præp.: G-21 / H. Enghoff // Lenkoran, Astara / 10.x.1969 / Rakhmanov leg // № 14 // 00000_95. – *Nopoiulus extremus* / Enghoff PARATYPE / ♀ right ant. -P30- / left ant. -P31 / Euparal G-22 // Præp.: G-22 / H. Enghoff // Lenkoran, Astara / 10.x.1969, Rakhmanov leg. // № 15 // 00000_95.

MYR_DIP_0000096: *Nopoiulus (Paranopoiulus)* / *extremus* Enghoff / ♂ голотип и паратипы ♂ и ♀ / Опр. {Det.} Enghoff. / Сбор {Collected} Азербайджан, / Ленкорань, Гирканский / зап-к и Астара, leg. Р. Рахманов / и А. Покаржевский {Azerbaijan, Lenkoran, Hyrkan Res., and Astara, leg. R. Rakhmanov and A. Pokarzhevsky} // Caucasus / Lenkoran / Talysh Mts., / The Hyrcan Reserv. / oak wood litter / 30.I.1975 / leg. A. Pokarzhevsky // 30/I-75 г. Талыш / Гирк. з.-к. / Почв. проба № 2, дубовый / лес. влажный / лист {Talysh, Hyrkan Res., soil sample № 2, oak forest, wet leaves} // *Nopoiulus* (Paranopoiulus) extremus n. sp. / ♂ PARATYPE. / H. Enghoff det., 1982 // Praep G 20 / H. Enghoff .— Micropreparation: *Nopoiulus extremus* / Enghoff, PARATYPE / ♂ right ant.-P30- / clypeus-left P31- / gnathochil. Euparal // Lenkoran: Talysh / Mts. 30.i.1975. / Pokarzhewsky leg. // Præp.: G-20 / H. Enghoff // № 16 // 00000_96.

**Current nomenclature**. *Nopoiulus extremus* Enghoff, 1984 (Sierwald et al. 2026).

**Remarks**. The remaining paratypes are in ZMF (1♂) and NHMD (1♂ 1♀). The holotype and paratype samples MYR_DIP_0000095–96 dried out and were moistened in lactic acid on 2.V.2023.

***golovatchi* Enghoff, 1984 (*Nopoiulus* )**

*Nopoiulus golovatchi* Enghoff, 1984: 420, figs 19, 51, 53, 55–60 (D♂♀).

**Types. *Holotype***: (♂), MYR_DIP_0000067. ***Paratypes***: (2 ♀), MYR_DIP_0000068.

**Type locality**. Abkhasia, Sukhumi Botanical Garden (ca 43°00'N, 41°01'E).

**Label data**. MYR_DIP_0000067: *Nopoiulus (Paranopoiulus)* / *golovatchi* Enghoff / Опр. {Det.} Enghoff / Сбор {Collected} Абхазия, Сухумский / бот. сад, подстилка {Abkhasia, Sukhumi botanical garden, litter}, 9. X.1978 / С.И. Головач leg. {S.I. Golovatch} // *Nopoiulus (Paranopoiulus) golovatchi* n.sp. / ♂ HOLOTYPE. / H. Enghoff det., 1982 // præp. G-4, G-17. / H. Enghoff // right P9 in tube! // Abkhasia / Sukhumi / Botanical Garden / litter, 9.X.1978. Leg. Golovatch.— Micropreparations (×2): *Nopoiulus golo*- / *vatchi* / Enghoff / Holotype / ♂ left ant., P 1 / oral, P2 aboral / left P9 lateral // Præp.: G-4 / H. Enghoff // Abchasia: Sukhu- / mi, bot. gard., 9.x. / 1978, Golovatch / G-4 Euparal // № 17 // 00000_67 – *Nopoiulus golovatchi* / Enghoff Holotype / ♂ right ant., cly- / peus, left md., / P28, gnathochil. / P29, Euparal / No 18 // Abchasia: Sukhumi / bot. gard. 9.x. / 1978. Golovatch // Præp.: G-17 / H. Enghoff // 00000_67.

MYR_DIP_0000068: *Nopoiulus (Paranopoiulus)* / *golovatchi* Enghoff / Опр. {Det.} Enghoff / Сбор {Collected} Абхазия, Сухумский / бот. сад, подстилка. 9. X.1978. / С.И. Головач leg. // MYR_DIP_0000068 // *Nopoilus (Paranopoiulus) golovatchi* n. sp. / 2 ♀ PARATYPES / H. Enghoff det., 1982 / Ussr, Abkhasia, Sukhumi, Botanical Garden, / litter, 9.X.1978. S. Golovatch leg // præp. G-18. H. Enghoff.— Micropreparations (×2): *Nopoiulus golovatchi* / Enghoff, PARATYPE / ♀ P2 aboral, / right (?) vulva / Euparal // Abchasia: Sukhumi / bot. gard., 9.x. / 1978, Golovatch // Præp.: G-18 / H. Enghoff // 00000_68. – *Nopoiulus golovatchi* / Enghoff, PARATYPE / ♀ P28-right ant. / P29, Euparal // Præp.: G-19 / H. Enghoff // Abchasia: Sukhumi / bot. gard., 9.x.1978 / Golovatch // № 20 // 00000_68.

**Current nomenclature**. *Nopoiulus golovatchi* Enghoff, 1984 (Sierwald et al. 2026).

**Remarks**. The remaining paratypes are in ZMF (1♀) and NHMD (1♂ 1♀).

***iranicus* Enghoff, 1984 (*Nopoiulus* )**

*Nopoiulus iranicus* Enghoff, 1984: 422, 19, 61 (D♀).

**Type. *Paratype***: (1♀), MYR_DIP_0000080.

**Type locality**. Iran, Mazandaran Prov., 12 km NW of Zirab (ca 36°11'N, 52°58'E).

**Label data**. *Nopoiulus (Paranopoiulus) iranicus* Enghoff / Опр. {Det.} Enghoff / Сбор {Collected} Iran, Prov. Masandaran / 11 km E of Alasht, 1400 m dense / forest, wet, 27.VI.1978. leg. / J. Martens & H. Pieper / паратип ♀ // Blaniulidae / Iran, Prov. Masandaran, 11 km E of Alasht / 1400 m, dense forest, wet. / 27.VI.1978. JM [J. Martens] + HP [H. Pieper] 18 // præp. M-5–6 H. Enghoff // *Nopoiulus (Paranopoiulus) iranicus *n. sp. / ♀ PARATYPE / H. Enghoff det., 1982.— Micropreparations (×2): *Nopoiulus iranicus* / Enghoff, PARATYPE / ♀ P2+vulvae (left / vulva free from / membranes), Eupa- / ral // Iran 18:1 km E / Alasht. 27. VI.1978 / Martens & Pieper // Præp.: M-5 / H. Enghoff. – *Nopoiulus iranicus* / Enghoff, PARATYPE / ♀ clypeus, right / ant.-P30-gnatho- / chil.-P31 // Iran 18:1 km E / Alasht, 27.vi. / 1978. Martens & / Pieper / № 29 // Præp.: M-6 / H. Enghoff.

**Current nomenclature**. *Nopoiulus iranicus* Enghoff, 1984 (Sierwald et al. 2026).

**Remarks**. The holotype ♀ and 1 juv. ♂ are in ZMF, the remaining paratype (1♀) is in NHMD.

***pensylvanicus* Brandt, 1840 (*Julus* )**

*Julus pensylvanicus* Brandt, 1840c: 102 (D♂♀).

**Type. *Syntype***: (1♀), MYR_DIP_0000295.

**Type locality**. USA, Pennsylvania (no exact locality).

**Label data**. Lectotype / design. S. Golovatch // *Julus pensylva*- / *nicus* Nob. / Pensylv. Mus. Ber. Zimmerman.

**Current nomenclature**. *Ptyoiulus impressus* (Say, 1821) (Sierwald et al. 2026). Synonymised with *P. impressus* by Hoffman (1999).

**Remarks**. The syntype is dried and pinned, its body is broken into two pieces.

### Family Julidae

***abchasica* Lohmander, 1936 (*Archileucogeorgia* )**

*Archileucogeorgia abchasica* Lohmander, 1936: 91, figs 71–74, 77, 78 (D♂).

**Type. *Holotype***: (♂), MYR_DIP_0000263.

**Type locality**. Abkhazia, nr Sukhumi, Kelasuri (= Aleksandrovskaya) Cave (ca 43°01'N, 41°08'E).

**Label data**. Зоол. Муз. АН {Zoological Museum of the Academy of Sciences} / №253-1910 г. / 805. *Archileucogeorgia* / *abchasica* Lohm. / Опр. {Det.} Type / Сбор {Collected} 19.IX.1909 г. Сухум- / ский окр. 38 Кела- / сурская пещера {Sukhumi Province, 38 Kelasurskaya cave} / К. Сатунин {K. Satunin} // *Archileucogeorgia* / *abchasica* n.sp. / Type Hans Lohmander.

**Current nomenclature**. *Leucogeorgia abchasica* (Lohmander, 1936) (Sierwald et al. 2026). Transferred to the genus *Leucogeorgia* Verhoeff, 1930 by Antić and Reip (2020).

**Remarks**. The collection date, given by Lohmander (1936: 95) as 2.X.1910, does not correspond to that indicated on the label. The holotype is fragmented and its body pieces are shared between two sample tubes, with the body in one and small fragments of its head-section in the other.

***adsharicus* Lohmander, 1936 (*Amblyiulus* )**

*Amblyiulus adsharicus* Lohmander, 1936: 156, figs 131–132 (D♂♀).

**Types. *Syntypes***: (4♂ 1♀), MYR_DIP_0000236.

**Type locality**. Turkey, Artvin Prov., Borçka (ca 41°22'N, 41°41'E).

**Label data**. Зоол. Муз. АН {Zoological Museum of the Academy of Sciences} / №17-1911 г. / 801. *Amblyiulus* / *adsharicus* Lohm. / Опр. {Det.} Typen / Сбор {Collected} 15.V.1910 г. / Батумская обл. {Batumi Oblast} / Порчха {Porchkha} / П. Нестеров {P. Nesterov [col.]}.

**Current nomenclature**. *Syrioiulus adsharicus* (Lohmander, 1936) (Vagalinski 2020) vs *Heteropachyiulus adsharicus* (Lohmander, 1936) (Sierwald et al. 2026). Transferred to *Syrioiulus* Verhoeff, 1914 by Vagalinski (2020); see also Evsyukov et al. (2021).

**Remarks**. All type specimens are fragmented and shared between two sample tubes, with their fragmented bodies and one head in one, and four heads and some body fragments in the other.

***adsharicus* Lohmander, 1936 (*Chromatoiulus* )**

*Chromatoiulus adsharicus* Lohmander, 1936: 123, figs 99–101 (D♂♀).

**Types. *Syntypes***: (2♂ 1♀), MYR_DIP_0000304.

**Type locality**. Georgia, Adjaria, Acharistskali R. (coordinates are given for Khelvachauri, Batumi; ca 41°34'N, 41°40'E).

**Label data**. *Chromatoiulus* / *adsharicus* n.sp. / Typen / Hans Lohmander. // Зоол. Муз. АН {Zoological Museum of the Academy of Sciences} / №138-1915 г. / 772. *Chromatoiulus* / *adsharicus* Lohm. / Опр. {Det.} Typen / Сбор {Collected} 21.VIII.1914 г. Батум- / ская обл. Устье реки / Аджария-Цхали. {Batumi Oblast, valley of Acharistskali R.} / К.В. Суходольский {K.V. Sukhodolsky [col.]}.

**Current nomenclature**. *Omobrachyiulus adsharicus* (Lohmander, 1936) (Sierwald et al. 2026). Transferred to *Omobrachyiulus* Lohmander, 1936 by Vagalinski and Lazányi (2018).

**Remarks**. The remaining syntype (1♀) is now kept in GMNH (MYRIA000014645; O. Gran, pers. comm., 5.II.2026). The ZISP syntypes dried out and were moistened on 13.IV.2026; all of them have their head sections detached from the rest of their bodies.

***altaicus* Gulička, 1972 (*Cylindroiulus*)**

*Cylindroiulus altaicus* Gulička, 1972: 43, fig. 5.2 (D♂).

**Type. *Holotype***: (♂), MYR_DIP_0000062.

**Type locality**. Russia, Altai, Kosh-Agatch Distr., nr Chagan-Uzun (ca 50°06'N, 88°22'E); lake.

**Label data**. *Cylindroiulus* / *altaicus* Gulička / Опр. {Det.} Holotypus ♂ / Сбор {Collected} А.М. Гилярова {A.M. Ghilyarov} / Юго-Вост. Алтай {SE Altai}/ оз. Аю-Коль {Ayu-Kol’ Lake} ~ 800 / м.н.у.м. {m a.s.l.} 17.VIII.962 // *Cylindroiulus altaicus* n.sp. / Altaj. kraj – Holotypus // Юго-Восточный Алтай {SE Altai} / у оз. Аю-Коль {nr Ayu-Kol’ Lake}, ~ 800 м. у.м., {m a.s.l.} / пихтовый лес {spruce forest}, 17.VIII.1962 / ♂ голотип. / leg. А.М. Гиляров {A.M. Ghilyarov}.

**Current nomenclature**. *Sibiriulus altaicus* (Gulička, 1972) (Mikhaljova 2017; Sierwald et al. 2026). Listed and indirectly transferred to *Sibiriulus* Gulička, 1972 by Lokšina and Golovatch (1979), at present firmly regarded as a species of *Sibiriulus* (Mikhaljova 2017).

**Remarks**. This species was described from the holotype ♂ alone, which is presently broken in two parts. Mikhaljova et al. (2014: 45) mistakenly assumed that the holotype ♂ of this species had been lost, which is not the case. The holotype was returned after a scandal, deposited by SIG in ZISP.

***anguiglossus* Lohmander, 1932 (*Leptoiulus* )**

*Leptoiulus anguiglossus* Lohmander, 1932a: 12, fig. 10 (D♀).

**Type. *Holotype***: (♀), MYR_DIP_0000271.

**Type locality**. Iran, Astrabad (= Gorgan) (ca 36°14'N, 46°25'E).

**Label data**. Зоол. Муз. АН {Zoological Museum of the Academy of Sciences} / №527-15 г. / 591. *Leptoiulus* / *anguiglossus* Lohm. / Опр. {Det.} spec. orig.! / Сбор {Collected} Persien, Astra- / bad, 2.V.1914 г. / A. Kiritschenko // Persien: / Astrabad / 2.V.1914 / A. Kiritschenko // *Leptoiulus* / *anguiglossus* n.sp. / Hans Lohmander.

**Current nomenclature**. *Leptoiulus anguiglossus* Lohmander, 1932 (Sierwald et al. 2026).

**Remarks**. The holotype is devoid of the head and some front segments.

***arabistanus* Lohmander, 1932 (*Leptoiulus* )**

*Leptoiulus arabistanus* Lohmander, 1932a: 14, figs 11–12, 17, 20 (D♂♀).

**Types. *Syntypes***: (2♂ 2♀ + 2 additional specimens), MYR_DIP_0000309. ***Other material***: (2♂ 7♀), MYR_DIP_0000240.

**Type locality**. Iran, Khuzestan Prov., Cheshme-Rogan spring nr Dehdez (ca 31°43'N, 50°17'E).

**Label data**. MYR_DIP_0000309: *Leptoiulus* / *arabistanus* n.sp. / Hans Lohmander. // Зоол. Муз. АН {Zoological Museum of the Academy of Sciences} / №97-1904 г. / 597. *Leptoiulus* / *arabistanus* Lohm. / Опр. {Det.} spec. orig.! / Сбор {Collected} Зап. Персия, Ара- / бистан, эксп. в окр. сел. Дех- / и-Диз (22 в.), {West Persia, Arabistan, expedition in the vicinity of Dekh-i-Diz (22 century)} 25.XII.1903 г. / Н. Зарудный {N. Zarudnyi [col.]}.

MYR_DIP_0000240: *Amblyiulus* / *arabistanus* n.sp. / Hans Lohmander. // Зоол. Муз. АН {Zoological Museum of the Academy of Sciences} / №97-1904 г. / 596. *Amblyiulus* / *arabistanus* Lohm. / Опр. {Det.} spec. orig.! / Сбор {Collected} Зап. Персия, Арабис- / тан, род. Чешме-Ро- / ган (24–25 в.) {West Persia, Arabistan, Cheshme-Rogan spring (23–24 centuries)} 31.XII.1903 г. / Н. Зарудный {N. Zarudnyi [col.]}.

**Current nomenclature**. Leptoiulus (Iraniulus) arabistanus Lohmander, 1932 (Sierwald et al. 2026).

**Remarks**. The collection date of the sample MYR_DIP_0000309 as given by Lohmander (1932a: 16), 7.X.1904, fails to correspond to the date indicated on the label. Yet, the remaining data clearly correspond to those from the original description and hence the specimens indeed represent the syntypes. Additionally, the GMNH collection holds one spirit-preserved specimen of an unspecified sex (apparently ♂) that was collected by N Zarudny from “Persien, Arabistan, Dorf Deck-i-Diz” on 7.I.1904 (MYRIA000014748; O. Gran, pers. comm., 29.I.2026). Its label data are identical to those given by Lohmander (1932a: 16), and hence it should be a syntype. The sample MYR_DIP_0000309 dried out, and was moistened on 14.IV.2026. It contains two broken specimens that cannot be sexed, as their anterior body sections are missing. However, according to Lohmander (1932a: 14), the original type series contained 5♂ and 2♀, and hence the broken torsos are likely to belong to males.

In the sample MYR_DIP_0000240, except for the country and province, the rest of the original label’s content (viz., locality, date, and specimens’ number) fails to correspond to the information given by Lohmander (1932a). One male and one female are headless.

***astrabadensis* Lohmander, 1932 (*Amblyiulus* )**

*Amblyiulus astrabadensis*Lohmander 1932a: 30, figs 30–32 (D♂♀).

**Types. *Syntypes***: (1♂ 2♀), MYR_DIP_0000241.

**Type locality**. Iran, Astrabad (= Gorgan) (ca 36°14'N, 46°25'E).

**Label data**. Зоол. Муз. АН {Zoological Museum of the Academy of Sciences} / №81-05 г. / 595. *Amblyiulus* / *astrabadensis* Lohm. / Опр. {Det.} spec. orig.! / Сбор {Collected} Персия, Астра- / бад. {West Persia, Astrabad} 19.IV.1905 г. / Е. Филиппович {E. Filippovich [col.]} // *Amblyiulus* / *astrabadensis* n.sp. / Hans Lohmander.

**Current nomenclature**. *Syrioiulus astrabadensis* (Lohmander, 1932), first transferred to *Syrioiulus* Verhoeff, 1914 by Mauriès (1984); see also Enghoff and Moravvej (2005).

**Remark**. The original syntype series consisted of 5♂ and 5♀ (Lohmander 1932a: 30), of which three syntypes are now in ZISP (see above), and two additional syntypes (1♂ 1♀) and four micropreparations are in GMNH (MYRIA000014623; O. Gran, pers. comm., 5.II.2026); revised by Vagalinski (2020). The whereabouts of the remaining syntypes (3♂ 2♀) is unknown. The collection date given by Lohmander (1932a: 32) as 2.V.1905 does not correspond to the date indicated on the ZISP label (19.IV.1905), but corresponds to that of the syntypes housed in GMNH (see Vagalinski 2020: 88).

***astrabadensis* Lohmander, 1932 (*Leptoiulus* )**

*Leptoiulus astrabadensis* Lohmander, 1932a: 10, figs 7–9, 14, 15, 18 (D♂♀).

**Types. *Syntypes***: (1♂ 2♀), MYR_DIP_0000258. – ***Other specimens***: (2♂ 6♀), MYR_DIP_0000241; (1♂ 1 juv.), MYR_DIP_0000257; (2♂ 1♀), MYR_DIP_0000259.

**Type locality**. Iran, Astrabad (= Gorgan) (ca 36°14'N, 46°25'E).

**Label data**. MYR_DIP_0000258: Зоол. Муз. АН {Zoological Museum of the Academy of Sciences} / №527-15 г. / 593. *Leptoiulus* / *astrabadensis* Lohm. / Опр. {Det.} spec. orig.! / Сбор {Collected} Персия, Астра- / бад, {Persia, Astrabad} 20.IV.1914 г. / А. Кириченко {A. Kirichenko} // Persien: / Astrabad / 3.V.1914 / A. Kiritschenko // *Leptoiulus* / *astrabadensis* n.sp. / Hans Lohmander.

MYR_DIP_0000257: Зоол. Муз. АН {Zoological Museum of the Academy of Sciences} / №527-15 г. / 595. *Leptoiulus* / *astrabadensis* Lohm. / Опр. {Det.} spec. orig.! / Сбор {Collected} Персия, Астра- / бад, {Persia, Astrabad} 14.IV.1914 г. / А. Кириченко {A. Kirichenko} // Persien / Astrabad / 27.IV.1914 / A. Kiritschenko // *Leptoiulus* / *astrabadensis* n.sp. / Hans Lohmander.

MYR_DIP_0000259: Зоол. Муз. АН {Zoological Museum of the Academy of Sciences} / №527-15 г. / 592. *Leptoiulus* / *astrabadensis* Lohm. / Опр. {Det.} spec. orig.! / Сбор {Collected} Персия, Астра- / бад, {Persia, Astrabad} 14.IV.1914 г. / А. Кириченко {A. Kirichenko} // Persien: / Astrabad / 27.IV.1914 / A. Kiritschenko // *Leptoiulus* / *astrabadensis* n.sp. / Hans Lohmander.

**Current nomenclature**. *Leptoiulus astrabadensis* Lohmander, 1932 (Enghoff and Moravvej 2005; Golovatch et al. 2022a).

**Remarks**. Based on Lohmander (1932a: 11), the original type series of *L. astrabadensis* (2♂ 2♀) was collected from Astrabad (= Gorgan) by A. Kirichenko on 3 May 1914. There is only one sample in the ZISP collection (MYR_DIP_0000258) that was taken on that day, but it contains 1♂ and 2♀. For this reason, we consider these specimens to be true syntypes, while the remaining specimens are to be treated as ‘Other material’, even though all are topotypes. Each aforementioned sample contains some specimens that are fragmented into two or more pieces. Two samples, MYR_DIP_0000258 and MYR_DIP_0000259, contain contradictory collection dates on the labels enclosed: 20.IV vs 3.V and 14.IV vs 27.IV, respectively. However, two more syntypes are kept in GMNH (O. Gran, pers. comm., 26.I.2026): two torsos of unspecified sex (apparently, 1♂ 1♀), and four micropreparations (two of the male, and two of the female).

***bicolor* Lohmander, 1932 (*Cylindroiulus* )**

*Cylindroiulus bicolor* Lohmander, 1932b: 172, figs 4, 5 (D♂♀).

**Types. *Syntypes***: (2♀), MYR_DIP_0000255; (5♂ 1♀), MYR_DIP_0000256.

**Type locality**. Iran, Ardabil Prov., E of Sang Abad, Bagrov-Dag Mt. (ca 37°45'N, 48°21'E).

**Label data**. MYR_DIP_0000255: Зоол. Муз. АН {Zoological Museum of the Academy of Sciences} / №454-14 г. / 583. *Cylindroiulus* / *bicolor* Lohm. / Опр. {Det.} spec. orig. / Сбор {Collected} Сев. Персия, {North Persia} / Багров-Даг, вост. скл. {Bagrov-Dag [Mt.], east slope} / 7.VI.1914 г. / С. Фон-Вик {S. von Wick} // *Cylindroiulus* / *bicolor* n.sp. / Hans Lohmander.

MYR_DIP_0000256: Зоол. Муз. АН {Zoological Museum of the Academy of Sciences} / №306-15 г. / 582. *Cylindroiulus* / *bicolor* Lohman. / Опр. {Det.} spec. orig. / Сбор {Collected} Сев. Персия, вост. / скл. Багров-Дага, коч. / Алчамых, {North Persia, east slope of Bagrov-Dag [Mt.], nomadic stand Alchamykh} 5.VII.1914 г. / С. Фон-Вик {S. von Wick} // *Cylindroiulus* / *bicolor* n.sp. / Hans Lohmander.

**Current nomenclature**. *Cylindroiulus bicolor* Lohmander, 1932 (Sierwald et al. 2026).

**Remarks**. The collection date as given by Lohmander (1932b: 174), 18–20.VII.1914, does not correspond to the dates indicated on the labels. In the sample MYR_DIP_0000256, three males are devoid of their front parts. One female in the sample MYR_DIP_0000255 is fragmented. Two more syntypes (1♂ and one of an unspecified sex) are kept in GMNH: two torsos and seven micropreparations (MYRIA000014662–65; O. Gran, pers. comm., 26.I.2026).

***brachymeritus* Enghoff, 1987 (*Nepalmatoiulus* )**

*Nepalmatoiulus brachymeritus* Enghoff, 1987: 328, figs 47, 53, 217, 218 (D♂♀).

**Types. *Holotype***: (♂), MYR_DIP_0000073. ***Paratypes***: (2♀), MYR_DIP_0000074.

**Type locality**. China, Sichuan, Kangding (= Da-tsien-lu or Tachienlu) (ca 30°03'N, 101°53'E).

**Label data**. MYR_DIP_0000073: Зоол. Инст. АН {Zoological Institute of the Academy of Sciences} / голотип ♂ паратипы ♀ / *Nepalmatoiulus brachymeritus* / Enghoff, 1987 / *Nepalmatoiulus brachymeritus* Enghoff, 1987. / Место {Site} China, Szechuan, Da-tsien- / lu, 17. VI.1893 / Колл. leg. N. Potanin // *Nepalmatoiulus brachymeritus* Enghoff / ♂ HOLOTYPE // China, Szechuan Prov., / Da-Tsien-lu. 17.VI.1893. / leg. G.N. POTANIN. / Zool. Mus. Leningrad // Тацзинлу {Da-tzien-lu} / (Центр. Китай) {Central China}/ Потанинь {Potanin} / 17-VI-1893 // Potanin 17.VI.1893. / Da-Tsien-lu // Cat. № 17. Spm. 1// 796 ♂ //, præp. G-59. H. Enghoff //♂ и G-59.— Micropreparation: *Nepalmatoiulus brachymeritus* Enghoff ♂. HOLOTYPE. Szechuan, Da-tsien-lu. Potanin // Gnathochil., P48, midbody dorsal limbus / left ant., P49. Euparal. Præp. G-59. H. Enghoff // 00000_73.

MYR_DIP_0000074: Micropreparation: *Nepalmatoiulus brachymeritus* Enghoff ♀. PARATYPE. Szechuan: Da-tsien-lu. Potanin. Præp. G-60. H. Enghoff // right vulva. Faure-Berlese/ Gold Size // 00000_74. – *Nepalmatoiulus brachymeritus* Enghoff ♀. PARATYPE. Szechuan: Da-tsien-lu. Potanin. Præp. G-61. H. Enghoff // gnathochil. right ant., P48/midbody dorsal limbus, left ant., right P49. Euparal // 00000_74.

**Current nomenclature**. *Nepalmatoiulus brachymeritus* Enghoff, 1987 (Sierwald et al. 2026).

**Remarks**. Based on the original description (Enghoff 1987: 328–329), there were eight paratype ♀♀ studied by the author. Of them, only two are currently available in the ZISP collection; the whereabouts of the other paratypes remain unknown, but are likely in NHMD. The torsos of the two paratype females are absent from the jar, although their micropreparations are available in the collection.

***brachyurus dagestanus* Lohmander, 1936 (*Chromatoiulus* )**

*Chromatoiulus brachyurus dagestanus* Lohmander, 1936: 121, figs 96–98 (D♂).

**Types. *Syntypes***: (2♀), MYR_DIP_0000224; (1♂), MYR_DIP_0000225.

**Type locality**. Russia, Republic of Dagestan: Kamilukh Vil. (ca 41°55'N, 46°38'E), or Kumukh Vil. (ca 42°10'N, 47°08'E). Since no lectotype has been designated, the type locality cannot be unambiguously selected.

**Label data**. MYR_DIP_0000224: Зоол. Муз. АН {Zoological Museum of the Academy of Sciences} / №398-1909 г. / 769. *Chromatoiulus* / *brachyurus dagestanus* / n. subsp. / Опр. {Det.} H. Lohmander / Сбор {Collected} 3.VIII.1909 г. Дагестан {Daghestan} / сел. Камелюк, Гу- {Kamelyuk Vil., Gu-} / нибский окр. {nibsky Distr.} / А. Млокосевич {A. Mlokosevitch} [col.] // *Chrom. brachyurus* / *dagestanus* n.subsp. / Hans Lohmander.

MYR_DIP_0000225: Зоол. Муз. АН {Zoological Museum of the Academy of Sciences} / №398-1909 г. / 770. *Chromatoiulus* / *brachyurus dagestanus* / n. subsp. / Опр. {Det.} H. Lohmander / Сбор {Collected} 14.VII.1909 г. Дагестан {Daghestan} / г. Кумух; в луже {Kumukh Mt.; in puddle} / А. Млокосевич {A. Mlokosevitch} [col.] // *Chrom. brachyurus* / *dagestanus* n.subsp. / Hans Lohmander.

**Current nomenclature**. *Omobrachyiulus caucasicus* (Karsch, 1881) (Vagalinski and Golovatch 2021). The taxonomic status of *Omobrachyiulus* Lohmander, 1936 was revised by Vagalinski and Lazányi (2018: 92, 94), who listed *C. brachyurus dagestanus* Lohmander, 1936 as a synonym of *O. brachyurus*, while Vagalinski and Golovatch (2021: 35) formally synonymised *O. brachyurus* under *O. caucasicus* (Karsch, 1881).

**Remarks**. The collection dates given by Lohmander (1936: 123) as 16.VIII.1909 for MYR_DIP_0000224 and 27.VIII.1909 for MYR_DIP_0000225 fail to correspond to those indicated on the labels. The syntype ♂ in MYR_DIP_0000225 is broken.

***colchicus* Lohmander, 1936 (*Iulus* )**

*Iulus colchicus* Lohmander, 1936: 77, figs 60–68 (D♂).

**Types. *Syntypes***: (1♂ 4 juv.), MYR_DIP_0000232; (1♂), MYR_DIP_0000233.

**Type locality**. Georgia: Gagra (ca 43°17'N, 40°16'E) or Tsikhisdziri (ca 41°46'N, 41°46'E). Since the lectotype has not been designated, the type locality cannot be unambiguously selected.

**Label data**. MYR_DIP_0000233: Зоол. Муз. АН {Zoological Museum of the Academy of Sciences} / №64-1917 г. / 792. *Julus* / *colchicus* Lohm. / Опр. {Det.} Type. / Сбор {Collected} 26.V.1910 г. / Зап. Закавказье {West Transcaucasia}, / Гагры {Gagry}. / В. Линдгольм {V. Lindholm [col.]} // *Julus colchicus* / n.sp. / Type Hans Lohmander.

MYR_DIP_0000233: Зоол. Муз. АН {Zoological Museum of the Academy of Sciences} / №11-1928 г. / 793. *Julus* / *colchicus* n.sp. / Опр. {Det.} L. Lohmander. / Сбор {Collected} 22.IX.1914 г. / Батумская обл. {Batumi Oblast} / Цихис-Дзири {Tsikhis-Dziri}. / А. Быков {A. Bykov [col.]} // *Julus colchicus* / n.sp. / Hans Lohmander.

**Current nomenclature**. *Iulus colchicus* Lohmander, 1936 (Evsyukov et al. 2018), vs *Euxinoiulus colchicus* (Lohmander, 1936) (Sierwald et al. 2026).

**Remarks**. The collection date of the sample MYR_DIP_0000233 as given by Lohmander (1936: 81), 15.X.1914, fails to correspond to the date indicated on the label. Additionally, the sample from Gagra (MYR_DIP_0000232) also contains four juveniles, which were not mentioned by Lohmander and thus cannot be regarded as types. The specimen in the sample MYR_DIP_0000233 is fragmented.

***dentiger* Gulička, 1963 (*Cylindroiulus* )**

*Cylindroiulus dentiger* Gulička, 1963: 519, figs 1.1–3 (D♂♀).

**Types. *Holotype***: (♂), MYR_DIP_0000056.

**Type locality**. Russia, Kemerovo Oblast, Prokopyevsk (ca 53°52'N, 86°38'E).

**Label data**. MYR_DIP_0000056: *Cylindroiulus dentiger* / Gulička / Опр. {Det.} Holotypus ♂ / Сбор {Collected} Ю.Б. Бызовой и З.В. / Чадаевой {Yu.B. Byzova and Z.B. Chadaeva} / Кемеров. обл., Прокопьевск {Kemerovo Oblast, Prokopyevsk} / гидроузел, лес {waterworks, forest} IV–V.60.

**Current nomenclature**. *Sibiriulus profugus* (Stuxberg, 1876) (Mikhaljova 2017; Sierwald et al. 2026). Listed and indirectly transferred to *Sibiriulus*Gulička 1972 by Lokšina and Golovatch (1979), formally synonymised under *S. profugus* by Mikhaljova (2002), at present firmly regarded as a species of *Sibiriulus* Gulička, 1972 (Mikhaljova 2017).

**Remark**. The paratypes (4♂ 5♀) are kept embezzled in the SMNPS collection. No type specimens of this species are available in ZMMU (A. Schileyko, pers. comm., 4.II.2026).

***dioscoriadis* Lignau, 1915 (*Brachyiulus* )**

*Brachyiulus dioscoriadis* Lignau, 1915: 382–387, text figs 12–20, tab. IV: figs 4, 5 (D♂♀).

**Types. *Syntypes***: (1♂ 2♀ 5 juv.), MYR_DIP_0000314; (8♂ 14♀ 4 juv.), MYR_DIP_0000315.

**Type locality**. Abkhasia, Basla River nr Sukhumi (ca 43°01'N, 41°03'E).

**Label data**. MYR_DIP_0000314: *Brachyiulus dioscoriadis* / Lignau / Lignau intern. {illegible word} // 1♂ 2♀ 5 juv // // Зоол. Муз. АН {Zoological Museum of the Academy of Sciences} / №162-05 г. / 66. *Brachyiulus* / *dioscoriadis* Lign. / Опр. {Det.} Н. Лигнау {N. Lignau} / Сбор {Collected} / Сухум. окр. бл. с. / слияния рр. Маруха / и Адынге, в. 1400 м. {Sukhumi Distr., nr confluence of Marukha and Adynge rivers at 1400 m a.s.l.} / 7.VIII.1905 г. / М. Калишевский {M. Kalishevsky}.

MYR_DIP_0000315: *Brachyiulus dioscoriadis* / Lignau / Lignau intern. {illegible word} // 8♂ 14♀ 4 juv // 4 экз. взято для / Д-ра Schubart {4 specimens taken for Dr Schubart} // Сухумский округъ / Венецианский мостъ / на р. Бесла в 5 верстахъ / от г. Сухума {Sukhumi Distr., Venetian Bridge, 5 verst of Sukhumi} / 25.VIII.1905 экс. Калишевского {Kalishevsky’s expedition} // Зоол. Муз. АН {Zoological Museum of the Academy of Sciences} / №162-05 г. / 67. *Brachyiulus* / *dioscoriadis* Lign. / Опр. {Det.} Н. Лигнау {N. Lignau} / Сбор {Collected} Сухумский окр., / венец. мост на р. Бес- / ла в 5 в. от г. Сухума {Sukhumi Distr., Venetian Bridge, 5 verst of Sukhumi} / 25.VIII.1905 г. / М. Калишевский {M. Kalishevsky}.

**Current nomenclature**. Megaphyllum (Colchiobrachyiulus) dioscoriadis (Lignau, 1915) (Sierwald et al. 2026). Transferred to *Colchiobrachyiulus* Lohmander, 1936 by Vagalinski and Golovatch (2021), who also assumed that syntypes of the sample from Marukha River in the Karachay-Cherkess Republic (MYR_DIP_0000314) may actually belong to *Colchiobrachyiulus montanus* Vagalinski, 2021.

**Remarks**. Many syntypes are broken in two or more parts.

***discolor* Lohmander, 1932 (*Amblyiulus* )**

*Amblyiulus discolor* Lohmander, 1936: 28, figs 27–29 (D♂♀).

**Types. *Syntypes***: (2♂ 1♀), MYR_DIP_0000239.

**Type locality**. Iran, Astrabad (= Gorgan) (ca 36°14'N, 46°25'E).

**Label data**. Зоол. Муз. АН {Zoological Museum of the Academy of Sciences} / №97-1904 г. / 526. *Amblyiulus* / *discolor* Lohm. / Опр. {Det.} spec. orig.! / Сбор {Collected} Персия, Астрабад, {Persia, Astrabad} / М. Гермаб-Даши {M. Germab-Dashi} / 7–9.X.1903 г. / Н. Зарудный {N. Zarudnyi [col.]} // Persien: / Astrabad / Germab-daschi / 20–22.X.1903 / [on opposite side of the label]: №97-901 / N. Zarudny // *Amblyiulus* / *discolor* n.sp. / Hans Lohmander.

**Current nomenclature**. *Syrioiulus discolor* (Lohmander, 1932) (Sierwald et al. 2026). Transferred to *Syrioiulus* by Mauriès (1984).

**Remark**. Both males are broken.

***disparatus* Lohmander, 1936 (*Leptoiulus* )**

*Leptoiulus disparatus* Lohmander, 1936: 60, figs 34–37 (D♂).

**Types. *Holotype***: (♂), MYR_DIP_0000305.

**Type locality**. Georgia, Borjomi (ca 41°50'N, 43°23'E).

**Label data**. *Leptoiulus* / *disparatus* n.sp. / Type Hans Lohmander. // Зоол. Муз. АН. {Zool[ogical] Mus[eum] A[cademy] of S[ciences]} / №373-1912 г. / 802. *Leptoiulus* / *disparatus* Lohm. / Опр. {Det.} Type / Сбор. {Col.} 10.VI.1912 г. / Тифлисс. губ. Окр. / Боржома. {Tiflis Province, nr. Borjomi} / Л. Млокосевич. {L. Mlokosevitch [col.]}.

**Current nomenclature**. *Leptoiulus disparatus* Lohmander, 1936 (Sierwald et al. 2026).

**Remarks**. The holotype is fragmented in six body parts.

***divaricatus* Lohmander, 1936 (*Chromatoiulus* )**

*Chromatoiulus divaricatus* Lohmander, 1936: 135, figs 115–119 (D♂♀).

**Types. *Syntypes***: (2♂ 4♀), MYR_DIP_0000214; (2♂), MYR_DIP_0000215; (2♂), MYR_DIP_0000267. – ***Other material***: (2♀), MYR_DIP_0000216.

**Type locality**. Armenia, Dilijan (ca 40°44'N, 44°52'E), or Georgia, Borjomi (ca 41°50'N, 43°23'E); the locality ‘Arsiansky fast’ in Adjara (Georgia) is not found. Since the lectotype has not been designated, the type locality cannot be unambiguously selected.

**Label data**. MYR_DIP_0000214: *Chromatoiulus* / *divaricatus* / Typen Hans Lohmander // Зоол. Муз. АН. {Zool[ogical] Mus[eum] A[cademy] of S[ciences]} / №617-1927 г. / 773. *Chromatoiulus* / *divaricatus* Lohm. / Опр. {Det.} Typen / Сбор. {Col.} VIII.1927, Армения {Armenian} / Делижан {Dilijan} / А. Дьяконов {A. Djakonov}.

MYR_DIP_0000215: *Chromatoiulus* / *divaricatus* n. sp. / Hans Lohmander // Зоол. Муз. АН. {Zool[ogical] Mus[eum] A[cademy] of S[ciences]} / №38-1910 г. / 774. *Chromatoiulus* / *divaricatus* n.sp. / Опр. {Det.} H. Lohmander / Сбор. {Col.} 12.IX.1909 г. Тифл. {Tiflis} / губ. {prov.} Берега Табжи- {shores of Tabzhi-} / Хурского оз. {Khursk La[ke]} у Боржо- / ма {nr Borjom} В. Милютин {V. Milyutin}.

MYR_DIP_0000216: *Chromatoiulus* / *divaricatus* n. sp. / Hans Lohmander // Зоол. Муз. АН. {Zool[ogical] Mus[eum] A[cademy] of S[ciences]} / №394-09 г. / 775. *Chromatoiulus* / *divaricatus* n.sp. / Опр. {Det.} H. Lohmander / Сбор. {Col.} 30.VII.1909 г. / Батумская обл., {Batumi Oblast} / Аджария {Adzharia}, Арспан- / ский пост {Arspansky fast} К. Сатунин {K. Satunin}.

MYR_DIP_0000267: *Chromatoiulus* / *divaricatus* n. sp. / ? Hans Lohmander // Зоол. Муз. АН. {Zool[ogical] Mus[eum] A[cademy] of S[ciences]} / №394-1909 г. / 668. *Chromatoiulus* / *divaricatus* n.sp. / Опр. {Det.} H. Lohmander ? / Сбор. {Col.} Закавказье, Арси- / анский пост, Аджары {Transcaucasia, Arsiansky fast, Adzhary} / 30.VII.1909 г. / К. Сатунин {K. Satunin [col.]}.

**Current nomenclature**. *Omobrachyiulus divaricatus* (Lohmander, 1936) (Sierwald et al. 2026). This combination was proposed by Vagalinski and Lazányi (2018) and Vagalinski and Golovatch (2021), who also revised the taxonomic status of *Omobrachyiulus* Lohmander, 1936.

**Remarks**. None of the collection dates given by Lohmander (1936: 140) correspond to those indicated on the labels. According to Lohmander, the sample MYR_DIP_0000214 should contain 4♂, but only two are available. The sample of 2♀ (MYR_DIP_0000216) was not listed by Lohmander at all, and as such cannot be considered containing syntypes. All samples contain broken specimens. In addition, two syntypes (torsos of 1♂ and 1♀) are in GMNH (MYRIA000014647; O. Gran, pers. comm., 26.I.2026).

**enghoffi Korsós, 2001 (*Anaulaciulus* )**

*Anaulaciulus enghoffi* Korsós, 2001: 78, figs 33–40 (D♂♀).

**Types. *Holotype***: (♂), MYR_DIP_0000094. ***Paratypes***: (8♂ 7♀), MYR_DIP_0000093.

**Type locality**. China, Gansu, Hezheng Co., apparently the mountain pass Lajishan (ca 35°20'45"N, 103°15'03"E).

**Label data**. MYR_DIP_0000094: Зоол. Муз. АН {Zoological Museum of the Academy of Scineces} / *Anaulaciulus* / *enghoffi* Korsós / Опр. {Det.} Korsós, 1994 / Сбор {Collected} China. 4. V.1885 / leg. G.N. Potanin / голотип ♂ / паратип 8♂ 7♀ // *Anaulaciulus enghoffi* sp. n. / Korsós, 1992 // *Anaulaciulus enghoffi* Korsós, 1994. / male holotype / China: Kansu, southern wall of the pass Latschi-san, / Karyn Valley, 4.May 1885; leg. G.N. Potanin.

MYR_DIP_0000093: Зоол. Муз. АН {Zoological Museum of the Academy of Scineces} / *Anaulaciulus* / *enghoffi* Korsós / Опр. {Det.} Korsós, 1994 / Сбор {Collected} China, 4. V.1885 / leg. G.N. Potanin / голотип ♂ / паратип 8♂ 7♀ // *Anaulaciulus enghoffi* sp. n. / Korsós, 1992 // *Anaulaciulus enghoffi* Korsós, 1994 / 8 male and 7 female paratypes. / China: Kansu, southern wall of the pass Latschi-san, / Karyn Valley, 4 May 1885; leg. G.N. Potanin // China, Kansu, südl. Hang / des Latschi-san Passes / (Karyn Tal), 4. Mai 1885 / G.N. Potanin leg. / Zool. Mus. Leningrad // *Anaulaciulus* n. sp. (Julidae). / very close to *A. inaequipes* Enghoff, 1986, / from Burma. H. Enghoff det., 1986.

**Current nomenclature**. *Anaulaciulus enghoffi* Korsós, 2001 (Sierwald et al. 2026).

**Remarks**. The remaining paratypes are in HMNH (1♂ 1♀), MNHG (1♀), SMF (1♀), ZMMU (1♂ 1♀), and NHMD (1♂ 1♀).

***exiguus* Brandt, 1840 (*Julus* )**

*Julus exiguus* Brandt, 1840c: 102 (D♂♀).

**Types. *Syntypes***: (1♂), MYR_DIP_0000027; (1♂), MYR_DIP_0000046.

**Type locality**. USA, Pennsylvania (no exact locality).

**Label data**. MYR_DIP_0000027: *Julus exiguus* Brandt, 1841 / Опр. {Det.} Brandt / Сбор {Collected} Pensylv. / Mus. Ber., 1839 / 1♂ синтип {syntype} // Зоол. Инст. АН {Zoological Institute of the Academy of Sciences} / *Brachyiulus* / *pusillus* (Leach), 1815 / Опр. С.И. Головач {Det. S.I. Golovatch} / Сбор США {Collected USA} / Пенсильвания {Pennsylvania} / leg. Zimmermann / из coll. Berl. Mus. 1839 / гоноподии залиты в препарат {gonopods mounted on a slide}.— Micropreparation: *Julus exiguus* / Brandt, 1841 / синтип {syntype} ♂ / гоноподии {gonopods} // *Brachyiulus* / *pusillus* (Leach), 1815 / 1♂ гоноподии {gonopods} / det. С. Головач {S. Golovatch}, 1979.

MYR_DIP_0000046: *Julus exiguus* / Brandt, 1841 / Опр. {Det.} Brandt. / Сбор {Collected} Pensylv. / Mus. Ber. 1839 / 1♂ синтип {syntype} // 1♂ / Зоол. Инст. АН {Zoological Institute of the Academy of Sciences} / *Brachyiulus* / *pusillus* (Leach), 1815 / Опр. С.И. Головач {Det. S.I. Golovatch} / Сбор {Collected} США, Пенсильвания {USA, Pennsylvania} / из коллекции {from the collection of} Zool. Mus // Berlin, получено в ЗИН {received in} / в 1839 г., leg. Zimmermann / 1♂.

**Current nomenclature**. Listed as accepted by Sierwald et al. (2026), but as a junior synonym of *Brachyiulus pusillus* (Leach, 1815) by Chamberlin (1921).

**Remarks**. The remaining syntypes (1♂ 5♀) are in ZMB.

***ghilarovi* Gulička, 1963 (*Julus* )**

*Julus ghilarovi* Gulička, 1963: 521, figs 1.4–7 (D♂♀).

**Type. *Holotype***: (♂), MYR_DIP_0000055.

**Type locality**. Russia, Kemerovo Oblast, Prokopyevsk (ca 53°52'N, 86°38'E).

**Label data**. *Julus ghilarovi* / Gulička / Опр. {Det.} Holotypus ♂ / Сбор {Collected} Ю.Б. Бызовой и З.В. / Чадаевой {Yu.B. Byzova and Z.B. Chadaeva} / Кемеров. обл., Прокопьевск {Kemerovo Oblast, Prokopyevsk} / гидроузел, лес {waterworks, forest} IV–V.60 // *Julus ghilarovi* n.sp. / Holotypus. Prokopyevsk // Кемеровская обл. {Kemerovo Oblast} / Прокопьевск, гидроузел, {Prokopyevsk, waterworks,} / лес. {forest} 6. IV.1960, / ♂ голотип. {holotype} / leg. Ю.Б. Бызова и З.В. Чадаева {Yu.B. Byzova and Z.B. Chadaeva} // № 917.

**Current nomenclature**. *Julus ghilarovi* Gulička, 1963 (Sierwald et al. 2026).

**Remarks**. The holotype is broken into two parts. The paratypes (4♂ 11♀) are kept embezzled in the SMNPS collection.

***ghilarovi brachydactylus* Gulička, 1972 (*Julus*)**

*Julus ghilarovi brachydactylus* Gulička, 1972: 44, fig. 5.3 (D♂).

**Type. *Holotype***: (♂), MYR_DIP_0000054.

**Type locality**. Russia, Altai Republic, Kurai (ca 50°14'N, 87°56'E).

**Label data**. *Julus ghilarovi* / *brachydactylus* Gulička / Опр. {Det.} Holotypus / Сбор Д. Берман {Collected D. Berman} / Кемеровская обл. {Kemerovo Oblast} / Ю.-В. Алтай, Курайская {SE Altai, Kurai} / котловина {Hollow} 17.VII.63 // *Iulus ghilarovi brachydactylus* / n.subsp. – Holotypus // Кемеровская обл., {Kemerovo Oblast} / Юго-Восточный Алтай, {SE Altai} / Курайская котловина. {Kurai Hollow} / 17 июля 1963 (Д. Берман) ♂ голотип {17 July 1963 (D. Berman [col.]) ♂ holotype}.

**Current nomenclature**. *Julus brachydactylus* Gulička, 1972 as a species bona fide (Nefediev 2018).

**Remarks**. The species was described from the holotype ♂ and one paratype (♂), the latter likely retained in the SMNPS collection.

***gracilis* Lohmander, 1936 (*Kubaniulus* )**

*Kubaniulus gracilis* Lohmander, 1936: 62, 38, 39 (D♂).

**Types. *Syntypes***: (1♂), MYR_DIP_0000222; (1 juv.), MYR_DIP_0000223.

**Type locality**. Russia, Adygea Republic, middle reaches of Kisha River (ca 43°52'N, 40°20'E).

**Label data**. MYR_DIP_0000222: Зоол. Муз. АН {Zoological Museum of the Academy of Sciences} / №285-1911 г. / 808. *Kubaniulus* / *gracilis* Lohm. / Опр. {Det.} Type. / Сбор {Collected} 26.IV-3.V.1911 г. Куб. / обл. средн. течение р. {Kuban Oblast, middle reaches of} / Киши {Kishi} [illegible text], горист. / берега, поросш. пихтой / буком и вязом {mountainous banks overgrown with spruce, beech and elm} [Д.] Волнух {[D.] Volnukhin} [col.] // *Kubaniulus* / *gracilis* n.sp. / Type Hans Lohmander. / [illegible text written by pencil on the opposite side].

MYR_DIP_0000223: Зоол. Муз. АН {Zoological Museum of the Academy of Sciences} / №285-1911 г. / 809. *Kubaniulus* / *gracilis* n.sp. / Опр. {Det.} H. Lohmander(?) / Сбор {Collected} 16.VII.1911 г. Куб. об. {Kuban Oblast} / поляны близ р. Шиши {glades nr Shishi River} / (прав. прит. р. Киши) {right tributary of Kishi River} / [Д.] Волнухин {[D.] Volnukhin} [col.] // *Kubaniulus* / *gracilis* n.sp. / ? Hans Lohmander.

**Current nomenclature**. *Kubaniulus gracilis* Lohmander, 1936 (Evsyukov et al. 2020; Sierwald et al. 2026).

**Remarks**. The collection date of the type series, given by Lohmander (1936: 65) as 11.V.1911, does not correspond to any of the dates indicated on the labels. The ♂ syntype (MYR_DIP_0000222) is fragmented, the second syntype is broken into two parts.

***hastatus* Lohmander, 1932 (*Leptoiulus* )**

*Leptoiulus hastatus* Lohmander, 1932a: 8, figs 5, 6, 16, 19 (D♂).

**Types. *Holotype***: (♂), MYR_DIP_0000306.

**Type locality**. Iran, East Azebaijan Prov., Hasan Beyglu (ca 38°50'N, 46°41'E).

**Label data**. *Leptouilus* / *hastatus* n. sp. / Hans Lohmander // Зоол. Муз. АН {Zoological Museum of the Academy of Sciences} / №454-1914 г. / 599. *Leptoiulus* / *hastatus* Lohm. / Опр. {Det.} Spec. Orig.! / Сбор {Collected} Сев. Персия, сел. Га- / сан-Беглю, гора Ка- / радаг. {North Persia, Hasan-Beglyu Vil., Karadagh Mt.} 5.V.1914 г. / C. Фон-Вик. {S. von Wick [col.]}.

**Current nomenclature**. *Leptoiulus hastatus* Lohmander, 1932 (Sierwald et al. 2026).

**Remarks**. Front sections of the holotype are detached and fragmented.

***hortensis* Golovatch, 1981 (*Chromatoiulus* )**

*Chromatoiulus hortensis* Golovatch, 1981: 110, figs 12–16 (D♂♀).

**Type. *Paratype***: (1♂), MYR_DIP_0000127.

**Type locality**. Abkhasia, Sukhumi Botanical Garden (ca 43°00'N, 41°01'E).

**Label data**. Зоол. Муз. АН {Zoological Museum of the Academy of Sciences} / Паратип 1♂ {paratype} / *Chromatoiulus* / *hortensis* Golovatch / Опр. {Det.} Golovatch / Сбор {Collected} Абхазия. Сухуми {Abkhazia, Sukhumi}/ Бот. сад {botanical garden} 9.X.1978 / leg. С.И. Головач {S.I., Golovatch} // *Chromatoiulus* / *hortensis* Golovatch / 1 ♂ паратип / paratype // Абхазия / Сухуми / ботанический / сад. 9.X.1978. / С.И. Головач {Abkhazia, Sukhumi, botanical garden, S.I. Golovatch}.

**Current nomenclature**. *Omobrachyiulus hortensis* (Golovatch, 1981) (Sierwald et al. 2026). Transferred to the genus *Omobrachyiulus* Lohmander, 1936 by Vagalinski and Golovatch (2021).

**Remarks**. The holotype ♂ is in ZMMU. The whereabouts of the remaining paratypes (23♂ 29♀), which should have been deposited in ZMMU, remain unknown (not found in ZMMU; A. Schileyko, pers. comm., 4.II.2026); some paratypes may also be in MIIZ.

***implicitus* Lohmander, 1936 (*Chromatoiulus* )**

*Chromatoiulus implicitus* Lohmander, 1936: 140, figs 120–122 (D♂♀).

**Types. *Syntypes***: (1♂ 1♀), MYR_DIP_0000307.

**Type locality**. Abkhazia, Gagra (ca 43°17'N, 40°16'E).

**Label data**. *Chromatoiulus* / *implicitus* n.sp. / Type Hans Lohmander. // Зоол. Муз. АН {Zoological Museum of the Academy of Sciences} / №64-1917 г. / 768. *Chromatoiulus* / *implicitus* Lohm. / Опр. {Det.} Type / Сбор {Collected} 26.V.1910 г., Закавк. / Сухумский округ / Гагры. {Trankaucasia, Sukhumi Distr., Gagra} / В. Миндгольм {V. Mindholm [col.]}.

**Current nomenclature**. *Omobrachyiulus implicitus* (Lohmander, 1936) (Sierwald et al. 2026). Transferred to *Omobrachyiulus* Lohmander, 1936 by Vagalinski and Lazányi (2018).

**Remarks**. The remaining syntype (1♀) is kept in GMNH (MYRIA000014648; O. Gran, pers. comm., 5.II.2026).

***incarnatus* Lohmander, 1932 (*Amblyiulus* )**

*Amblyiulus incarnatus* Lohmander, 1932a: 33, fig. 26 (D♀).

**Types. *Syntypes***: (3♀), MYR_DIP_0000237; (1♀), MYR_DIP_0000238.

**Type locality**. Iran, Ardabil Prov., E of Sang Abad, Bagrov-Dag Mt. (ca 37°45'N, 48°21'E).

**Label data**. MYR_DIP_0000237: Зоол. Муз. АН {Zoological Museum of the Academy of Sciences} / №454-1914 г. / 529. *Amblyiulus* / *incarnatus* Lohm. / Опр. {Det.} spec. orig. / Сбор {Collected} Сев. Персия, вост. {North Persia, east} / склон Бaгров-Дага {slope of Bagrov-Dag} / 7.VI.1914 г. / С. Фон-Вик {S. von Wick [col.]} // *Amblyiulus* / *incarnatus* n.sp. / Hans Lohmander.

MYR_DIP_0000238: Зоол. Муз. АН {Zoological Museum of the Academy of Sciences} / №454-1914 г. / 528. *Amblyiulus* / *incarnatus* Lohm. / Опр. {Det.} spec. orig.! / Сбор {Collected} Сев. Персия, Бaгров- {North Persia, Bagrov-} / Даг, вост. склон {Dag, east slope}, 7.VI. / 1914 г., С. Фон-Вик {S. von Wick [col.]} // Nord Persien: / ost abhang des / Bagrow-Dag, / Altschaly 20/VI.1914 / S. von Wick // *Amblyiulus* / *incarnatus* / n.sp. / Hans Lohmander.

**Current nomenclature**. *Syrioiulus incarnatus* (Lohmander, 1932) (Enghoff and Moravvej 2005; Vagalinski 2020; Sierwald et al. 2026). The new combination was first introduced by Mauriès (1984).

**Remarks**. Based on the original description (Lohmander 1932a: 34), the type series consisted of 2♀ collected on 19–20.VI.1914. None of the original labels fully correspond to the published dates, but the sample MYR_DIP_0000238 (1♀) was taken from exactly the same locality: Bagrow-Dag, Altschaly on 20.VI.1914, and hence could be a syntype. Two specimens in the sample MYR_DIP_0000237 are fragmented, one of which missing the head and some front segments. The ♀ syntype in the sample MYR_DIP_0000238 is broken into two parts. Two micropreparations of syntypes are in GMNH (MYRIA000014745; O. Gran, pers. comm., 26.I.2026), collected from the type locality and on the same date as was specified by Lohmander (1932a).

***jedryczkowskii* Golovatch, 1981 (*Julus* )**

*Julus jedryczkowskii* Golovatch, 1981: 105, figs 1–7 (D♂♀).

**Type. *Paratype***: (1♂), MYR_DIP_0000128.

**Type locality**. Russia, North Ossetia, Alagir Distr., Tsei Canyon (ca 42°48'N, 43°57'E).

**Label data**. Зоол. Муз. АН {Zoological Museum of the Academy of Sciences} / Паратип 1♂ {paratype} / *Julus* / *jedryczkowskii* Golovatch / Опр. {Det.} Golovatch / Сбор {Collected} Сев. Осетия {North Ossetia} / 4.VIII.1977 / leg. М. Рудаковский {M. Rudakovsky} // *Julus* sp. / 1 ♂ / Сев. Осетия {North Ossetia} / Цей {Tsei}, 4.8.77 / 50 × 50, пл. 4. {site 4} / проба 8 {sample 8} // *Julus jedryczkowskii* / Golovatch / 1♂ / паратип / + препарат {paratype + preparation} / Северная Осетия / Северо-Осетинский заповедник / Цейсское ущелье, долина / реки Цейдон: 4. VIII.1977 / leg. М. Рудаковский {North Ossetia, North-Ossetian Nature Reserve, Tsei Canyon, Tseidon R. valley // *Julus* sp.n. / 1♂ / препарат {preparation} / 1.— Micropreparation: *Julus jedryczkowskii* Gol. / 1♂ паратип {paratype} / Северная Осетия, / Северо-Осетинский / заповедник, долина / реки Цейдон, Цейсское / ущелье, подстилка, {North Ossetia, North-Ossetian Reserve, Tseidon R. valley, Tsei Canyon, in litter} / 4.8.1977 / leg. М. Рудаковский {M. Rudakovsky} / 0000-128.

**Current nomenclature**. *Julus jedryczkowskii* Golovatch, 1981 (Sierwald et al. 2026).

**Remarks**. The holotype ♂ and most paratypes (5♂ 7♀ 17 juv.; A. Schileyko, pers. comm., 4.II.2026) are deposited in ZMMU; the remaining paratype (1♂) seems to be in MIIZ. The ZISP paratype is fragmented into several pieces.

***kacheticus* Lohmander, 1936 (*Cylindroiulus* )**

*Cylindroiulus kacheticus* Lohmander, 1936: 49, figs 27–30 (D♂♀).

**Types. *Syntypes***: (2♂ 7♀), MYR_DIP_0000234; (1♂ 5♀), MYR_DIP_0000235.

**Type locality**. Georgia, Lagodekhi (ca 41°49'N, 46°16'E) and Azerbaijan, Sheki (= Nukha) (ca 41°12'N, 47°10'E). Although Read (1992: 387) reported this species to have been described from Georgia, the original type series was actually collected from two localities nearby each other in Georgia and Azerbaijan. Since a lectotype has not been designated, the type locality cannot be unambiguously selected.

**Label data**. MYR_DIP_0000234: Зоол. Муз. АН {Zoological Museum of the Academy of Sciences} / №64-1917 г. / 799. *Cylindroiulus* / *kacheticus* Lohm. / Опр. {Det.} Type. / Сбор {Collected} 21.IV.1912 г. / Тифл. {Tiflis}. / губ. Сигнахский у. {Province, Signakhsky Co.} / Лагодехи {Lagodekhi}. Л. Ланц {L. Lants [col.]} // *Cylindroiulus* / *kacheticus* n.sp. / Type Hans Lohmander.

MYR_DIP_0000235: Зоол. Муз. АН {Zoological Museum of the Academy of Sciences} / №64-1917 г. / 800. *Cylindroiulus* / *kacheticus* n.sp. / Опр. {Det.} Lohmander / Сбор {Collected} 18.IV.1912 г. Елиза- / ветин. губ. Нуха {Elizavetinsk Province, Nukha} / Л. Ланц {L. Lants [col.]} // *Cylindroiulus* / *kacheticus* n.sp. / Hans Lohmander.

**Current nomenclature**. *Cylindroiulus kacheticus* Lohmander, 1936 (Sierwald et al. 2026).

**Remarks**. Specimens in both samples are fragmented, with four specimens in the sample MYR_DIP_0000234 being headless.

***kubanus* Lohmander, 1936 (*Unciger* )**

*Unciger kubanus* Lohmander, 1936: 88, figs 69, 70 (D♂♀).

**Types. *Syntypes***: (1♂), MYR_DIP_0000249; (2♀), MYR_DIP_0000250; (1♀), MYR_DIP_0000251.

**Type locality**. Armenia, Armavir (ca 40°09'N, 44°02'E), or Russia, Krasnodar Territory, Psebai (ca 44°08'N, 40°49'E). Since a lectotype has not been designated, the type locality cannot be unambiguously selected.

**Label data**. MYR_DIP_0000249: Зоол. Муз. АН {Zoological Museum of the Academy of Sciences} / №285-1911 г. / 810. *Unciger* / *kubanus* Lohm. / Опр. {Det.} Type. / Сбор {Collected} 10.V.1911 г. / Кубанск. oб. Арма- / вир {Kubanskaya Oblast, Armavir}, Д. Волнухин. {D. Volnukhin} // *Unciger kubanus* / n.sp. / Type Hans Lohmander.

MYR_DIP_0000250: №285-1911 г. / 810. *Unciger kubanus* / n.sp. / Опр. {Det.} Lohmander / Сбор {Collected} 5.V.1911 г. / Кубан. обл. Псебай. {Kubanskaya Oblast, Psebai} / Д. Волнухин. {D. Volnukhin} // *Unciger kubanus* / n.sp. / Hans Lohmander.

MYR_DIP_0000251: №285-1911 г. / 819. *Unciger kubanus* / n.sp. / Опр. {Det.} H. Lohmander / Сбор {Collected} 1.VI.1911 г. Куб. об. {Kubanaskaya Oblast} / Ст. Псебай на под- / еме высот Герисгель. {Psebai station on raising elevations at Geriskel’} / Д. Волнухин. {D. Volnukhin} // *Unciger kubanus* / n.sp. / Hans Lohmander.

**Current nomenclature**. *Unciger kubanus* Lohmander, 1936 (Sierwald et al. 2026).

**Remarks**. Based on Lohmander (1936: 90), the sample from Armavir (MYR_DIP_0000249) also contained an adult female, which is now in GMNH (MYRIA000014769; O. Gran, pers. comm., 26.I.2026). The ♂ syntype has its head detached from the body.

***lindholmi* Lohmander, 1936 (*Julus* )**

*Julus lindholmi* Lohmander, 1936: 71, figs 42, 49, 50, 53 (D♂).

**Type. *Holotype***: (♂), MYR_DIP_0000242.

**Type locality**. Abkhazia, Tsabal (= Tsebelda) (ca 43°01'N, 41°16'E).

**Label data**. Зоол. Муз. АН {Zoological Museum of the Academy of Sciences} / №64-1917 г. / 791. *Julus* / *lindholmi* Lohm. / Опр. {Det.} Type. / Сбор {Collected} 20.V.1912 г. Запад. / Закавказье, Ольгин- / ское, Цебельда {West Transcaucasia, Olginskoe, Tsebel’da} / В. Линдгольм {V. Lindholm [col.]} // *Julus lindholmi* / n.sp. / Type Hans Lohmander.

**Current nomenclature**. *Julus lindholmi* Lohmander, 1936 (Evsyukov et al. 2018; Sierwald et al. 2026).

**Remarks**. The collection date as given by Lohmander (1936: 73), 2.VI.1912, does not correspond to that indicated on the label. The specimen is fragmented.

***polyakis* Enghoff, 1987 (*Nepalmatoiulus* )**

*Nepalmatoiulus polyakis* Enghoff, 1987: 300, figs 47, 51, 52, 145–147 (D♂).

**Type. *Holotype***: (♂), MYR_DIP_0000079.

**Type locality**. China, Sichuan, Pingwu Co., nr Suining (ca 32°24'36"N, 104°33'22"E).

**Label data**. Голотип / (+препарат G-58) / Зоол. Инст. АН СССР {Zoological Institute of the USSR Academy of Sciences} / *Nepalmatoiulus polyakis* / Enghoff, 1987 / Опр. {Det.} Enghoff, 1987 / Сбор {Collected} China, Szechuan, Lun- / -an-fu, 25.VIII.1885, leg. / N. Potanin // prep. G-58 / H. Enghoff // 795 / ♂ // China, Szechuan, / Lun-an-fu (now / nr Suining), 23.VIII. / 1885 leg. G.N. Potanin / Zool. Mus. Leningrad // *Nepalmatoiulus polyakis* Enghoff / ♂ HOLOTYPE // right ½ of collum + / sgm. 23 SEM 1986. HE.— Micropreparation: *Nepalmatoiulus polyakis* Enghoff ♂. HOLOTYPE. China, Szechuan, Lun-an-fu. Potanin // Gnathochil. right ant. / left ant., sgm. 24 with legs. Euparal. / Præp. G-58. / H. Enghoff // 00000_79.

**Current nomenclature**. *Nepalmatoiulus polyakis* Enghoff, 1987 (Sierwald et al. 2026).

**Remarks**. This species was described from the holotype ♂ alone. The holotype was broken and dried out, but moistened in lactic acid on 17.IV.2023.

***redikorzevi* Lohmander, 1936 (*Grusiniulus* )**

*Grusiniulus redikorzevi* Lohmander, 1936: 148, figs 126–130 (D♂♀).

**Types. *Syntypes***: (4♀), MYR_DIP_0000243; (1♀), MYR_DIP_0000244.

**Type locality**. Georgia: Ninotsminda (former Bogdanovka) (ca 41°44'N, 45°18'E), or Borjomi (ca 41°50'N, 43°23'E). Since no lectotype has been designated, the type locality cannot be unambiguously selected.

**Label data**. MYR_DIP_0000243: Зоол. Муз. АН {Zoological Museum of the Academy of Sciences} / №183-1909 г. / 806. *Grusiniulus* / *redikorzevi* Lohm. / Опр. {Det.} Typen. / Сбор {Collected} 21.VI.1909 г. Тифл. / губ., Ахалкалакский у. / Богдановка {Tiflis Province, Akhalkalaki Distr., Bogdanovka), Л. Берг {L. Berg [col.]}. // *Grusiniulus* / *redikorzevi* Lohm / Typen Hans Lohmander.

MYR_DIP_0000244: Зоол. Муз. АН {Zoological Museum of the Academy of Sciences} / №373-1912 г. / 807. *Grusiniulus* / *redikorzevi* Lohm. / Опр. {Det.} n.sp. / Сбор {Collected} 10.VI.1912 г. Тифл. / губ. окр. Боржома {Tiflis Province, nr Borzhom} / Л. Млокосевич {L. Mlokosevitch [col.]} // *Grusiniulus* / *redikorzevi* n.sp. / Hans Lohmander.

**Current nomenclature**. Cyphobrachyiulus (Grusiniulus) redikorzevi (Lohmander 1936) (Vagalinski and Golovatch 2021).

**Remarks**. The collection dates as given by Lohmander (1936: 152), 4.VII.1909 and 23.VI.2012, do not correspond to those on the actual labels. The sample MYR_DIP_0000243 was reported by Lohmander as containing 1♂ and 4♀, but the male is currently missing and two females in this sample are fragmented. One female syntype is in GMNH (MYRIA000014741; O. Gran, pers. comm., 26.I.2026).

***rhaphimeritus* Enghoff, 1987 (*Nepalmatoiulus* )**

*Nepalmatoiulus rhaphimeritus* Enghoff, 1987: 302, figs 47, 51, 52, 148–150 (D♂♀).

**Types. *Holotype***: (♂), MYR_DIP_0000081. ***Paratypes***: (1♀), MYR_DIP_0000082; (1♂), MYR_DIP_0000083.

**Type locality**. China, Sichuan, Kangding (=Da-tsien-lu or Tachienlu) (ca 30°03'N, 101°53'E).

**Label data**. MYR_DIP_0000081: *Nepalmatoiulus rhaphimeritus* Enghoff / ♂ HOLOTYPE // Potanin, 30.V.1893 / E vic. Urb. Da-Tzsjan / -lu (prov. Sey-Tschuan) / Cat. № 3. Spec. 3 (on the reverse side) // 806 ♂ // præp. G-62. H. Enghoff.— Micropreparation: *Nepalmatoiulus rhaphimeritus* Enghoff ♂. HOLOTYPE. Szechuan, Da-tsien-lu. Potanin. Præp. / G-62. H. Enghoff // Gnathocil. right ant., P40/dorsal ½ of sgm. 23, left ant., P41. Euparal // 00000_81.

MYR_DIP_0000082: *Nepalmatoiulus rhaphimeritus* Enghoff / 1♀ PARATYPE // Танцзинлу / (Центр. Китай). Потанинь, 93 {Da-tsien-lu, (Central China), Potanin} // 807 ♀ // præp. G-63–64. H. Enghoff.— Micropreparations (×2): *Nepalmatoiulus* / *rhaphimeritus* Enghoff ♀ / PARATYPE / Szechuan: Da-tsien- / lu. Potanin. Præp. / G-63. H. Enghoff // right vulva. / Faure-Berlese / Gold Size // 00000_82.— *Nepalmatoiulus* / *rhaphimeritus* Enghoff ♀ / PARATYPE / Szechuan, Da-tsien- / lu. Potanin. Præp. / G-64. H. Enghoff // gnathochil., right / ant., P42/midbody / dorsal limbus, / left ant., P43. / Euparal // 00000_82.

MYR_DIP_0000083: *Nepalmatoiulus rhaphimeritus* Enghoff / 1♂ PARATYPE // Тацзинлу (Сычуань) / Центр. Китай / Потанинь {Da-tsien-lu, Sichuan, Central China, Potanin} // Potanin 12.V.1893 (n. st.) / E. vic. nrb. Da-Tzsjan-lu / Cat. № 8. Speni Trig (on the reverse side) // 809 ♂.

**Current nomenclature**. *Nepalmatoiulus rhaphimeritus* Enghoff, 1987 (Sierwald et al. 2026).

**Remarks**. The remaining paratype (1♂) is kept in NHMD.

***satunini* Lohmander, 1936 (*Archileucogeorgia* )**

*Archileucogeorgia satunini* Lohmander, 1936: 95, figs 75–76 (D♂).

**Type. *Holotype***: (♂), MYR_DIP_0000252.

**Type locality**. Abkhazia, E of Sukhumi, Kelasuri River valley (ca 43°00'N, 41°05'E).

**Label data**. Зоол. Муз. АН {Zoological Museum of the Academy of Sciences} / №253-1910 г. / 804. *Archileucogeorgia* / *satunini* Lohm. / Опр. {Det.} Type. / Сбор {Collected} 19.IX.1909 г., Сухум- / ский округ, Ущ. р. / Келаcури (близ Сухума) {Sukhumi Distr., canyon of Kelasuri River (nr Sukhumi)} / К. Сатунин {K. Satunin} // *Archileucogeorgia* / *satunini* n.sp. / Type Hans Lohmander.

**Current nomenclature**. *Leucogeorgia satunini* (Lohmander, 1936) (Sierwald et al. 2026). Transferred to the genus *Leucogeorgia* Verhoeff, 1930 by Antić and Reip (2020).

**Remarks**. The collection date as given by Lohmander (1936: 96), 1.X.1909, does not correspond to that indicated on the label. The holotype is fragmented.

***schestoperovi* Lohmander, 1933 (*Peltopodoiulus* )**

*Peltopodoiulus schestoperovi* Lohmander, 1933: 62, figs 58–66 (D♂♀).

**Types. *Syntypes***: (1♂ 1♀), MYR_DIP_0000272.

**Type locality**. Turkmenistan, Firjuza (= Archabil) (ca 37°55'N, 58°05'E).

**Label data**. Зоол. Муз. АН {Zoological Museum of the Academy of Sciences} / 602. *Peltopodoiulus* / *schestoperovi* Lohm. / Опр. {Det.} spec. orig. / Сбор {Collected} Туркменистан {Turkmenistan} / Копет-даг, Фирюза {Kopet-Dag, Firyuza} / 15.IV.1929 г. // Firusa, Kopet-dag / 15.IV.29 // *Peltopodoiulus* / *schestoperovi* n.sp. / Hans Lohmander.

**Current nomenclature**. *Peltopodoiulus schestoperovi* Lohmander, 1933 (Sierwald et al. 2026).

**Remarks**. The original type series studied by Lohmander (1933: 67) contained 9♂ 14♀ and three juv. females which were collected on 15 and 27.IV.1929. Of these, only two syntypes (1♂ 1♀) are available in the ZISP collection; the remaining syntypes are in GMNH (MYRIA000014752–55; O. Gran, pers. comm., 26.I.2026): (1♂ 2♀ – torsos), 11 syntypes of unspecified sex and 11 micropreparations. The whereabouts of the remaining seven syntypes is unknown.

***serpentinus* Lohmander, 1932 (*Leptoiulus* )**

*Leptoiulus serpentinus* Lohmander, 1932a: 16, fig. 13 (D♀).

**Types. *Holotype***: (♀), MYR_DIP_0000308.

**Type locality**. Iran, Khuzestan Prov., ‘Gamdalkal’ (apparently, Dehdez; ca 31°42'N, 50°17'E).

**Label data**. *Leptoiulus* / *serpentinus* n.sp. / Hans Lohmander. // 10.IV. // Зоол. Муз. АН {Zoological Museum of the Academy of Sciences} / №97-904 г. / 601. *Leptoiulus* / *serpentinus* Lohm. / Опр. {Det.} Spec. Orig.! / Сбор {Collected} З. Персия, Арабистан, {West Persia, Arabistan} / уроч. Гамдалкал {Gamdalkal [tract]}, 10.V. / 1904 г. / Н. Зарудный {N. Zarudny}.

**Current nomenclature**. Leptoiulus (Iraniulus) serpentinus Lohmander, 1932 (Sierwald et al. 2026).

**Remarks**. The holotype ♀ is broken in four parts, with the head detached.

***subalpinus* Lohmander, 1936 (*Julus* )**

*Julus subalpinus* Lohmander, 1936: 66, figs 40, 41, 46–48, 51, 52 (D♂♀).

**Types. *Syntypes***: (1♂), MYR_DIP_0000229; (1♂), MYR_DIP_0000231; (1♂ 15♀ 18 juv.), MYR_DIP_0000226; (2♀ 3 juv.), MYR_DIP_0000227; (5♀ 11 juv.), MYR_DIP_0000228; (2♀), MYR_DIP_0000230.

**Type locality**. Russia, Krasnodar Territory, Dzhuga Mt. (ca 43°54'N, 40°25'E).

**Label data**. MYR_DIP_0000229: (no 122) Bez. Kubanj. / Berg Dzhuga, sub- / alpine Wiese, 9.VI.11 / Type-♂ D. Filatov // Зоол. Муз. АН {Zoological Museum of the Academy of Sciences} / №285-1911 г. / 795. *Julus* / *subalpinus* Lohm. / Опр. {Det.} Type ♂ / Сбор {Collected} 9.VI.1911 г. Кубанск. об. {Kubanskaya Oblast} / гора Джуга, субаль. / пийский луг {Dzhuga Mt., subalpine meadow} / Д. Филатов {D. Filatov} [col.] // *Julus subalpinus* / n.sp. Hans Lohmander.

MYR_DIP_0000231: (no 134) Bez. Kubanj. / Berg Dzhuga, sub- / alpine Wiese, 9.VI.11 / ♂ D. Filatov // Зоол. Муз. АН {Zoological Museum of the Academy of Sciences} / №285-1911 г. / 796. *Julus* / *subalpinus* n.sp. / Опр. {Det.} H. Lohmander, ♂ / Сбор {Collected} 9.VI.1911 Куб. об. {Kubanskaya Oblast} / гора Джуга, субаль. / пийский луг {Dzhuga Mt., subalpine meadow} / Д. Филатов {D. Filatov} [col.] // *Julus subalpinus* / n.sp. Hans Lohmander.

MYR_DIP_0000226: Зоол. Муз. АН {Zoological Museum of the Academy of Sciences} / №285-1911 г. / 684. *Julus subal*- / *pinus* Lohm. / Опр. {Det.} spec. orig. / Сбор {Collected} Кубанск. обл. {Kubanskaya Oblast} / субальп. луг горы {subalpine meadow of Mt.} / Джуга (бл. лагеря Бол. Бамбаки) {Dzhuga (nr camp Bolshie Bambaki)} 27.V.1911 г. / Д. Волнухин {D. Volnukhin} [col.] // Кубанск. обл. {Kubanskaya Oblast} / 27/V-1911 / Субальп. лугъ горы {subalpine meadow of Mt.} / Джуга (близ ла- / геря Б. Бамбаки) {Dzhuga (nr camp Bolshie Bambaki)} // 37 экз. {specimens} // 4 экз. взяты / для Schubert’a {4 specimens taken for Schubert} // *Julus subalpinus* / n.sp. Hans Lohmander.

MYR_DIP_0000227: Зоол. Муз. АН {Zoological Museum of the Academy of Sciences} / №285-1911 г. / 685. *Julus* / *subalpinus* Lohm. / Опр. {Det.} spec. orig. / Сбор {Collected} Кубанск. обл., го- / ра Джуга {Kubanskaya Oblast, Dzhuga Mt.}, 15.VI.1911 г. / субальпийский луг и / скалы {subalpine meadow and rocks} Д. Волнухин {D. Volnukhin} [col.] // *Julus subalpinus* / n.sp. Hans Lohmander.

MYR_DIP_0000228: Зоол. Муз. АН {Zoological Museum of the Academy of Sciences} / №285-1911 г. / 794. *Julus* / *subalpinus* / Lohm. / Опр. {Det.} Typen / Сбор {Collected} 27.V.1911 г. Кубанск. / обл. субальпийский {Kubanskaya Oblast, subalpine} / луг горы Джуга (бл. {meadow Dzhuga Mt. (nr} / лагеря Бол. Бамбаки). {camp Bolshie Bambaki.)} Д. Волнухин {D. Volnukhin} [col.] // *Julus subalpinus* / n.sp. / Typen Hans Lohmander.

MYR_DIP_0000230: Зоол. Муз. АН {Zoological Museum of the Academy of Sciences} / №285-1911 г. / 797. *Julus* / *subalpinus* n.sp. / Опр. {Det.} H. Lohmander / Сбор {Collected} 25.V.1911 г. Куб. об. {Kubanskaya Oblast} / субальпийский луг и / смешанный лес на гра- / нице луга (бл. лагеря / Б. Бамбаки) {subalpine meadow and mixed forest on the meadow edge (nr camp Bolshie Bambaki)} Д. Филатов {D. Filatov} [col.] // *Julus subalpinus* / n.sp. / Hans Lohmander.

**Current nomenclature**. *Julus subalpinus* Lohmander, 1936 (Evsyukov et al. 2018; Sierwald et al. 2026).

**Remarks**. The original type series consisted of 3♂ and 18♀ collected on 7–9.VI.1911 (Lohmander 1936: 70). Of these, torsos of six female syntypes are in GMNH (MYRIA000014746–47; O. Gran, pers. comm., 26.I.2026), with their label data clearly corresponding to those published by Lohmander (1936). As for the ZISP samples, neither the number of specimens nor the collection dates given by Lohmander (1936) correspond to those available in the collection (see above). Nevertheless, there is no doubt that at least the samples MYR_DIP_0000226, MYR_DIP_0000229, and MYR_DIP_0000231 indeed belong to the original series studied by Lohmander. All specimens in the sample MYR_DIP_0000226 are badly fragmented; although this sample contains a label stating that there were 37 specimens inside, based on the head sections of fragmented specimens we have been able to find only 34 specimens (1♂ 15♀ 18 juv.). Many specimens in the samples MYR_DIP_0000228, MYR_DIP_0000231 and MYR_DIP_0000231 are broken.

### Family Mongoliulidae

***schmidti* Golovatch, 1979 (*Skleroprotopus* )**

*Skleroprotopus schmidti* Golovatch, 1979b: 904, figs 1.1–11 (D♂).

**Types. *Holotype***: (♂), MYR_DIP_0000101. ***Paratype***: (1 juv.), same number.

**Type locality**. Russia, Maritime Territory, Nakhodka, Solyonoe Lake (ca 42°49'44"N, 132°53'06"E).

**Label data**. *Skleroprotopus* / *schmidti* Gol. / 1♂ голотип {holotype} / 1 juv. паратип {paratype} // *Sklerotopus* / *schmidti* Gol. / 1 ♂ голотип {holotype} / 1 juv. паратип {paratype} // препарат {preparation} // № 202-/901 // Корейско-Сахалинская / экспедиция 1900–01 гг. / Приморский край, / Находкинский р-он, / залив Америка, бух-/ та Козьмина, у / оз. Соленого {The Korean-Sakhalin expedition of 1900-01. Maritime Territory, Nakhodka Distr., America Gulf, Kozmina Bay, nr Solyonoye Lake}, / leg. П.Ю. Шмидт {P.Yu. Schmidt} / 12.V.1900.

**Current nomenclature**. *Skleroprotopus coreanus* (Pocock, 1895) (Sierwald et al. 2026). Synonymised with *S. coreanus* by Mikhaljova (2019).

**Remarks**. This species was described from the holotype ♂ alone. The holotype dried out, is badly damaged and mouldy; it was moistened on 26.XI.2025.

***similiserratus* Golovatch, 1979 (*Skleroprotopus* )**

*Skleroprotopus similiserratus* Golovatch, 1979b: 906, figs 2.1–8 (D♂♀).

**Types. *Holotype***: (♂), MYR_DIP_0000172. ***Paratypes***: (1♀), MYR_DIP_0000155; (3♀ 3 juv.), MYR_DIP_0000156; (2♀), MYR_DIP_0000157; (4♂ 8♀ 2 juv.), MYR_DIP_0000158; (3♂ 1♀), MYR_DIP_0000159; (1♂), MYR_DIP_0000160; (5♂ 2♀), MYR_DIP_0000161; (3♂ 1 juv.), MYR_DIP_0000162; (1♂ 1♀ 1 juv.), MYR_DIP_0000163; (1♂ 7♀), MYR_DIP_0000164; (7♂ 6♀ 1 juv.), MYR_DIP_0000165; (1♂), MYR_DIP_0000166; (1♀), MYR_DIP_0000167; (1♂ 4♀ 1 juv.), MYR_DIP_0000168; (3♂ 3♀ 1 juv.), MYR_DIP_0000169; (1♂), MYR_DIP_0000170; (1 juv.), MYR_DIP_0000171; (2 juv.), MYR_DIP_0000173; (1♀), MYR_DIP_0000174; (2♀ 3 juv.), MYR_DIP_0000175; (1 juv.), MYR_DIP_0000176; (1♀ 1 juv.), MYR_DIP_0000177; (1♂), MYR_DIP_0000178; (1♂), MYR_DIP_0000179; (1♀), MYR_DIP_0000180; (1 juv.), MYR_DIP_0000181; (1♂), MYR_DIP_0000182; (1♀), MYR_DIP_0000183.

**Type locality**. Russia, Maritime Territory, Chuguevka Distr., Verkhne-Ussuriyski Field Station (ca 44°02'16.2"N, 134°12'15.3"E).

**Label data**. MYR_DIP_0000172: *Skleroprotopus* / *similiserratus* Gol. / голотип ♂ {holotype} // Приморский край, / Чугуевский р-н / Верхне-Уссурийский / стационар, Биолого-/ почвенного института ДВНЦ, / ельник, гнилой пень, / Курчева и Михалева leg. {Maritime Territory, Chuguevka Distr., Verkhne-Ussuriyskiy Field Station, Biological and Soil Institute of the Far Eastern Branch of the Russian Academy of Sciences, spruce forest, rotten stump, leg. Kurcheva and Mikhaljova} / 19.VII.1975.

MYR_DIP_0000155: *Skleroprotopus* / *similiserratus* Gol. / паратип {paratype}; 1♀ // Приморский край, / Чугуевский р-он / Верхнеуссурийский стац. / ельник, гнилой пень {Maritime Territory, Chuguevka Distr., Verkhneussuriisky Field Station, spruce forest, rotten stump} / 2/VII–75 г.

MYR_DIP_0000156: *Skleroprotopus* / *similiserratus* / Gol. паратипы {paratypes} / 3♀, 3 juv. // Прим. кр. Чугуевск. р-н / пробн. пл. № 1–1973 / ельник, гнил. пень {Maritime Territory, Chuguevka Distr., sample site № 1–1973, spruce forest, stump} / 1.VIII.75 г.

MYR_DIP_0000157: *Skleroprotopus* / *similiserratus* Gol. / паратипы {paratypes} / 2♀ // Приморский край, / Уссурийский заповедник, сопка Грабовая, под- / стилка {Maritime Territory, Ussury Res., Grabovaya Mt., litter}, 11.VI.1976 leg. / Е.В. Михалева {E.V. Mikhaljova}.

MYR_DIP_0000158: *Skleroprotopus* / *similiserratus* / Gol. паратипы {paratypes} / 4♂ 8♀, 2 juv. // Пр. кр., Чуг. р-н, Водораздел / р. Пр[авая] Соколов[ка] и Изюбриной, / ельник-сухостой, гнил. пень {Maritime Territory, Chuguevka Distr., watershed of Pravaya Sokolvka and Izyubrinnaya rivers, dry spruce forest, rotten stump}, / 25.VII.75, М. II сб. 12. Михалёва {col. Mikhaljova}.

MYR_DIP_0000159: *Skleroprotopus* / *similiserratus* Gol. / паратипы (paratypes} / 3♂ 1♀ // Пр. кр., Чуг. р-н, Верхнеусс. / стац., пр. пл. № 1, в / грибах (грузди) и подст. {Maritime Territory, Chuguevka Distr., Verkhneussuriisky Field Station, sample site № 1, in mashrooms (milk-caps) and litter}, / 11.9.75 г.

MYR_DIP_0000160: *Skleroprotopus* / *similiserratus* Gol. / паратип 1{paratype} // Прим. кр.; Уссурийский / зап-к; Дубово-кедровый / лес; подст. {Maritime Territory, Ussury Res., mixed forest, litter}; 8.VI.76 г.

MYR_DIP_0000161: *Skleroprotopus* / *similiserratus* Gol. / паратипы {paratype}, 5♂ 2♀ / (без экзувиев {without exuviae}) // Пр. кр., Чуг. р-н, долин[ный] / ильм[овый] лес, валеж[ник] {Maritime Territory, Chuguevka Distr., valley elm forest, deadwood}, / 7.VIII.75 г. // препарат {preparation}.— Preparation: *Skleroprotopus similiserratus* Gol. ♂ паратип {paratype} // Приморский край, Чугуевский р-н, / Верхнеуссурийский стационар Биолого-почв. ин-та ДВНЦ АН СССР, / долинный ильмовый лес, валежник {Maritime Territory, Chuguevka Distr., Verkhneussuriyski Field Station of the Biology and Soil Institute of the Far Eastern Scientific Centre of the Russian Academy of Scineces, valley elm forest, dead wood}, 7.VIII.1975 (Г.Ф. Курчева, Е.В. Михалева {G.F. Kurcheva, E.V. Mikhaljova} leg.). / 0000161.

MYR_DIP_0000162: *Skleroprotopus* / *similiserratus* Gol. / паратипы {paratypes} 3♂ 1 juv. // Приморье, / Супутинский з-к {Maritime Territory, Suputinsky Res.}, / 7.IX.74 / Березанцев А.Ю. {A.Yu. Berezantsev}.

MYR_DIP_0000163: *Skleroprotopus* / *similiserratus* / Gol. паратипы {paratypes} / 1♂ 1♀, 1 juv. // Приморье, окр. ст. Хасан, / у Голубиного утеса {Maritime Territory, Khasan Railway Station, at Pigeon Cliff}, 16.10.74 / [А.Ю.] Березанцев {A.Yu. Berezantsev}.

MYR_DIP_0000164: *Skleroprotopus* / *similiserratus* / Gol. паратипы {paratypes} / 1♂ 7♀ // Приморский кр., Чугуевск. р-он, / Верхнеуссурийский стац. / Ельник, гнилой пень {Maritime Territory, Chuguevka Distr., Verkhneussuriyski Field Station, spruce forest, rotten stump}, 9.VII.75 г. / [Е.В.] Михалёва {E.V. Mikhaljova}.

MYR_DIP_0000165: *Skleroprotopus* / *similiserratus* / Gol. паратипы {paratypes} / 7♂ 6♀, 1 juv. // Приморский край, / Чугуевский р-он, / Верхнеуссурийский стац. / Ельник, гнилой пень {Maritime Territory, Chuguevka Distr., Verkhneussuriyski Field Station, spruce forest, rotten stump}, 19.VII.75 / [Е.В.] Михалёва {E.V. Mikhaljova}.

MYR_DIP_0000166: *Skleroprotopus* / *similiserratus* Gol. / паратип {paratype}; 1♂ // Прим. кр., Уссур. зап-к, / Дубово-кедр. лес, / подст. {Maritime Territory, Ussuri Res., mixed forest, litter}, 29.6.76.

MYR_DIP_0000167: *Skleroprotopus* / *similiserratus* / Gol. паратип {paratype} / 1♀ // Пр. кр., Чуг. р-н, Водораздел / кл[ючей] Березового и Безымянного, / юго-зап. склон, ельник, / гнил. пень {Maritime Territory, Chuguevka Distr., watershed of Berezovyi and Bezymyannyi creeks, spruce forest, rotten stump}, 28.VII.75, М. III сб. 16.

MYR_DIP_0000168: *Skleroprotopus* / *similiserratus* Gol. / паратипы {paratypes} / 1♂ 4, 1 juv. // Уссур. зап-к, п.п. № 2 {Ussury Res., sample site № 2}, / раск. 13, А0; 11.7.76.

MYR_DIP_0000169: *Skleroprotopus* / *similiserratus* Gol. / паратипы {paratypes} / 3♂ 3♀, 1 juv. // Приморский кр. Чугуевск. р-он, / Верхнеуссурийский стац. / Ельник, гнилой пень {Maritime Territory, Chuguevka Distr., Verkhneussuriyski Field Station, spruce forest, rotten stump}, 9.VII.75 г. / [Е.В.] Михалёва {E.V. Mikhaljova}.

MYR_DIP_0000170: *Skleroprotopus* / *similiserratus* Gol. / паратип {paratype}: 1♂ // Пр. кр., Чуг. р-н, Водоразд. / кл[ючей] Березового и Безымянного, / кам. березняк с елью, подст. {Maritime Territory, Chuguevka Distr., watershed of Berezovyi and Bezymyannyi creeks, birch-spruce forest, litter}, / 28.7.75, М. III, сб. 14.

MYR_DIP_0000171: *Skleroprotopus* / *similiserratus* Gol. / паратип {paratype}: 1 juv. // Пр. кр., Уссур. з-к, / пр.пл. № 2. подст. {Maritime Territory, Ussuri Res., sample site № 2} / 29.9.76.

MYR_DIP_0000173: *Skleroprotopus* / *similiserratus* / Gol. паратипы {paratypes} / 2 juv. // Пр. кр., Чуг. р-н, Верхнеус. / Стац. пр. пл. № 1, подст. {Maritime Territory, Chuguevka Distr., Verkhneussuri Field Station, sample site № 1, litter} / 11.9.75 г.

MYR_DIP_0000174: *Skleroprotopus* / *similiserratus* Gol. / паратип: 1 // Прим. кр. Уссур. / з-к, чернопихтар. / раск. 12 {Maritime Territory, Ussuri Res., fir forest, dig 2}: 11.7.76.

MYR_DIP_0000175: // *Skleroprotopus* / *similiserratus* Gol. / паратипы / 2 ♀, 3 juv. // Прим. кр., Уссур. / з-к, чернопихтарн. {Maritime Territory, Ussury Res., fir forest}, / раск. {dig} 13: 0–10 см; / 11.7.76 г.

MYR_DIP_0000176: *Skleroprotopus* / *similiserratus* Gol. / паратип {paratype} 1 juv. // Пр. кр., Чуг. р-н, Водоразд. р. Пр[авой] / Сокол[овки] и Изюбр[иной], ельник, гнил. / пень {Maritime Territory, Chuguevka Distr., watershed of Pravaya Sokolovka and Izyubrinnaya rivers, spruce forest, rotten stump}, 25.VII.75 г., М. II, сб. 9.

MYR_DIP_0000177: *Skleroprotopus* / *similiserratus* Gol. / паратип {paratype}: 1♀, 1 juv. // Прим. кр., Уссурийский зап-к, / грабовая сопка; гнилая / древесина {Maritime Territory, Ussuri Res., hornbeam hill, rotten wood}; 6.VI.76 г.

MYR_DIP_0000178: *Skleroprotopus* / *similiserratus* Gol. / паратип {paratype}: 1 ♂ // Приморский край, / хр. Чандолаз, близ / пещ. Близнец {Maritime Territory, Chandolaz Mt. Range, Bliznets Cave}, / 9.VI.1974 (Б.П. Чевризов {B.P. Chevrizov}).

MYR_DIP_0000179: *Skleroprotopus* / *similiserratus* Gol. / паратип {paratype}: 1♂ // Прим. кр., Уссур. / з-к, чернопихтарн. {Maritime Territory, Ussuri Res., fir forest}, / раск. {dig} № 14: А0: 11.7.76.

MYR_DIP_0000180: *Skleroprotopus* / *similiserratus* Gol. / паратип {paratype}: 1♀ // Прим. кр., Уссур. / з-к, чернопихтарник {Maritime Territory, Ussuri Res., fir forest}, / раск. {dig} № 9; А0: 7.7.76.

MYR_DIP_0000181: *Skleroprotopus* / *similiserratus* Gol. / паратип {paratype}: 1 juv. // Пр. кр., Уссур. з-к, / Дуб-кедр. лес, подстилка {Maritime Territory, Ussuri Res., mixed forest, litter}, / 12.6.76.

MYR_DIP_0000182: *Skleroprotopus* / *similiserratus* Gol. / паратип {paratype}: 1♂ // Прим. кр., Уссур. з-к, / Дубняк, подст. {Maritime Territory, Ussuri Res., oak forest, litter} 14.10.75.

MYR_DIP_0000183: *Skleroprotopus* / *similiserratus* Gol. / паратип {paratype}: 1♀ // Пр. кр., Уссур. з-к, / Дуб-кедр. лес, подст. {Maritime Territory, Ussuri Res., mixed forest, litter}, / 29.6.76.

**Current nomenclature**. *Skleroprotopus coreanus* (Pocock, 1895) (Sierwald et al. 2026). Synonymised with *S. coreanus* by Mikhaljova (1982).

**Remarks**. No paratypes of this species are currently available in ZMMU (A. Schileyko, pers. comm., 4.II.2026).

### Family Nemasomatidae

***filiformis* Enghoff, 1985 (*Basoncopus* )**

*Basoncopus filiformis* Enghoff, 1985: 32, figs 6–12 (D♂♀).

**Types. *Holotype***: (♂), MYR_DIP_0000085. ***Paratype***: (1♀), MYR_DIP_0000086.

**Type locality**. Kazakhstan, Dzungarian Alatau Mts (no exact locality).

**Label data**. MYR_DIP_0000085: *Basoncopus filiformis* Enghoff / ♂ HOLOTYPE // П 1 п 1 Дж 35–50 // præp. G-37–39 H. Enghoff (в пробирке) {in vila} // USSR, East Kazakhstan / Djungaria, Djungarsky / Alatau, old fruit garden / VIII.1966, leg. N.K. Belousova.— Micropreparations (×3): *Basoncopus filiformis* Enghoff ♂. Dzhungarskij Alatau, 1966, Belousova leg // Præp. G-37 H. Enghoff // Gonopods. Euparal. HOLOTYPE // 00000_85. – *Basoncopus filiformis* / Enghoff. HOLOTYPE ♂ / Dzhungarskij Ala / tau, 1966, Belou / sova leg // gnathochilarium P 2. / Сlypeus, antennae, right P60, P61. / Euparal // Præp.: G-38 H. Enghoff // 00000_85. – *Basoncopus filiformis* Enghoff ♂. HOLOTYPE. Dzhungarskij Alatau, 1966, Belousova leg // first pair of legs, oral view. Euparal // Præp. G-39 H. Enghoff // 00000_85.

MYR_DIP_0000086: *Basoncopus filiformis* Enghoff. / ♀ PARATYPE / USSR, East Kazakhstan / Djungaria, Djungarsky Alatau / old fruit garden. VIII.1966 / leg. N.K. Belousova // præp. G-40 ♀ H. Enghoff.— Micropreparation: *Basoncopus filiformis* Enghoff. PARATYPE ♀. Dzhungarskij Alatau, 1966, Belousova leg // P68–69: Euparal. P2 + left vulva: Faure-Berlese. Prep.: G-40 H. Enghoff // 00000_86.

**Current nomenclature**. *Basoncopus filiformis* Enghoff, 1985 (Sierwald et al. 2026).

**Remarks**. The species was only described from the holotype ♂ and paratype ♀.

***kasakstanus* Lohmander, 1933 (*Isobates* )**

*Isobates kasakstanus* Lohmander, 1933: 52, figs 48–57 (D♂♀).

**Types. *Lectotype***: (♂), MYR_DIP_0000275. ***Paralectotype***: (1♂ 2♀), same number.

**Type locality**. Uzbekistan, Andijan (ca 40°47'N, 72°18'E).

**Label data**. Зоол. Муз. АН {Zoological Museum of the Academy of Sciences} / 589. *Isobates* (*Orini* / *sobates*) *kasakstanus* Lohm. / Опр. {Det.} spec. orig.! / Сбор {Collected} Фергана, Анди- / жан {Fergana, Andizhan}, IX.1878 г. / Кушакевич {Kushakevich [col.]} Fergana, Andizhan, / IX.1878, Kushakevich // Andizhan / Kuschakevich, 18–78 // *Isobates* / *kasakstanus* n.sp. / Hans Lohmander.

**Current nomenclature**. *Orinisobates kasakstanus* (Lohmander, 1933) (Sierwald et al. 2026). Enghoff (1985) transferred the species to *Orinisobates* Lohmander, 1933.

**Remarks**. The original type series studied by Lohmander (1933: 58) contained 6♂ 2♀ and 1 juv. ♀, of which only four specimens are available: three microvials with the lectotype ♂, paralectotypes (1♂ 2♀) and some body fragments; one male paralectotype, designated by H Enghoff in 1983, is in GMNH (MYRIA000014744; O. Gran, pers. comm., 26.I.2026). Yet, the jar with the type series of *Isobates kazakstanicus* contains the second sample tube (MYR_DIP_0000276) with three specimens that were re-identified by H. Enghoff as *Orinisobates sibiricus* in 1983 (see below).

***microthylax* Enghoff, 1985 (*Orinisobates* )**

*Orinisobates microthylax* Enghoff, 1985: 49, figs 50–52 (D♀).

**Types. *Holotype***: (♀), MYR_DIP_0000072. ***Paratypes***: (1♀), MYR_DIP_0000075; (1♀), MYR_DIP_0000076; (1♀ 1 juv.), MYR_DIP_0000077; (2 ♀), MYR_DIP_0000078; (38♀ 3 juv.), MYR_DIP_0000084.

**Type locality**. Russia, Buryatia, Darkhituy [Lakes] (ca 52°32'36"N, 112°39'23"E).

**Label data**. MYR_DIP_0000072: *Orinisobates microthylax* Enghoff, 1985 / Опр. {Det.} Enghoff / А. Алексеевой {A. Alexeeva} / ГОЛОТИП ♀ / Бурятия, окр. оз. Еравное, / Дархитуй, тайга {Buryatia, nr Lake Yeravnoe, Darkhitui, taiga}, 23.VI.1975 // *Orinisobates microthylax* Enghoff / ♀ HOLOTYPE / Transbaikalia, Buryatia, vicinity / of Lake Yeravnoye, Darkhituy, / mixed forest, taiga, litter / 23.VI.1975, Leg. A. Alexeeva // præp. G-45 ♀. / H. Enghoff .— Micropreparation: *Orinisobates microthylax* Enghoff ♀. HOLOTYPE, Transbaikalia, at Lake Yeravnoye, 23.VI.1975, A. Alexeeva leg. // Præp. G-45. H. Enghoff // gnathochilarium, clypeus, antenna, P 28–29. Euparal P2 & left vulva Faure-Berlese – two preparations on the slide, printed labels (75).

MYR_DIP_0000075: Siberia, Transbaikalia, / Buryatia, vicinity of / Lake Yeravnoye, Darkhituy, taiga, / mixed forest litter, / leg. A. Alexeeva / 23.VI.1975 // 23.VI.75, Бурятия / оз. Еравное, См. лес {Buryatia, Yeravnoe lake, mixed forest} / Дархитуй 2 (А_0_) {Darkhitui} / Алексия {Alexia} / № 32 (на обороте) {on the reverse} // *Orinisobates* / *sibiricus* (?) Gul. / Det. S. Golovatch // *Orinisobates microthylax* Enghoff. ♀ PARATYPE // præp. G-46. H. Enghoff.— Micropreparation: *Orinisobates microthylax* Enghoff ♀. PARATYPE. Transbaikalia, at Lake Yeravnoye, 23.vi.1975, A. Alexeeva leg. // Præp. G-46. H. Enghoff // P2 & vulvae Faure-Berlese (75).

MYR_DIP_0000076: Kamchatka, ca. 3 km / away from Kozyrevsk / Birch-tree wood litter / 24.VI.1977 / leg. L.S. Yefremova / *Orinisobates* sp. / det. S. Golovatch // Камчатская обл. 3 км / от п. Козыревск, березняк / (береза белая), подст., {Kamtchatka oblast, 3 km off Kozyrevsk Vil., birch forest, litter} / 24.6.77 г., Ефремова Л.С. {L.S. Efremova}// *Orinisobates microthylax* Enghoff ♀ PARATYPE // præp. G-47. H. Enghoff.— Micropreparation: *Orinisobates microthylax* Enghoff ♀. PARATYPE. Kamchatka, 3 km from Kozyrevsk, 24.vi.1977. L.S. Yefremova leg // Præp. G-47. H. Enghoff // gnathochilarium, clypeus, antennae, P 34–35. Euparal P2 & vulvae Faure-Berlese (76).

MYR_DIP_0000077: Kamchatka, / Esso, River / Uksichan bank, / rotten wood, / 11.VIII.1976 / leg. G.F. Kurčeva & / E.V. Mikhaljova / *Orinisobates* sp. / det. S. Golovatch // *Orinisobates microthylax* Enghoff. / 1♀, 1 juv. PARATYPES // præp. G-48. H. Enghoff.— Microipreparation: *Orinisobates microthylax* Enghoff ♀. PARATYPE. Kamchatka, Esso 11.viii.1976. G.E. Kurcheva leg. // Præp. G-48. H. Enghoff // vulvae Faure-Berlese.

MYR_DIP_0000078: Transbaikalia, / Buryatia, / vicinity of / Lake Yeravnoye, / Darkhityu, mixed forest, / taiga, litter, / 23.VI.1975. / leg. A. Alexeeva // 23.VI.75. Бурятия. / оз. Еравное. См. лес. / Дархитуй 2 (А_0_) {Buryatia, Yeravnoe Lake, mixed forest, Darkhitui} / Алексеева {Alexeeva} // *Orinisobates microthylax* Enghoff. / 2♀ PARATYPES.

MYR_DIP_0000084: *Orinisobates* / *microthylax* Enghoff, 1985 / Опр. {Det.} H. Enghoff / Сбор {Collected} С.И. Головача и / Л.Б. Рыбалова {S.I. Golovatch and L.B. Rybalov} / паратипы / paratypes // USSR, Far East, / Vladivostok, Bot. Gardens / patch of preserved local / forest, litter & under bark, 9.VI.1984. S. Golovatch & / L. Rybalov leg // *Orinisobates microthylax* n. sp. 38 ♀ of which 2 masculinised, 3 juv. H. Enghoff det., 1985, further 5♀ retained by HE.— Micropreparations: *Orinisobates microthylax* n. sp. masculinised ♀! Vladivostok 1984 Golovatch & Rybalov PARATYPE. H. Enghoff // P1, P8, right P9 Euparal. Præp. G-54. H. Enghoff 1985 (84). – *Orinisobates microthylax* Enghoff ♀, Vladivostok 1984 Golovatch & Rybalov PARATYPE // gnathochilarium & vulvae Faure-Berlese Præp. G-55. H. Enghoff, 1985 (84) – *Orinisobates microthylax* masculinised ♀! Vladivostok 1984 Golovatch & Rybalov PARATYPE // P8, P9. Euparal. Præp. G-56. H. Enghoff, 1985.

**Current nomenclature**. *Orinisobates microthylax* Enghoff, 1985 (Sierwald et al. 2026).

**Remarks**. The remaining paratypes (6♀) are in NHMD.

***sibiricus* Gulička, 1963 (*Isobates* )**

*Isobates sibiricus* Gulička, 1963: 522, figs 2.1–2 (D♂♀).

**Type. *Syntype***: (♀), MYR_DIP_0000059.

**Type locality**. Russia, Kemerovo Oblast, Prokopyevsk (ca 53°52'N, 86°38'E).

**Label data**. *Isobates sibiricus* / Gulička / Опр. {Det.} Holotypus / Сбор {Collected} Ю.Б. Бызовой и З.В. Чадаевой {Yu.B. Byzova and Z.V. Chaadaeva} / Кемеров. обл. Прокопьевск, гидроузел / лес {Kemerovo Region, Prokop’evsk, waterworks, forest} / IV–V.1960 // Кемеровская обл., / Прокопьевск, гидроузел, / лес {Kemerovo Region, Prokop’evsk, waterworks, forest}, IV–V.1960 / ♂ голотип / leg. Ю.Б. Бызова и З.В. Чадаева {Yu.B. Byzova and Z.V. Chaadaeva} // This specimen is a ♀! / H. Enghoff, 1983 // *Isobates sibiricus* n. sp. / Prokopievsk – Holotypus.

**Current nomenclature**. *Orinisobates sibiricus* (Gulička, 1963) (Sierwald et al. 2026). Enghoff (1985) transferred this species to *Orinisobates* Lohmander, 1933.

**Remarks**. The remaining syntypes (4♂ 7♀) are kept embezzled in the SMNPS collection. Moreover, as noted by Enghoff (1985) who revised the ZISP sample, Gulička returned, after a scandal, a female he had labelled as the holotype, but since the original description (Gulička 1963) was clearly based on a male, all ZISP and SMNPS samples actually represent only syntypes. No type specimens of this species are available in ZMMU (A. Schileyko, pers. comm., 4.II.2026).

One sample with the syntypes of *Isobates kazakstanicus* (see above) was re-identified by H. Enghoff as *Orinisobates sibiricus* in 1983.

***soror* Enghoff, 1985 (*Orinisobates* )**

*Orinisobates soror* Enghoff, 1985: 48, figs 44–50 (D♂♀).

**Types. *Holotype***: (♂), MYR_DIP_0000070. ***Paratypes***: (2♂ 3♀), MYR_DIP_0000071.

**Type locality**. Russia, Kunashir Island, coordinates are given for Yuzhno-Kurilsk (ca 44°03'N, 145°51'E).

**Label data**. MYR_DIP_0000070: *Orinisobates soror* Enghoff / ♂ HOLOTYPE // Soviet Far East, the Kuril / Islands, Kunashyr Isle / seashore of the Okhotsk Sea, / in the pebble & under stones / 17.VIII.1975, leg. G.F. Kurčeva // præp. G-43 H. Enghoff.— Micropreparation: *Orinisobates soror* Enghoff, HOLOTYPE ♂ Kuril isl. (Kunashyr), 17.VII.75, G. Kurcheva leg // gnathochilarium P 1,2,8,9. Clypeus, antennae P 36–37. Euparal (70) // Præp. G-43 H. Enghoff // 00000_70 – two preparations on the same slide, printed labels.

MYR_DIP_0000071: Soviet Far East, / Kuril Islands, / Kunashir Isle, / seashore of the / Okhotsk Sea, / in pebble & / under stones, / leg. G.F. Kurčeva. / 17.VII.1975 // præp. G-44 ♀ / H. Enghoff.— Micropreparation: *Orinisobates soror* Enghoff, PARATYPE ♀, Kuril isl. (Kunashir), 17.VII.75, G. Kurcheva leg. // clypeus, antennae P 38–39. Euparal, P2 & vulvae Faure-Berlese (71) // Præp. G-44 H. Enghoff // 00000_71 – two preparations on the same slide, printed labels.

**Current nomenclature**. *Orinisobates soror* Enghoff, 1985 (Sierwald et al. 2026).

**Remarks**. The remaining paratypes (1♂ 1♀) are in NHMD.

### Order Platydesmida

**Family Platydesmidae**

***grayi* Golovatch & Korotayeva, 2026 (*Platydesmus* )**

*Platydesmus grayi* Golovatch & Korotayeva, 2026: 149, figs 1–15 (D♂♀).

**Type. *Holotype***: (♂), MYR_DIP_0000280. ***Paratypes***: (7♀), MYR_DIP_0000281; (1♂ 1♀), MYR_DIP_0000301.

**Type locality**. Costa Rica, Heredia Prov., La Selva Biological Station (10°25'58.9"N, 84°00'24.4"W).

**Label data**. MYR_DIP_0000280: ♂ holotype // *Platydesmus grayi* sp.n. / ♂ holotype / det. S. Golovatch & A. Korota- / yeva 2026 // Costa Rica, Heredia, La / Selva Biological Station, / 10°25'58.9"N, 84°00'24.4"W / 23–30.VI.2025, A. Gray.

MYR_DIP_0000281: *Platydesmus grayi* sp.n. / 7♀ paratypes / det. S. Golovatch & A. Korota- / yeva 2026 // Costa Rica, Heredia, La / Selva Biological Station, / 10°25'58.9"N, 84°00'24.4"W / 17.VII.2025, A. Gray.

MYR_DIP_0000301: *Platydesmus grayi* sp.n. / 1♂1♀ paratypes / det. S. Golovatch & A. Korotae- / eva 2026 // Costa Rica, Heredia, La / Selva Biological Station, / 10°25'58.9"N, 84°00'24.4"W / 23–30.VI.2025, A. Gray.

**Current nomenclature**. *Platydesmus grayi* Golovatch & Korotayeva, 2026 (Golovatch and Korotayeva 2026).

**Remarks**. The remaining paratypes (1♂ 1♀) are in ZMMU. Specimens in the sample MYR_DIP_0000301 are fragmented.

### Order Polydesmida

**Family Dalodesmidae**

***capensis* Brandt, 1841 (*Polydesmus* )**

*Polydesmus capensis* Brandt, 1841d: 10 (D♀).

**Type. *Lectotype***: (♀), MYR_DIP_0000049.

**Type locality**. South Africa, Cape of Good Hope (no exact locality).

**Label data**. *Polydesmus capensis* / Brandt, 1841 / ♀ lectotype / design. S. Golovatch, / 1999 // Africa aust. Br // *Polydesmus capensis* / Nob. ap. Afr. Brandt // Lectotypus / design.

**Current nomenclature**. *Polydesmus capensis* Brandt, 1841 (Sierwald et al. 2026). The lectotype was designated by Golovatch and Hoffman (2000). Superficially, this species may belong to the genus *Allawrencius* Verhoeff, 1939, formally being transferred and referred to as ?*Allawrencius capensis* (Brandt, 1841), comb. nov. ex *Polydesmus* Latreille, 1802/03 (det. SIG).

**Remarks**. This species was described from three specimens (Brandt 1841d), of which only one is now available in the ZISP collection; the whereabouts and sex of the other two remain unknown. The lectotype is broken.

***hamatus* Brandt, 1841 (*Polydesmus* )**

*Polydesmus hamatus* Brandt, 1841d: 10 (D♀).

**Type. *Holotype***: (♀), MYR_DIP_0000028.

**Type locality**. Madagascar (no exact locality).

**Label data**. *Polydesmus hamatus* / Brandt / Опр. {Det.} Brandt / Сбор {Collected} Madagascar / Dupont / голотип / holotypus // 162 // *Dalodesmus hamatus* / (Brandt, 1841) / 1 ♀: det. S. Golovatch, 1988 // *P. hamatus* / Madagascar / 1839, Dupont.

**Current nomenclature**. *Dalodesmus hamatus* (Brandt, 1841) (Wesener et al. 2025; Sierwald et al. 2026). Transferred to the genus *Dalodesmus* Cook, 1896 by Golovatch and Hoffman (1989).

**Remarks**. This species was described from the holotype ♀ alone. The holotype is broken.

### Family Opisotretidae

***himalayensis* Golovatch, 1987 (*Martensodesmus* )**

*Martensodesmus himalayensis* Golovatch, 1987: 205, figs 3–6 (D♂).

**Types. *Paratypes***: (1♂), MYR_DIP_0000142; (1♂), MYR_DIP_0000143.

**Type locality**. Nepal, between Dobhan (Dhading/Gorkha border; ca 28°11'N, 84°53'E) and Jagat (Gorkha Distr.; ca 28°18'N, 84°51'E).

**Label data**. MYR_DIP_0000142: *Martensodesmus himalayensis* / Golovatch, n.g., n.sp. / 1♂ paratype, паратип / гоноподии утеряны {gonopods lost} / det. S. Golovatch, 1984 // Nepal // 222 Dhading / Gorkha Dist. Buri / Gandaki, Dobhan-Jagat / 1100–1300 m, / 30 Jul 83 Laub-wald. MARTENS & SCHAWALLER.

MYR_DIP_0000143: *Martensodesmus himalayensis* / Golovatch, n. sp. / 1♂ паратип, paratype / взято за обработку {retained for help with ID} / det. С. Головач, 1984 // 223a Dhading Dist., Buri / Gandaki, gegenüber Pang / -shing 1750 m Gebüsch, Bambus / 31 Jul 83 MARTENS & SCHAWALLER // Nepal.

**Current nomenclature**. *Martensodesmus himalayensis* Golovatch, 1987 (Sierwald et al. 2026).

**Remarks**. The holotype ♂ and the remaining paratypes (4♂) are in SMF.

### Family Paradoxosomatidae

***bucharensis* Lohmander, 1933 (*Orthomorpha* )**

*Orthomorpha bucharensis* Lohmander, 1933: 31, figs 16, 17 (D♀).

**Type. *Holotype***: (♀), MYR_DIP_0000211.

**Type locality**. Uzbekistan, Bukhara (ca 39°46'N, 64°26'E); no particular locality ‘Chil’-Dara’ has been found.

**Label data**. ? *Orthomorpha* / *bucharensis* n.sp. / Hans Lohmander // №90-97 // 603 ? *Orthomorpha* / *bucharensis* Lohm. / spec. orig. / Бухара, Чиль- / дара {Bukhara, Chil’-Dara} / А.Н. Казнаков {[leg.] A.N. Kaznakov} / Зоол. Муз. АН / №90-97 г. // 603. 1 голотип {holotype} / *Hedinomorpha bucharensis* / (Lohmander, 1933) / Опр. {Det.} С.И. Головач {S.I. Golovatch} / Сбор Бухара, Чиль-Дара, {col. Bukhara, Chil’-Dara} / leg. А. Казнаков {A. Kaznakov} / Зоол. Институт АН СССР {Zoological Institute Academy of Sciences of the USSR} / №90-97 г.

**Current nomenclature**. *Hedinomorpha bucharensis* (Lohmander, 1933) (Sierwald et al. 2026). Jeekel (1988) transferred this species to *Hedinomorpha* Verhoeff, 1934.

**Remarks**. This species was described from the holotype ♀ alone.

***complex* Golovatch, 2013 (*Sigipinius* )**

*Sigipinius complex* Golovatch, 2013: 323, figs 91–104 (D♂♀).

**Types. *Paratypes***: (1♂ 1♀), MYR_DIP_0000184; (1♂ 2♀), MYR_DIP_0000185.

**Type locality**. China, Sichuan, S of Muli (27°45'24"N, 101°15'59"E) (Golovatch 2013).

**Label data**. MYR_DIP_0000184: *Sigipinius complex* Golovatch // China, Sichuan Province / S of Muli, 3975–4120 m / 27°43'16"N/101°15'13"E / 27°43'03"N/101°15'05"E / 22.05.2012 Belousov / Davidian, Kabak, Korolev lg. // *Sigipinius complex* / Golovatch n.sp. / 1♂ 1♀ paratypes / det. S. Golovatch, 2013 // ZIN.

MYR_DIP_0000185: *Sigipinius complex* Golovatch // China, Sichuan Province / S of Muli, 3975–4120 m / 27°43'16"N/101°15'13"E / 27°43'03"N/101°15'05"E / 22.05.2012 Belousov / Davidian, Kabak, Korolev lg. // *Sigipinius complex* / Golovatch n.sp. / 1♂ 2♀ paratypes / det. S.Golovatch, 2013 // ZIN.

**Current nomenclature**. *Sigipinius complex* Golovatch, 2013 (Sierwald et al. 2026).

**Remarks**. The holotype ♂ and the remaining paratypes (4♂ 5♀) are in ZMMU.

***crassipes* Golovatch, 1984 (*Tylopus* )**

*Tylopus crassipes* Golovatch, 1984b: 62, figs 14–16 (D♂♀).

**Type. *Paratype***: (1♂), MYR_DIP_0000133.

**Type locality**. Vietnam, Lao Cai Prov., nr Sa Pa (ca 22°20'N, 103°50'E).

**Label data**. *Tylopus crassipes* / Golovatch / Опр. {Det.} Golovatch / Сбор {Collected} Vietnam, № 85б / leg. Topal-Matskási, 1971 / 1♂ паратип // *Tylopus crassipes* Golovatch / 1♂ paratype – паратип / взято на обработку из Вен- / герского музея (Будапешт) {retained from the Hungarian Natural History Museum (Budapest) for help with ID} / 1 гоноподий вычленен {one gonopod is dissected out} // Vietnam, № 85 / 1971, leg. Topál-Matskási // gonopods.

**Current nomenclature**. *Tylopus crassipes* Golovatch, 1984 (Sierwald et al. 2026).

**Remarks**. The holotype ♂ and two paratypes (1♂ 1♀) are in HMNH.

***distinctum* Golovatch, 1984 (*Substrongylosoma* )**

*Substrongylosoma distinctum* Golovatch, 1984c: 345, figs 38–42 (D♂♀).

**Types. *Paratypes***: (2♂ 2♀), MYR_DIP_0000120.

**Type locality**. India, Darjeeling, nr North Point (ca 27°04'N, 88°15'E).

**Label data**. *Substrongylosoma* / *distinctum* Golovatch / Опр. {Det.} Golovatch / Сбор {Collected} India № 857 / leg. Topál, 1967 / 2♂ 2♀ паратипы {paratypes} // *Substrongylosoma* / *distinctum* Golovatch / 2♂ 2♀ паратипы / paratypes // Взято за обработку из / коллекции Венгерского / музея естеств. истории / (Будапешт) {retained from the Hungarian Natural History Museum (Budapest) for help with ID} // № 857 / India, Topál.

**Current nomenclature**. *Substrongylosoma distinctum* Golovatch, 1984 (Sierwald et al. 2026).

**Remarks**. The holotype ♂ and the remaining paratypes (30♂ 28♀) are in HMNH. The ZISP sample dried out and was moistened on 26.XI.2025.

***erectispina* Golovatch, 2012 (*Inversispina* )**

*Inversispina erectispina* Golovatch, 2012: 4, figs 13–21 (D♂♀).

**Types. *Paratypes***: (1♀), MYR_DIP_0000187; (2♂), MYR_DIP_0000188.

**Type locality**. China, Sichuan, SW of Mianning (28°13'45"N, 101°43'07"E) (Golovatch 2012).

**Label data**. MYR_DIP_0000187: *Inversispina* / *erectispina* / Golovatch, n.sp. / 1♀ paratype / det. S. Golovatch, // China, Sichuan Prov., / Pass Pingchuan-Yanyuan / 3205 m, 27°31'53"N, / 101°42'43"E, 20.07.2011 / leg. I. Belousov & I. Kabak, 2011.

MYR_DIP_0000188: *Inversispina* / *erectispina* / Golovatch, n.sp. / 2♂ paratype / det. S. Golovatch, 2011 // China, Sichuan Prov., / SW Mianning, 3715 m, 28°14'19"N, 101°42'48"E / 08.07.2011, leg. I. Belousov / & I. Kabak // 8.07.2011 Sichuan / SW Minning, 3715 m / 28°14'19"N, 101°42'48"E.

**Current nomenclature**. *Inversispina erectispina* Golovatch, 2012 (Sierwald et al. 2026).

**Remarks**. The holotype ♂ and several paratypes (7♂ 5♀) are in ZMMU, the remaining paratypes are in MNHN (1♂) and NHMD (2♂). The ZISP sample MYR_DIP_0000188 contains two vials: one vial with gonopods, the other with the specimen.

***falcatum* Golovatch, 1984 (*Substrongylosoma* )**

*Substrongylosoma falcatum* Golovatch, 1984c: 346, figs 43–46 (D♂♀).

**Type. *Paratype***: (1♂), MYR_DIP_0000126.

**Type locality**. India, Darjeeling, nr North Point (ca 27°04'N, 88°15'E).

**Label data**. ♂ паратип / *Substrongylosoma* / *falcatum* Golovatch / Опр. {Det.} Golovatch / Сбор {Collected} India. № 843 / leg. Topál, 1967 // gonopod // взято за обработку из / Венгерского муз. ест. / истории (Будапешт) {retained from the Hungarian Natural History Museum (Budapest) for help with ID} // *Substrongylosoma* / *falcatum* Golovatch. / 1♂ (без IV сегмента) {without segment IV} / Паратип; paratype / India, № 843, Topál.

**Current nomenclature**. *Substrongylosoma falcatum* Golovatch, 1984 (Sierwald et al. 2026).

**Remarks**. The holotype ♂ and the remaining paratypes (2♂ 5♀) are in HMNH.

***granulatus* Golovatch, 1984 (*Tylopus* )**

*Tylopus granulatus* Golovatch, 1984b: 68, figs 24–26 (D♂).

**Type. *Paratype***: (1♂), MYR_DIP_0000107.

**Type locality**. Vietnam, Ninh Binh Prov., Cuc Phuong (ca 20°19'N, 105°37'E).

**Label data**. *Tylopus granulatus* / Golovatch / Опр. {Det.} Golovatch / Сбор {Collected} Vietnam / № 385, leg. Topál, 1966 // gonopods // *Tylopus granulatus* Golovatch / 1♂ paratype – паратип / Взято на обработку из Вен- / герского музея (Будапешт) {retained from the Hungarian Natural History Museum (Budapest) for help with ID} // Vietnam, № 385 / 1966, leg. Topál.

**Current nomenclature**. *Tylopus granulatus* Golovatch, 1984 (Sierwald et al. 2026).

**Remarks**. The holotype ♂ and one paratype (♂) are in HMNH.

***hilaroides* Golovatch, 1984 (*Tylopus* )**

*Tylopus hilaroides* Golovatch, 1984b: 58, figs 7, 8 (D♂).

**Type. *Paratype***: (1♂), MYR_DIP_0000122.

**Type locality**. Vietnam, Ninh Binh Prov., Cuc Phuong (ca 20°19'N, 105°37'E).

**Label data**. *Tylopus hilaroides* / Golovatch. / Опр. {Det.} Golovatch. / Сбор {Collected} Vietnam, № 385, / leg. Topál, 1966 // *Tylopus hilaroides* Gol. / 1♂ – паратип (paratypes) / Взято за обработку из / Венгерского музея естеств. / истории (Будапешт) {retained from the Hungarian Natural History Museum (Budapest) for help with ID} // Vietnam, 1966 / № 385, leg. Topál // gonopod.

**Current nomenclature**. *Tylopus hilaroides* Golovatch, 1984 (Sierwald et al. 2026).

**Remarks**. The holotype ♂ and one paratype (♂) are in HMNH.

***horridum* Golovatch, 1984 (*Polydrepanum* )**

*Polydrepanum horridum* Golovatch, 1984c: 333, figs 12–17 (D♂♀).

**Type. *Paratype***: (1♂), MYR_DIP_0000132.

**Type locality**. India, Maharashtra, Wenchi (apparently, Wani; ca 20°03'N, 78°57'E).

**Label data**. *Polydrepanum horridum* / Golovatch / Опр. {Det.} Golovatch / Сбор {Collected} India, №612 / leg. Topal, 1967 // gonopod // *Polydrepanum horridum* / Golovatch, 1♂ паратип, paratype / India, № 612, leg. Topál / взято за обработку из Венгерского / Музея ест. ист. (Будапешт) {retained from the Hungarian Natural History Museum (Budapest) for help with ID}.

**Current nomenclature**. *Polydrepanum horridum* Golovatch, 1984 (Sierwald et al. 2026).

**Remarks**. The holotype ♂ and the remaining paratypes (2♂ 5♀) are in HMNH.

***kabaki* Golovatch, 2014 (*Tylopus* )**

*Tylopus kabaki* Golovatch, 2014a: 13, figs 53–65 (D♂♀).

**Types. *Paratypes***: (1♂ 2♀), MYR_DIP_0000210.

**Type locality**. China, Yunnan, Tuoxia Highway (27°42'50"N, 99°11'27"E) (Golovatch 2014a).

**Label data**. *Tylopus kabaki* / Golovatch, n.sp. / 1♂ 2♀ paratypes / det. S. Golovatch / 2013 // 3♀ // 10.06.2013 / 2 NT // CH, Yunnan, Deqen, Tuoxia Highway / R. valley S of Geduolouo Village / 27°43'35"N, 99°11'19"E / 10.06.2013, H-3575 m / Belousov, Kabak, Davidian leg.

**Current nomenclature**. *Tylopus kabaki* Golovatch, 2014 (Sierwald et al. 2026).

**Remarks**. The holotype ♂ and the remaining paratypes (1♂ 1♀) are in ZMMU. The ZISP paratypes dried out and were moistened on 23.X.2024.

***logunovi* Golovatch, 2024 (*Touranella* )**

*Touranella logunovi* Golovatch, 2024: 408, figs 4–12 (D♂).

**Type. *Holotype***: (♂), MYR_DIP_0000205.

**Type locality**. Vietnam, Bidoup – Nui Ba National Park (12°10'38"N, 108°40'49"E) (Golovatch 2024).

**Label data**. *Touranella logunovi* / Golovatch, n.sp. / ♂ holotype / S. Golovatch det., 2024 // 14-2023, ZISP / Vietnam, Lam Dong Prov., / Lac Durong Distr., Bidoup – Nui / Ba Nat. Park, / nr field station, / 12°10'38.47"N, 108°40'49.28"E, / 1450–1500 m a.s.l., various / collecting methods, / 26.X–11.XI.2023, D.V. Logunov.

**Current nomenclature**. *Touranella logunovi* Golovatch, 2024 (Sierwald et al. 2026).

**Remarks**. The species was described from the holotype ♂ alone.

***mandshuricus* Golovatch, 1978 (*Sichotanus* )**

*Sichotanus mandshuricus* Golovatch, 1978: 681, figs 3.1–4 (D♂♀).

**Types. *Holotype***: (♂), MYR_DIP_0000129. ***Paratype***: (1♀), MYR_DIP_0000131.

**Type locality**. China, Heilongjiang, Harbin, Yimianpozhen (ca 45°04'N, 128°05'E).

**Label data**. MYR_DIP_0000129: *Sichotanus* / *mandshuricus* Golovatch / Опр. {Det.} Golovatch / Сбор {Collected} К Западу от / ст. Имяньпо Кит / А. Емельянов {W of Yimianpo station, China, A. Emeljanov} // 16.VI. [19]11-г. К Западу от ст. / Имяньпо Кит. 13 ж. д. / под корой и под камнями / А. Емельянова {W of Yimianpo Station, Chinese Railway, under bark and stones, A. Emeljanov} // № 134–912 // *Sichotanus mandshuricus* / Golovatch / голотип ♂ {holotype} / det. С. Головач {S. Golovatch} 1975.

MYR_DIP_0000131: *Sichotanus* / *mandshuricus* Golovatch / Опр. {Det.} Golovatch / Сбор {Collected} Маньчжурия / ст. Имяньпо КЖД / А. Емельянов {Manchuria, W of Yimianpo Chinese Railway Station, A. Emeljanov} // № 176–915 / *Sichotanus mandshuricus* / Golovatch / паратип ♀ {paratype} / det. С. Головач 1975 {Golovatch} // 14.VII.1914 г. Маньчжурия, / ст. Имяньпо К.В.Ж.Д. / leg. А. Емельянов {Manchuria, W of Yimianpo Chinese Railway Station, leg. A. Emeljanov}.

**Current nomenclature**. *Sichotanus eurygaster* Attems, 1898 (Nguyen and Sierwald 2013). Synonymised with *S. eurygaster* by Mikhaljova (1982).

**Remarks**. The entire type series is in ZISP.

***muminabadensis* Gulička, 1972 (*Orthomorpha* )**

*Orthomorpha muminabadensis* Gulička, 1972: 43, fig. 5.1 (D♂).

**Type. *Holotype***: (♂), MYR_DIP_0000061.

**Type locality**. Tajikistan, Muminabad (ca 38°06'N, 70°02'E).

**Label data**. *Orthomorpha muminabadensis* / Gulička / Опр. {Det.} Holotypus ♂ / Сбор {Collected} Б. Валиахмедова {B. Valiakhmedov} / Таджикистан / Муминабад {Tajikistan, Muminabad}, 30.IV.62 // Таджикистан, / близ г. Муминабад {Tajikistan, nr Muminabad Torwn}, / 30.IV.1962 / ♂ голотип {holotype} / leg. Б. Валиахмедов {B. Valiakhmedov} // *Orthomorpha muminabadensis* n. sp. / Muminabad – Holotypus.

**Current nomenclature**. *Hedinomorpha bucharensis* (Lohmander 1933) (Nguyen and Sierwald 2013). Jeekel (1988) synonymised *O. muminabadensis* (Gulička, 1972) with *bucharensis* and transferred the latter species to *Hedinomorpha* Verhoeff, 1934.

**Remarks**. Although the species was described from the holotype ♂ and three male paratypes, only the holotype is available in the ZISP collection; the holotype is broken in two parts. The paratypes remain embezzled in the SMNPS collection.

***procurvus* Golovatch, 1984 (*Tylopus* )**

*Tylopus procurvus* Golovatch, 1984b: 64, figs 17–19 (D♂♀).

**Types. *Paratypes***: (1♂ 1♀), MYR_DIP_0000154.

**Type locality**. Vietnam, pass between Lao Cai and Lai Chau provinces (ca 22°21'N, 103°46'E).

**Label data**. *Tylopus procurvus* Golovatch // Опр. {Det.} Golovatch / Сбор {Collected} Vietnam, № 116, Topál-Matskási, 1971, 1♂ 1♀ паратипы // *Tylopus procurvus* Golovatch / 1♂ 1♀ паратипы – paratypes / Взято за обработку из Венгерского / музея (Будапешт) {retained from the Hungarian Natural History Museum (Budapest) for help with ID} // Vietnam, № 116 / 1971, leg. Topál-Matskási.

**Current nomenclature**. *Tylopus procurvus* Golovatch, 1984 (Sierwald et al. 2026).

**Remarks**. The holotype ♂ and the remaining paratypes (2♂ 4♀) are in HMNH. The ZISP sample dried out and was moistened in lactic acid on 23 May 2023.

***spiniger* Golovatch, 2014 (*Sigipinius* )**

*Sigipinius spiniger* Golovatch, 2014а: 3, figs 4–13 (D♂).

**Type. *Paratype***: (1♂), MYR_DIP_0000209.

**Type locality**. China, Yunnan, WSW of Edi (27°19'31"N, 99°51'27"E) (Golovatch 2014а).

**Label data**. *Sigipinius spiniger* / Golovatch, n.sp. / 1♂ paratype / det. S. Golovatch, 2013 // CH, Yunnan, Lijiang-Shangrila, 214 Ntn Road / WSW of Edi Vilage / 27°20'35"N/99°31'58"E / 31.05.2013, H-3690 m / Belousov, Kabak, Davidian leg.

**Current nomenclature**. *Sigipinius spiniger* Golovatch, 2014 (Sierwald et al. 2026).

**Remarks**. The holotype ♂ and the remaining paratypes (4♂) are in ZMMU.

***spiralis* Golovatch, 1984 (*Vietnamorpha* )**

*Vietnamorpha spiralis* Golovatch, 1984b: 55, figs 1–3 (D♂♀).

**Types. *Paratypes***: (1♂ 1♀), MYR_DIP_0000098.

**Type locality**. Vietnam, Ninh Binh Prov., Cuc Phuong (ca 20°19'N, 105°37'E).

**Label data**. *Vietnamorpha* / *spiralis* Golovatch / 1♂ 1♀ паратипы {paratypes} / Опр. {Det.} Golovatch / Сбор {Collected} Vietnam, / № 385, leg. Topál, / 1966 // Vietnam, № 385 / 1966. leg. Topál // *Vietnamorpha spiralis* / Golovatch, sp. n. / 1♂ 1♀ паратип – paratypes / взято за обработку из коллекции / Венгерского естеств.-исторического музея / (Будапешт), Головач {retained from the Hungarian Natural History Museum (Budapest) for help with ID, Golovatch}, 1982.

**Current nomenclature**. *Vietnamorpha spiralis* Golovatch, 1984 (Sierwald et al. 2026).

**Remarks**. The holotype ♂ and the remaining paratypes (8♂ 5♀ 5 juv.) are in HMNH.

***stragulatum* Lohmander, 1932 (*Strongylosoma* )**

*Strongylosoma stragulatum* Lohmander, 1932a: 4, fig. 1 (D♂).

**Type. *Holotype***: (♂), MYR_DIP_0000269.

**Type locality**. Iran, Ardabil Prov., E of Sang Abad, Bagrov-Dag Mt. (ca 37°45'N, 48°21'E).

**Label data**. Зоол. Муз. АН {Zoological Museum of the Academy of Sciences} / №306-15 г. / 628. *Strongylosoma* / *stragulatum* Lohm. / Опр. {Det.} spec. orig. / Сбор {Collected} С. Персия, Багров-Даг, {North Persia, Bagrov-Dag} / вост. скл. коч. Алчалы {E slope of Alchaly} / 5.VII.1914 г. / С. Фон-Вик {S. von Wick [col.]} // Nord Persien / Gst.-Aghrug des / Bagrow-Dag, Alt- / Salueg- 18.VII.1914 / [on opposite side of the label]: S. V. Wick // *Strongylosoma* / *stragulatum* n.sp. / Hans Lohmander.

**Current nomenclature**. *Strongylosoma lenkoranum* Attems, 1898 (Sierwald et al. 2026). Synonymised with *S. lenkoranum* by Attems (1937).

**Remarks**. The collection date as given by Lohmander (1932a: 4), 19.VII.1914, does not correspond to the dates indicated on the actual labels. The holotype is incomplete, badly damaged, and fragmented.

### Family Platyrhacidae

***truncatus* Golovatch, Korotayeva & Martínez-Torres, 2024 (*Barydesmus* )**

*Barydesmus truncatus* Golovatch et al., 2024: 426, figs 1, 4 (D♂♀).

**Types. *Holotype***: (♂), MYR_DIP_0000192. ***Paratype***: (1♀), MYR_DIP_0000213.

**Type locality**. Costa Rica, San José Prov., Macaw Lodge (9°43'42"N, 84°31'07"W) (Golovatch et al. 2024).

**Label data**. MYR_DIP_0000192: *Barydesmus truncatus* / Golovatch & Korotaeva, n.sp. / ♂ holotype / det. S. Golovatch & Korotayeva 2026 // Costa Rica, San José Prov. / Carara, Macaw Lodge / 9°43'42.7'‘N, 84°31'07.1'‘W / 11.–18.6.2018, D.V. Logunov.

MYR_DIP_0000213: *Barydesmus truncatus* / Golovatch & Korotaeva, n.sp. / ♀ paratype / det. S. Golovatch & A. Korotayeva 2026 // Costa Rica, San José Prov. / Carara, Macaw Lodge / 9°43'42.7"N, 84°31'07.1"W / 11–18.6.2018, D.V. Logunov.

**Current nomenclature**. *Barydesmus truncatus* Golovatch & Korotaeva, 2024 (Sierwald et al. 2026).

**Remarks**. The holotype is broken into two pieces, with the gonopods extracted and kept in a separate microvial.

### Family Polydesmidae

***aberrans* Mikhaljova in Golovatch & Mikhaljova, 1979 (*Uniramidesmus* )**

*Uniramidesmus aberrans* Golovatch & Mikhaljova, 1979: 835, figs 2.5–9 (D♂♀).

**Type. *Holotype***: (♂), MYR_DIP_0000087.

**Type locality**. Russia, Vladivostok, Botanical Garden of the Institute FEB RAS (ca 43°13'N, 132°00'E).

**Label data**. *Uniramidesmus* / *aberranus* Mikhaljova / Опр. {Det.} det. Е.В. Михалёва {E.V. Mikhaljova} / Сбор {Collected} Приморский край, / Владивосток, лесопарк ДВНЦ / 13-й км. подстилка: 7.V.1977 {Maritime Territory, Vladivostok, woodland park of the Far Eastern Scientific Centre, 13^th^ km, litter} / (leg. Е.В. Михалёва). {E.V. Mikhaljova} / голотип / holotype / 25 // Приморский край, / г. Владивосток, лесо – / парк ДВНЦ АН СССР/ 13й км, подстилка / 7.5.1977 г., leg. Михалёва Е.В. {Maritime Territory, Vladivostok, woodland park of the Far Eastern Scientific Centre, 13^th^ km, in litter, leg. E.V. Mikhaljova} / *Uniramidesmus aberranus* Mikh. 1 ♂ голо – / тип. Holotypus // Diplopoda / голотип, 25.

**Current nomenclature**. *Uniramidesmus aberrans* Mikhaljova, 1979 (Sierwald et al. 2026).

**Remarks**. Only paratypes (4♂ 8♀) are in the ZMMU collection (A. Schileyko, pers. comm., 4.II.2026), even though the entire type series was stated in the original description (Golovatch and Mikhaljova 1979) to have been deposited in ZMMU.

***alveolatus* Mikhaljova in Golovatch & Mikhaljova, 1979 (*Uniramidesmus* )**

*Uniramidesmus alveolatus* Golovatch & Mikhaljova, 1979: 832, figs 1.5–9 (D♂♀).

**Type. *Holotype***: (♂), MYR_DIP_0000088.

**Type locality**. Russia, Maritime Territory, Ussuri State Res. (ca 43°39'N, 132°30'E).

**Label data**. *Uniramidesmus* / *alveolatus* Mikhaljova / Опр. {Det.} det. Е.В. Михалёва. {E.V. Mikhaljova} / Сбор {Collected} Приморский край / Уссурийский заповедник, / чернопихтарник, подстилка: {Maritime Territory, Ussuri Res., fir forest, litter} / 24.VI.1977 (leg. Г.Ф. Курчева и / Е.В. Михалёва). {G.F. Kurcheva and E.V. Mikhaljova} / голотип, / holotype, / 24 // Diplopoda, голотип {holotype}, 24 // Приморский край, / Уссурийский (бывший Супутинский) / заповедник, / чернопихтарник, / подстилка, 24.6.1977 {Maritime Territory, Ussuri (former Suputinsky) Res., fir forest, litter} / г. leg. Курчева Г.Ф., Ми – / халёва Е.В. {G.F. Kurcheva and E.V. Mikhaljova} / *Uniramidesmus alve*- / *olatus* Mikh. 1 ♂ / голотип. Holotypus // Примор. кр. Уссур. з-к, / чернопихтарник, п. п. № 2; / подст., 24.6.77. {Maritime Territory, Ussuri Res., fir forest, site № 2, litter} / Курчева Г.Ф., Михалёва Е.В {G.F. Kurcheva and E.V. Mikhaljova}.

**Current nomenclature**. *Uniramidesmus alveolatus* Mikhaljova, 1979 (Sierwald et al. 2026).

**Remarks**. Although the entire type series was stated in the original description to have been deposited in ZMMU, none are currently available there (A. Schileyko, pers. comm., 4.II.2026).

***assimilis* Lohmander, 1936 (*Brachydesmus* )**

*Brachydesmus assimilis* Lohmander, 1936: 12, fig. 2 (D♂).

**Types. *Syntypes***: (1♂), MYR_DIP_0000302; (1♂), MYR_DIP_0000303.

**Type locality**. Three localities in Georgia: Borjomi (ca 41°50'N, 43°23'E), Tbilisi (ca 41°42'N, 44°48'E) and Lagodechi (ca 41°49'N, 46°16'E). Since a lectotype has not been designated, the type locality cannot be unambiguously selected.

**Label data**. MYR_DIP_0000302: *Brachydesmus* / *assimilis* n.sp. / Hans Lohmander // Зоол. Муз. АН {Zoological Museum of the Academy of Sciences} / №64-1917 г. / 783. *Brachydesmus* / *assimilis* n.sp. / Опр. {Det.} H. Lohmander. / Сбор {Collected} 21.IV.1912 г. Тифл. / губ. Сигнахский у. {Tiflis Province, Sugnakhsky Distr.} / Лагодехи {Lagodekhi} / Л.А. Ланц. {L.A. Lants}.

MYR_DIP_0000303: *Brachydesmus* / *assimilis* n.sp. / Hans Lohmander // Зоол. Муз. АН {Zoological Museum of the Academy of Sciences} / №304-1915 г. / 784. *Brachydesmus* / *assimilis* n.sp. / Опр. {Det.} H. Lohmander. / Сбор {Collected} 17.IV.1906 г. / Тифлис {Tiflis} / К. Сатунин {K. Satunin} / (defect.!).

**Current nomenclature**. *Brachydesmus assimilis* Lohmander, 1936 (Sierwald et al. 2026).

**Remarks**. The remaining paratype (1♂) from Borjomi is deposited in GMNH (MYRIA000014635; O. Gran, pers. comm., 5.II.2026). The male in the ZISP (sample MYR_DIP_0000303) is fragmented, with some segments missing. Both syntypes dried out and were moistened on 13.IV.2026.

***dentatus* Mikhaljova in Golovatch & Mikhaljova, 1979 (*Uniramidesmus* )**

*Uniramidesmus dentatus* Golovatch & Mikhaljova, 1979: 833, figs 2.1–4 (D♂♀).

**Type. *Holotype***: (♂), MYR_DIP_0000090.

**Type locality**. Russia, Maritime Territory, Ussuri State Res. (ca 43°39'N, 132°30'E).

**Label data**. *Uniramidesmus* / *dentatus* Mikhaljova / Опр. {Det.} Е.В. Михалёва / Сбор {Collected} Приморский край, / Уссурийский заповедник, / чернопихтарник; подстилка: {Maritime Territory, Ussuri Res., fir forest, litter} / 10.VI.1977 (leg. Г.Ф. Курчева и / Е.В. Михалёва) {G.F. Kurcheva and E.V. Mikhaljova} / голотип / holotype / 26 // голотип 26 // Приморский край, / Уссурийский (быв / ший Супутинский) / заповедник, черно- / пихтарник, подстилка, {Maritime Territory, Ussuri (former Suputinsky) Res., fir forest, litter} / 10.6.1977 г.; leg. Кур- / чева Г.Ф., Михалёва Е.В. G.F. Kurcheva and E.V. Mikhaljova} / *Uniramidesmus dentatus* Mikh. / 1♂ голотип. Holotypus // Уссур. З-к {Ussury Res.}, П.П. №2 / раск. №3, Ао III; 10.6.77.

**Current nomenclature**. *Uniramidesmus dentatus* Mikhaljova, 1979 (Sierwald et al. 2026).

**Remarks**. Only paratypes (4♂ 11♀ 27 juv.) are in the ZMMU collection (A. Schileyko, pers. comm., 4.II.2026), even though the entire type series was stated in the original description (Golovatch and Mikhaljova 1979) to have been deposited in ZMMU.

***detersus* Golovatch in Golovatch & Mikhaljova, 1979 (*Uniramidesmus* )**

*Uniramidesmus detersus* Golovatch & Mikhaljova, 1979: 830, figs 1.1–4 (D♂♀).

**Type. *Holotype***: (♂), MYR_DIP_0000099.

**Type locality**. Russia, Vladivostok, Okeanskaya station (ca 43°14'07.2"N, 132°00'28.7"E).

**Label data**. *Uniramidesmus* / *detersus* Golovatch. / Опр. {Det.} С. Головач. / Сбор {Collected} Приморский край, / Владивосток, станция / Океанская, поваленный / лес, гнилой березовый пень: {Maritime Territory, Vladivostok, Okeanskaya Station, fallen forest, rotten birch stump} / 8.V.1977 (leg. Е.В. Михалёва) {E.V. Mikhaljova} / голотип, holotype, 27 // Diplopoda / голотип, № 27 // Приморский край, / oкраина г. Владивостока, / станция Океанская, / пойменный лес, гнилой / березовый пень {Maritime Territory, nr Vladivostok, Okeanskaya station, floodplain forest, rotten birch stump}, 8.5.1977 г. / leg. Михалёва Е.В. {E.V. Mikhaljova} / *Uniramidesmus detersus* / Gol. 1♂ голотип. / holotypus // Прим. кр., oкраина / г. Владивостока, / ст. «Океанская», / пойменн. лес, / гнил. берез. пень, {Maritime Territory, nr Vladivostok, Okeanskaya Station, floodplain forest, rotten birch stump} / 8.5.77.

**Current nomenclature**. *Uniramidesmus detersus* Golovatch, 1979 (Sierwald et al. 2026).

**Remarks**. Only paratypes (5♂ 3♀) are in the ZMMU collection, even though the entire type series was stated in the original description (Golovatch and Mikhaljova 1979) to have been deposited in ZMMU.

***dshungaricus* Golovatch, 1979 (*Schizoturanius* )**

*Schizoturanius dshungaricus* Golovatch, 1979a: 996, figs 2.7–9 (D♂♀).

**Types. *Holotype***: (♂), MYR_DIP_0000206. ***Paratypes***: (1♂), MYR_DIP_0000207; (1♀), MYR_DIP_0000208.

**Type locality**. Kazakhstan, coordinates are given for the modern-day Ranger Station Dzhalansh situated in the valley of Zhalanash River (ca 45°29'N, 80°31'E).

**Label data**. MYR_DIP_0000206: *Schizoturanius* / *dshungaricus* Gol. / 1♂ голотип / holotypus / + препарат {preparation} // голотип 23. {holotype} / Diplopoda // *Schizoturanius* / *dshungaricus* Golovatch / det. S. Golovatch, Головач / Восточный Казахстан. / Джунгарский Алатау, доли- / на рек Джаланаш и Теректы: {East Kazakhstan, Dzungarsky Alatau [Mt. Range], valley of Dzhalanash and Terekty Rivers} / 16–22.VIII.1936, В. Шнитников {[leg.] V. Shnitnikov} / Зоол. Инст. АН №232-1937 / голотип/ holotypus / 23 // Долина рек / Джаланаш и Теректы, / Джунгарский Алатау / 16–22.VIII.1936 / ЗИН №232-1937. / leg. В. Шнитников {V. Shnitnikov}.— Micropreparation: *Schizoturanius* / *dshungaricus* Golovatch / 1♂ голотип / holotypus 23 / гоноподии {gonopods} // Казахстан / Джунгарский Алатау / долина р. Джаланаш / и Теректы {Kazakhstan, Dzungarsky Alatau [Mt. Range], valley of Dzhalanash and Terekty Rivers} / 16–22.VIII.1936 / leg. В.Н. Шнитников {V.N. Shnitnikov} / (колл. ЗИН №232-1937) // 0000206.

MYR_DIP_0000207: The body of paratype male is absent. – Micropreparation: *Schizoturanius* / *dshungaricus* Golovatch / 1♂ паратип / paratypus 23 / гоноподии {gonopods} // Казахстан 207 / Джунгарский Алатау / долина р. Джаланаш {Kazakhstan, Dzungarsky Alatau [Mt. Range], valley of Dzhalanash River} / 16–17.VIII.1936 / leg. В.Н. Шнитников / (колл. ЗИН №232-1937) // 0000207.

MYR_DIP_0000208: The body of the paratype female is absent. – Micropreparation: *Schizoturanius* / *dshungaricus* Golovatch / 1♀, паратип {paratype) / вульвы {vulvae} // Казахстан 208 / Джунгарский Алатау / долина р. Джаланаш / и Теректы {Kazakhstan, Dzungarsky Alatau [Mt. Range], valley of Dzhalanash and Terekty Rivers} / 16–22.VIII.1936 / leg. В.Н. Шнитников / (колл. ЗИН №232-1937) // 0000208.

**Current nomenclature**. *Schizoturanius dshungaricus* Golovatch, 1979 (Sierwald et al. 2026).

**Remarks**. The torsos of both paratypes are missing, only their slides are available.

***expressus* Golovatch, 1979 (*Turanodesmus* )**

*Turanodesmus expressus* Golovatch, 1979a: 991, figs 2.1–6 (D♂♀).

**Types. *Holotype***: (♂), MYR_DIP_0000115. ***Paratype***: (1♀), MYR_DIP_0000114.

**Type locality**. Kazakhstan, Almaty Oblast, Talgar Distr., Bolshaya Almatinka River (ca 43°08'N, 76°54'E).

**Label data**. MYR_DIP_0000115: голотип / № 22 / Diplopoda // *Turanodesmus* / *expressus* Gol. / 1♂ голотип. / holotypus / + препарат // Б. Алмаатинка, / IX.11.33 / В. Шнитников // № 5 ~ 1936.— Micropreparation: *Turanodesmus* / *expressus* Golovatch / 1♂ голотип / holotypus / гоноподии {gonopods} / 115 // Казахстан / Джунгарский Алатау / р. Большая Алмаатинка {Kazakhstan, Dzhungarian Alatau [Mt. Range], Bol’shaya Almaatinka River} / IX 1933, leg. В.Н. Шнитников {V.N. Shnitnikov} / (колл. ЗИН №5-1936) {coll. ZIN}.

MYR_DIP_0000114: *Turanodesmus* / *expressus* Gol. / 1♀ паратип / paratypus // Б. Алмаатинка {Bolshaya Almaatinka [River]} / 11.IX.1933 / 1♀ + препарат // №5–1936 г. // 15.— Micropreparation: *Turanodesmus* sp. / 1♀ / det. С. Головач {S. Golovatch} – 114 // Казахстан / Джунгарский Алатау / р. Большая Алмаатинка {Kazakhstan, Dzhungarian Alatau [Mt. Range], Bol’shaya Almaatinka River} / у озера {nr lake}, 29.VIII.1933 / leg. В.Н. Шнитников {V.N. Shnitnikov} / (колл. ЗИН №5-1936).

**Current nomenclature**. *Jaxartes expressus* (Golovatch, 1979) (Sierwald et al. 2026). Transferred to the genus *Jaxartes* Verhoeff, 1930 by Antić et al. (2019).

**Remarks**. This species was described from two specimens: the holotype ♂ and a ♀ paratype.

***ferrugineus* Lohmander, 1936 (*Brachydesmus* )**

*Brachydesmus ferrugineus* Lohmander, 1936: 22, figs 7, 8 (D♂♀).

**Types. *Syntypes***: (1♂), MYR_DIP_0000247; (2♂), MYR_DIP_0000248; (1♂), MYR_DIP_0000270.

**Type locality**. Abkhazia, Akhali Atoni (ca 43°05'N, 40°49'E).

**Label data**. MYR_DIP_0000247: Зоол. Муз. АН {Zoological Museum of the Academy of Sciences} / №64-1917 г. / 779. *Brachydesmus* / *ferrugineus* n.sp. / Опр. {Det.} H. Lohmander / Сбор {Collected} 18.V.1912 г. Черно- / морская губ. Новый / Афон {Chernomorskaya Province, Novy Afon}, В. Линдгольм {V. Lindholm [col.]} // lectotype ! / S. Golovatch, 1990, / design[ated] // *Brachydesmus* / *ferrugineus* n.sp. / Hans Lohmander.

MYR_DIP_0000248: Зоол. Муз. АН {Zoological Museum of the Academy of Sciences} / №64-1917 г. / 778. *Brachydesmus* / *ferrugineus* Lohm / Опр. {Det.} Typen. / Сбор {Collected} 20.V.1912 г. Запад. / Закавказье, Ольгин- / ское, Цебельда. {West Transcaucasia, Ol’ginskoe, Tsebel’da} / В. Линдгольм {V. Lindholm [col.]} // *Brachydesmus* / *ferrugineus* n.sp. / Typen Hans Lohmander.

MYR_DIP_0000270: Зоол. Муз. АН {Zoological Museum of the Academy of Sciences} / №183-1909 г. / 731. *Brachydesmus* / *ferrugineus* Lohm / Опр. {Det.} Spec. orig.? / Сбор {Collected} Тифлисск. губ. {Tiflis Province} / ущ. Банис-хеви-цха- / ли у Боржома {Bani-khevi-tskhali [river] canyon nr Borzhomi} / 13.VI.1909 г. Л. Берг {L. Berg} // *Brachydesmus* / *ferrugineus* n.sp. / Hans Lohmander.

**Current nomenclature**. *Brachydesmus kalischewskyi* Lignau, 1915 (Sierwald et al. 2026). Synonymised with *B. kalischewskyi* by Golovatch et al. (2016). The enclosed label states that the lectotype ♂ (MYR_DIP_0000247) was designated by SIG in 1990, but in fact while synonymising this name with *B. kalischewskyi*, Golovatch et al. (2016: 27–28) referred to the ZISP specimens as syntypes. The type locality for *B. ferrugineus* was chosen based on this unpublished designation of the lectotype.

**Remarks**. The remaining syntypes (3♂ 7♀) are kept in FMNH. All ZISP specimens are broken in two or three pieces.

***faustus* Golovatch, 1987 (*Himalodesmus* )**

*Himalodesmus faustus* Golovatch, 1987: 213, figs 23–28 (D♂♀).

**Types. *Paratypes***: (1♂ 1♀), MYR_DIP_0000134.

**Type locality**. Nepal, between Jagat (Gorkha Distr.; ca 28°18'N, 84°51'E) and Pangshing (nr Deng; ca 28°28'N, 84°51'E).

**Label data**. *Himalodesmus faustus* / Golovatch, 1986 / Опр. {Det.} С. Головач. / Сбор {Collected} J. Martens & / W. Schawaller; Nepal / 1 ♂ 1 ♀. паратипы. paratypes // *Himalodesmus faustus* / Golovatch, n. sp. / 1 ♂ 1 ♀ paratypes. / паратипы. / det. S. Golovatch, 1984 // Nepal, Gorkha / Dhading distr., / Buri Gandaki, Jagat to above / Pangshing, 1650 m cultured / land 31.VII.1983. J. MARTENS / & W. SCHAWALLER № 223.

**Current nomenclature**. *Himalodesmus faustus* Golovatch, 1987 (Sierwald et al. 2026).

**Remarks**. The holotype ♂ and remaining paratypes (3♂ 12♀) are in SMF.

***inermis* Lohmander, 1933 (*Turanodesmus* )**

*Turanodesmus inermis* Lohmander, 1933: 5, figs 1–4, 7 (D♂♀).

**Types. *Syntypes***: (4♀), MYR_DIP_0000253; (1♂ 3♀), MYR_DIP_0000254.

**Type locality**. Uzbekistan, Sardaba (ca 40°32'21"N, 68°24'40"E).

**Label data**. MYR_DIP_0000253: Зоол. Муз. АН {Zoological Museum of the Academy of Sciences} / №267-08 г. / 610. *Turanodesmus* / (*Kirgisdesmus*) *inermis* / H. Lohm. / Опр. {Det.} H. Lohmander / Сбор {Collected} Туркестан, ст. Го- / лодная степь, уроч. / Сардаба. {Turkestan, Golodnaya steppe station, Sardaba tract} 19.II.1908 г. / Н. Зарудный. {N. Zarudny} // *Turanodesmus* / *inermis* n.sp. / Hans Lohmander.

MYR_DIP_0000254: Зоол. Муз. АН {Zoological Museum of the Academy of Sciences} / №347-1909 г. / 609. *Turanodesmus* / (*Kirgisdesmus*) *inermis* / H. Lohm. / Опр. {Det.} spec. orig. ! 1♂2♀ / Сбор {Collected} Туркестан, ст. Голод- / ная степь, уроч. Сардаба. {Turkestan, Golodnaya steppe station, Sardaba tract} 25.III.1909 г. / Н. Зарудный. {N. Zarudny} // *Turanodesmus* / *inermis* n.sp. / Hans Lohmander.

**Current nomenclature**. *Jaxartes inermis* (Lohmander, 1933) (Sierwald et al. 2026). Transferred to the genus *Jaxartes* Verhoeff, 1930 by Antić et al. (2019).

**Remarks**. All specimens are fragmented and damaged, some segments and legs missing. Based on the original description (Lohmander 1933: 9), the sample MYR_DIP_0000253 should also have contained 1♂ which is now in GMNH (MYRIA000014768; O. Gran, pers. comm., 26.I.2026). Although the label of the sample MYR_DIP_0000254 states that 1♂ 2♀ were enclosed (in agreement with Lohmander 1933), there are actually 3♀ inside.

***kalischewskyi* Lignau, 1915 (*Brachydesmus* )**

**Figure 4.**
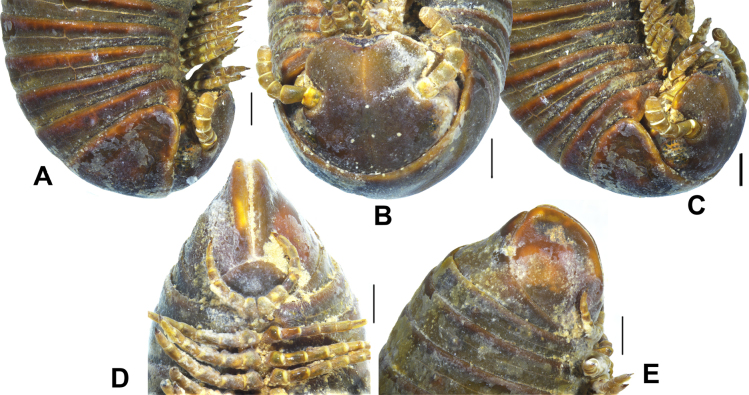
*Anadenobolus grandis* (Brandt, 1840), comb. nov. (= Julus (Spirobolus) grandis Brandt, 1840), ♀ lectotype. **A–C**. Anterior part of body, lateral, ventral and sublateral views, respectively; **D, E**. Posterior part of body, ventral and lateral views, respectively. Scale bar: 2 mm. Photographed by DVL.

**Figure 5.**
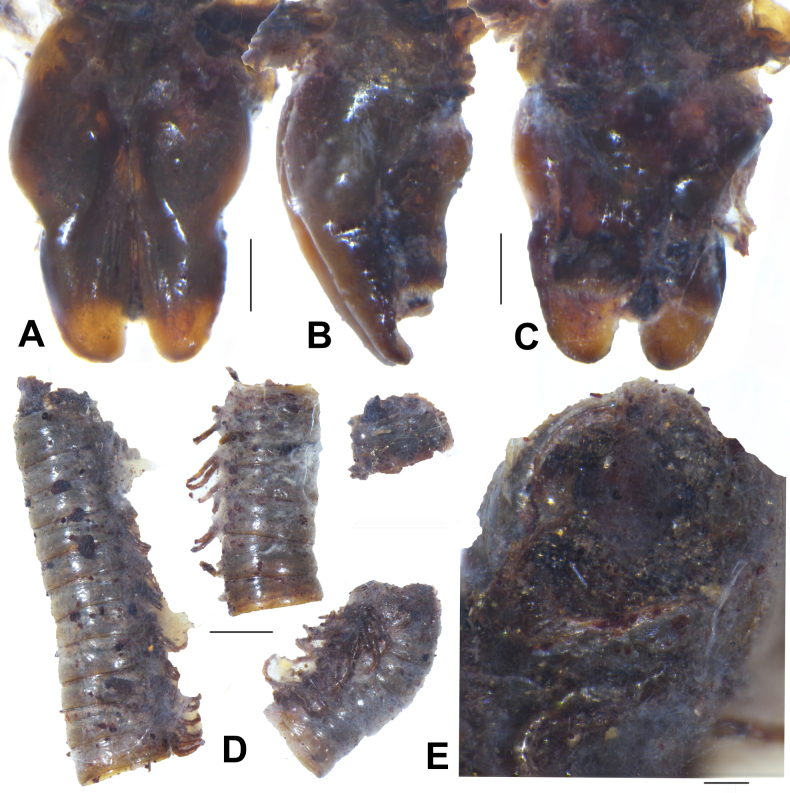
*Iulomorpha rotundata* (Brandt, 1840), comb. nov. (= Julus (Spirostreptus) rotundatus Brandt, 1840), ♂ holotype. **A–C**. Gonopods, anterior, lateral, and posterior views, respectively; **D**. Body fragments; **E**. Damaged head section. Scale bars: 0.2 mm (**A–C, E**); 1 mm (**D**). Photographed by DVL.

*Brachydesmus kalischewskyi* Lignau, 1915: 370, tab 4, figs 3–5 (D♂♀).

**Types. *Syntypes***: (1♂ 3♀), MYR_DIP_0000319.

**Type locality**. Abkhazia. Lignau (1915: 371) reported four specimens collected from four localities in Abkhazia, all of which are now kept in the same sample. Therefore, the type locality cannot be unambiguously selected.

**Label data**. *Brachydesmus kalischewskyi* sp.n. / опр. {det.} Лигнау {Lignau} / [on the reverse side] Abchasia, 30/VIII,05 / эксп. Камышевского {Kamyshevsky’s expedition} // 32. // Зоол. Инст. АН {Zool[ogical] Insti[tute] of the Academy of Sciences} / №162-1905 г. / 32. *Brychydesmus* / *kalischewskyi* Lign. / Опр. {Det.} Н. Лигнау {N. Lignau} / Сбор. {Col.} Абхазия {Abkhazia}, 1905 г. / М. Камышевский {M. Kamyshevsky}.

**Current nomenclature**. *Brachydesmus kalischewskyi* Lignau, 1915 (Sierwald et al. 2026).

**Remarks**. All syntypes except the male are fragmented.

***minor* Lignau, 1915 (*Polydesmus* )**

*Polydesmus minor* Lignau, 1915: 374, tab 4, fig. 3, text figs 9–11 (D♂♀).

**Types. *Syntypes***: (1♀), MYR_DIP_0000217; (1♀), MYR_DIP_0000218; (5♀), MYR_DIP_0000219; (1♂ 2♀), MYR_DIP_0000220; (3♂ 1♀ 1 juv.), MYR_DIP_0000221; (1♀), MYR_DIP_0000317.

**Type locality**. Abkhazia; since no lectotype has been designated, the type locality cannot be unambiguously selected.

**Label data**. MYR_DIP_0000217: *Polydesmus minor* n.sp. / опр. {det.} Лигнау {Lignau} // Зоол. Инст. АН {Zool[ogical] Insti[tute] of the Academy of Sciences} / №162-1905 г. / 425. *Polydesmus* / *minor* Lign. / Опр. {Det.} sp. orig. / Сбор. {Col.} Сухумский окр. {Sukhumi Distr.}, / в 2х вер. к зап. от сел. {two versts W of} / Ажары {Azhary Vil.}, 28.VII.1905 г. / М. Камышевский {M. Kamyshevsky}.

MYR_DIP_0000218: *Polydesmus minor* n.sp. / опр. {det.} Лигнау {Lignau} // Зоол. Инст. АН {Zool[ogical] Insti[tute] of the Academy of Sciences} / №162-05 г. / 422. *Polydesmus* / *minor* Lign. / Опр. {Det.} sp. orig. / Сбор. {Col.} Сухумский окр. {Sukhumi Distr.}; / прав. бер. р. Чхалты {right bank of Chkhalty River} / (Аугары), выс. 1130 м, зон. пихт. леса {(Augary), elevation 1130 m, zone of spruce forest} / 5.VIII.1905 г. / М. Камышевский {M. Kamyshevsky} // Сухумский округ {Sukhumi Distr.} / правый берег р. {right bank of river} / Чхалты (Аугары) {Chkhalty (Augary)} / зона пихтового леса (zone of spruce forest} / высота (elevation} / над уровнем моря (above sea level} / 1130 метров (meters) / 19.VIII.05 / эксп. Камышевского {Kamyshevsky’s expedition}.

MYR_DIP_0000219: *Polydesmus minor* n.sp. / опр. {det.} Лигнау {Lignau} / [on the reverse side] С. Ажары {Azhary Vil.}, 30/VIII,05 / эксп. Камышевского {Kamyshevsky’s expedition} // Зоол. Инст. АН {Zool[ogical] Insti[tute] of the Academy of Sciences} / №162-1905 г. / 424. *Polydesmus* / *minor* Lign. / Опр. {Det.} sp. orig. / Сбор. {Col.} Сухумский окр. {Sukhumi Distr.} / c. Ажары {Azhary vil.}, 30.VIII.05 г. / М. Камышевский {M. Kamyshevsky}.

MYR_DIP_0000220: *Polydesmus minor* n.sp. / опр. {det.} Лигнау {Lignau} / [on the reverse side] близ впадения р. Чхалты / в Кодор, экс. Камы- / шевского {nr confluence of Chkhalty and Kodor rivers, Kamyshevsky’s expedition}, 1.VIII.05 г. // Зоол. Инст. АН {Zool[ogical] Insti[tute] of the Academy of Sciences} / №162-05 г. / 424. *Polydesmus* / *minor* Lign. / Опр. {Det.} sp. orig. / Сбор. {Col.} Сухумский окр. {Sukhumi Distr.}; / бл. впад. р. Чхалты / в Кодор {nr confluence of Chkhalty and Kodor rivers}, 1.VIII.1905 г. / М. Камышевский {M. Kamyshevsky}.

MYR_DIP_0000221: *Polydesmus minor* n.sp. / опр. {det.} Лигнау {Lignau} \ [on the reverse side] Верхняя Цебельда {Verkhnyaya Tsebel’da} / 21/VIII.05 / эксп. Камышевского {Kamyshevsky’s expedition} // Зоол. Инст. АН {Zool[ogical] Insti[tute] of the Academy of Sciences} / №162-05 г. / 423. *Polydesmus* / *minor* Lign. / Опр. {Det.} sp. orig. / Сбор. {Col.} Сухумский окр. {Sukhumi Distr.} / Верх. Цебельда {Verkhnyaya Tsebel’da} / 21.VIII.1905 г. / М. Камышевский {M. Kamyshevsky} // 2 экз. взяты {two specimens taken by} / Dr. Schubert.

MYR_DIP_0000317: *Polydesmus minor* n.sp. / опр. {det.} Лигнау {Lignau} / [on the reverse side] С. Ажары. Абхазия {Azhary Vil., Abkhasia} / эксп. Камышевского {Kamyshevsky’s expedition}, 28/VII.05 // Зоол. Инст. АН {Zool[ogical] Insti[tute] of the Academy of Sciences} / №162-1905 г. / 426. *Polydesmus* / *minor* Lign. / Опр. {Det.} sp. orig. / Сбор. {Col.} Сухумский окр. {Sukhumi Distr.} / c. Ажары {Azhary Vil.}, 28.VII / 1905 г. / М. Камышевский {M. Kamyshevsky}.

**Current nomenclature**. *Polydesmus lignaui* Lohmander, 1936 (Golovatch et al. 2016; Sierwald et al. 2026). Lohmander (1936: 11) proposed *Polydesmus lignaui* as a replacement name for *P. minor* Lignau, 1915, non *P. minor* Bollman, 1888.

**Remarks**. Based on the original description (Lignau 1915: 374), the type series consisted of 55 specimens (syntypes), of which only 16 are available in the ZISP collection (4♂ 11♀ 1 juv.). Two syntypes (1♂ 1♀) are available in ZMB (Moritz and Fischer 1978). The whereabouts of the remaining syntypes are unknown. Except for the samples MYR_DIP_0000217 and MYR_DIP_0000317, all ZISP material is represented by broken and fragmented specimens. The sample MYR_DIP_0000317 dried out and was moistened on 15.IV.2026.

***montivagus* Lohmander, 1933 (*Schizoturanius* )**

*Schizoturanius montivagus* Lohmander, 1933: 12, fig. 10 (D♂).

**Type. *Holotype***: (♂), MYR_DIP_0000273.

**Type locality**. Kyrgyzstan, Chuy Region, Chon-Uchimchek stream (ca 42°9'33"N, 73°57'45"E).

**Label data**. MYR_DIP_0000273: Зоол. Муз. АН {Zoological Museum of the Academy of Sciences} / №366-13 г. / 612. *Schizoturanius* / *montivagus* Lohm / Опр. {Det.} Spec. orig. / Сбор {Collected} №312. Семиречье {Semirechie} / Тишненск. у., река {Tishnensky Province, river} / Учимчик {[Chon-]Uchimchek}, 11.VIII.1913 г. / В. Чернавин {V. Chernavin} // *Schizoturanius* / *montivagus* / Lohmander, 1933 / ♂ holotype / revised by Golovatch / 2022 // *Sibiriodesmus* / *montivagus* n.sp. / Hans Lohmander.

**Current nomenclature**. *Schizoturanius montivagus* Lohmander, 1933 (Sierwald et al. 2026).

**Remarks**. This species was described from the holotype ♂ alone. The holotype is fragmented.

***muralewiczi* Lohmander, 1936 (*Polydesmus* )**

*Polydesmus muralewiczi* Lohmander, 1936: 9, fig. 1 (D♂).

**Types. *Lectotype***: (♂), MYR_DIP_0000266. ***Paralectotype***: (1♀), same number.

**Type locality**. Russia, Krasnodar Territory, Psebai Vil. (ca 44°08'N, 40°49'E).

**Label data**. Зоол. Муз. АН {Zoological Museum of the Academy of Sciences} / №285-1911 г. / 785. *Polydesmus* / *muralewiczi* Lohm. / Опр. {Det.} Typen. / Сбор {Collected} 20.V.1911 г. Кубанск. / обл. ст. Псебай {Kubanskaya Oblast, Psebai Vil.}; под / камнями на под’еле / высот Герисгель. {under stones, at the elevation of Gerisgel’} / Волнухин {[D.] Volnukhin [col.]} 1♂ lectotype, / 1♀ paralectotype / designated by / S. Golovatch, 2015 // *Polydesmus* / *muralewiczi* n.sp. / Typen Hans Lohmander.

**Current nomenclature**. *Polydesmus muralewiczi* Lohmander, 1936 (Sierwald et al. 2026). The lectotype ♂ was designated by Golovatch et al. (2016).

**Remarks**. Although Lohmander (1936: 10) described the new species based on a single ♂, the type series in fact contains a female as well. Thus, both specimens were to be considered syntypes, and hence the lectotype ♂ was designated to avoid ambiguity (Golovatch et al. 2016). The lectotype is broken in two pieces. Lohmander (1936) provided, apparently by mistake, a different collecting date, 1.VI.1911, while the actual label stated 20.V.1911.

***occultus* Golovatch, 1986 (*Usbekodesmus* )**

*Usbekodesmus occultus* Golovatch, 1986a: 348, figs 3–8 (D♂♀).

**Types. *Paratypes***: (1♂ 1♀), MYR_DIP_0000118.

**Type locality**. Nepal, Kali Gandaki Valley (main gorge; ca 28°42'N, 83°38'E).

**Label data**. *Usbekodesmus occultus* / Golovatch, 1986 / С. Головач {S. Golovatch} / Сбор {Collected} J. Martens / Nepal / 1♂ 1♀ паратипы {paratypes} // *Usbekodesmus occultus* / Golovatch, n. sp. / 1♂ 1♀. паратипы, / paratypes. / det. С. Головач, 1983. / взято из Зенкенбергского / музея за обработку {retained from Senckenberg Museum for help with ID} // Nepal: Thakkhola: Kali / Gandaki Valley between Annapurna / & Dhaulagiri Himal, Chadziou / Khola Valley, 2700–2800 m, / bamboo growth, October, 1969 / leg. J. MARTENS, № 39.

**Current nomenclature**. *Epanerchodus occultus* (Golovatch, 1986) (Sierwald et al. 2026). Transferred to the genus *Epanerchodus* Attems, 1901 by Golovatch et al. (2011).

**Remarks**. The holotype ♂ and the remaining paratypes (4♂ 4♀) are in SMF.

***parvus* Golovatch, 1987 (*Himalodesmus* )**

*Himalodesmus parvus* Golovatch, 1987: 212, figs 23, 24 (D♂♀).

**Types. *Paratypes***: (1♂ 1♀), MYR_DIP_0000109.

**Type locality**. Nepal, Panchthar Distr., approximate coordinates are given for Paniporua Mt Ridge (ca 27°06'N, 87°55'E).

**Label data**. *Himalodesmus parvus* / Golovatch, 1986 / Опр. {Det.} С. Головач. / Сбор {Collected} J. Martens & B. Daams. / Nepal / 1♂ 1♀ паратипы / paratypes // *Himalodesmus parvus* / Golovatch, n. sp. / 1♂ 1♀ паратипы, paratypes / Оставлен за обработку {Retained for help with ID} / det. С. Головач {S. Golovatch}, 1984 // Nepal, Panchthar distr., / Grat, between Sheldoti / and Paniporua, 2200 m, / broad-leaved forest, 29.VIII.1983. / leg. J. MARTENS & B. DAAMS № 259.

**Current nomenclature**. *Himalodesmus parvus* Golovatch, 1987 (Sierwald et al. 2026).

**Remarks**. The holotype ♂ and remaining paratypes (2♂ 2♀) are in SMF.

***potanini* Golovatch, 1991 (*Epanerchodus* )**

*Epanerchodus potanini* Golovatch, 1991: 153, figs 1, 2, 19–22 (D♂♀).

**Types. *Holotype***: (♂), MYR_DIP_0000152. ***Paratypes***: (4♀ 3 juv.), MYR_DIP_0000147; (2♀), MYR_DIP_0000148; (3♀), MYR_DIP_0000149; (1♂ incomplete 1♀ incomplete 1 fragm.), MYR_DIP_0000150; (1♂), MYR_DIP_0000151; (1♀), MYR_DIP_0000153.

**Type locality**. China, Sichuan, Kangding (=Da-tsien-lu or Tachienlu) (ca 30°03'N, 101°53'E).

**Label data**. MYR_DIP_0000152: // *Epanerchodus potanini* / Golovatch. n. sp. / 1♂ holotype. / голотип! / det. С. Головач, 1991. / S. Golovatch // China, Szechuan Prov., / Da-tsien-lu (now Kangding), / ~ 2500 m, 14.VII.1983, leg. / G.N. Potanin // Тацзинлу / (Сычуань). / 14-VII-1893. / Китай цент // Potanin. 14VII.1983. / Da-tsien-lu // Cat. № 19. / Spem. Био // Holotypus ♂.

MYR_DIP_0000147: *Epanerchodus potanini* / Golovatch, n. sp./ 4♀, 3 juv.; paratypes. / det. С. Головач, 1991 // China, Szechuan Prov., / Da-tsien-lu (now Kangding), / ~ 2500 m, 12.05.1893; G. Potanin // Тацзинлу / (Китай Цент.) / Потанинъ {Da-tsien-lu, central China, Potakin} // Potanin. 12. V.1893 (n. st.) / b. vic. nrb. Dn-tzsjan-lu // Cat. № 9; Spem. 7 (septem).

MYR_DIP_0000148: *Epanerchodus potanini* / Golovatch, n. sp./ 2♀ paratypes – паратипы. / det. С. Головач, 1991 // China, Szechuan Prov., / Da-tsien-lu (now Kangding), / ~ 2500 m, 30.V.1893; G. Potanin leg // Тацзинлу / (Сычуань). {Da-tsien-lu, Szechuan} / 30-V-1893. / Потанинъ. Центр. Китай {Potanin, central China}// Potanin. 30.V.1893 / S. vic. rrb. Dn-tzsjan- / lu (Sy-Tseluneum // Cat. № 4; / spec. 2.

MYR_DIP_0000149: *Epanerchodus potanini* / Golovatch, n. sp. / 3♀ paratypes ~ паратипы. / det. С. Головач, 1991 // China, Szechuan Prov., / Da-tsien-lu (now Kangding), / 12.V.1893; leg. G.N. POTANIN. / Zool. Mus. Leningrad // Центр. Китай {central China} // 17.VI.1893. Potanin. / Da-tsien-lu // Cat. № 15; Spm. 3.

MYR_DIP_0000150: *Epanerchodus potanini* / Golovatch, n. sp. / 1♂ incomplete ?; 1♀ (incomplete ?); / 1 fragm. паратипы. {paratypes} / det. С. Головач, 1991. / S. Golovatch // China, Kansu Prov., pass betw. / Villages Morping & Wuping, / ~ 2700 m a. s. p., ~ 104,5°E & / 33,5°N, 4.VII.1885; G. Potanin leg // Китай, перевал (~ 2700 м н. у. м.) / между деревнями Морпин и / Упин, ~ 104,5°E и 33,5°N, {China, pass between Morping and Wuping} / юго-вост. угол пров. Таньсу {SE corner of Kansu Prov.} / 4.VII.1885, leg. Г.Н. Потанин {G.N. Potanin} // Потанинъ {Potanin}.

MYR_DIP_0000151: *Epanerchodus potanini* / Golovatch, n. sp. / 1♂ паратип – paratype! / det. С. Головач, 1991 / S. Golovatch // China, Szechuan Prov., / Da-tsien-lu, 14.VII.1893; / leg. G. Potanin // препарат! {preparation}.

MYR_DIP_0000153: *Epanerchodus potanini* / Golovatch, n. sp. / ♀ паратип ~ paratype / det. С. Головач, 1991{S. Golovatch} // China, Kansu Prov., / forest betw. village Wuping / & river Popho, Mt. Cha- / gola, 10.VII.1985, Potanin leg. // Китай, пров. Таньсу, бор / между дер. Упин и рекой / Попхо (г. Чагола) {China, Kansu, forest between Wuping Vil. and Popho River, Chagola Mt.} / 10.VII.1985, Г. Потанин {G. Potanin} // Потанин {Potanin}.

**Current nomenclature**. *Epanerchodus potanini* Golovatch, 1991 (Sierwald et al. 2026).

**Remarks**. Some paratypes are in MHNG (1♀), SMF (1♀), ZMMU (1♀), and NHMD (1♀). A micropreparation for the sample MYR_DIP_0000151 (paratype ♂) is missing.

***polymorphus* Mikhaljova & Golovatch, 1981 (*Epanerchodus* )**

*Epanerchodus polymorphus* Mikhaljova & Golovatch, 1981: 1183, fig. 1.А–И (D♂♀).

**Types. *Paratypes***: (116♂ 59♀ 110 juv.), MYR_DIP_0000089.

**Type locality**. Russia, Maritime Territory, Lazovsky Res. (ca 42°57'N, 133°44'E).

**Label data**. Paratypes 116 ♂ 59 ♀, 110 juv: *Epanerchodus* / *polymorphus* Mikh et Gol. / Опр. {Det.} Mikh. et Gol. / Сбор {Collected}. Приморский кр. {Maritime Territory} / 12.V.1900 / leg. П.Ю. Шмидт {P.Yu. Schmidt} / 116♂ 59♀, 110 juv / паратипы {paratypes} // Приморский край, / Партизанский район, / залив Находка, у бухты / Козьминой и озера / Солёного, между камнями, {Maritime Teritory, Partizansky Distr., Nakhodka Bay, by the bay Koz’mina and Solenoe Lake, among stones} / 12.5.1900 г., / leg. П.Ю. Шмидт {P.Yu. Schmidt} / *Epanerchodus polymorphus* / Mikh. et Gol. / 116 ♂ 59 ♀, 110 juv. / паратипы. Paratypes // № 53. 12.V.1900 / Окр. Владивостока / у бух. Козьминой / и оз. Соленого: между / камнями / П. Шмидт {Vladivostok vicinities, by the bay Koz’mina and Solenoe Lake, among stones, P. Schmidt [col.]} / № 250–1902 г. // 61 // № 930 // MYR_DIP_0000089.

**Current nomenclature**. *Epanerchodus polymorphus*Mikhaljova and Golovatch 1981 (Sierwald et al. 2026).

**Remarks**. The holotype ♂ is in ZMMU. Of the paratypes (223♂ 110♀ 5 juv.), which were supposed to be placed in ZMMU (see Mikhaljova and Golovatch 1981), only the following ones (77♂ 35♀ 4 juv.) are currently kept there (A. Schileyko, pers. comm., 4.II.2026); the whereabouts of the remaining paratypes remain unknown.

***redikorzevi* Lohmander, 1933 (*Usbekodesmus* )**

*Usbekodesmus redikorzevi* Lohmander, 1933: 17, figs 13–15 (D♂♀).

**Types. *Syntypes***: (1♂ 9♀), MYR_DIP_0000245; (1♂ 3♀), MYR_DIP_0000246.

**Type locality**. Uzbekistan, two localities: nr Samarkand (ca 39°36'N, 66°58'E) and Guzar (= G'uzor; ca 38°36'N, 66°15'E). Since a lectotype has not been designated, the type locality cannot be unambiguously selected.

**Label data**. MYR_DIP_0000245: Зоол. Муз. АН {Zoological Museum of the Academy of Sciences} / №45-96 г. / 616. *Usbekodesmus* / *redikorzevi* Lohm. / Опр. {Det.} spec. orig.! / Сбор {Collected} окр. Самарканда. {vicinity of Samarkand} / II.1896 г. / Барщевский [Barschevsky [col.]} // окр. Самарканда {vicinity of Samarkand} / 1896 [an illegible word] / Барщевский [Barschevsky [col.]} / [on the opposite side of the label]: №45-96 // *Polydesmus* / *redikorzevi* n.sp. / Hans Lohmander.

MYR_DIP_0000246: Зоол. Муз. АН {Zoological Museum of the Academy of Sciences} / №90-97 г. / 615. *Usbekodesmus* / *redikorzevi* Lohm. / Опр. {Det.} spec. orig.! 1♂3♀ / Сбор {Collected} Бухара, Гузар {Bukhara, Guzar} / 1897 г. / А. Казнаков {A. Kaznakov [col.]} // Гузаръ {Guzar} / А. Казнаков {A. Kaznakov [col.]} / [on the opposite side of the label]: №90-97 // *Polydesmus* / *redikorzevi* n.sp. / Hans Lohmander.

**Current nomenclature**. *Epanerchodus redikorzevi* (Lohmander, 1933) (Sierwald et al. 2026). Transferred to the genus *Epanerchodus* Attems, 1901 by Golovatch et al. (2011).

**Remarks**. Specimens in both samples are fragmented and badly damaged, with many legs missing. According to Lohmander (1933: 20), the sample MYR_DIP_0000245 should contain 12♀, but only nine are available. Two syntypes of unspecified sex (torsos and eight micropreparations) are in GMNH (MYRIA000014770–72; O. Gran, pers. comm., 26.I.2026).

***salairicus* Gulička, 1963 (*Turanodesmus* )**

*Turanodesmus salairicus* Gulička, 1963: 523, figs 2.3–4 (D♂♀).

**Type. *Holotype***: (♂), MYR_DIP_0000058.

**Type locality**. Russia, Kemerovo Oblast, Prokopyevsk (ca 53°52'N, 86°38'E).

**Label data**. *Turanodesmus salairicus* / Gulička / Опр. {Det.} Holotypus ♂ / Сбор {Collected} Ю.Б. Бызовой и З.В. / Чадаевой {Yu.B. Byzova and Z.V. Chadaeva} / Кемеров. обл. Прокопьевск / гидроузел, лес. Салаир- / ский кряж V–VI.960 {Kemerovo Oblast, Prokopievsk, waterworks, forest, Salair Mt. Range} // Кемеровская обл., / Прокопьевск, гидро- / узел, Салаирский кряж, / лес {Kemerovo Oblast, Prokopievsk, waterworks, Salair Mt. Range}; V–VI.1960 / ♂ голотип {holotype} / leg. Ю.Б. Бызова и З.В. Чадаева {Yu.B. Byzova and Z.V. Chadaeva} // *Turanodesmus salairicus* n. sp. Prokopievsk – Holotypus // № 918.

**Current nomenclature**. *Schizoturanius tabescens* (Stuxberg, 1876) (Sierwald et al. 2026). Synonymised with *S. tabescens* by Mikhaljova and Marusik (2004).

**Remarks**. The holotype is broken into two parts. The paratypes (6♂ 10♀) are kept embezzled in the SMNPS collection.

***sacer* Golovatch, 1987 (*Usbekodesmus* )**

*Usbekodesmus sacer* Golovatch, 1987: 208, figs 12–15 (D♂♀).

**Types. *Paratypes***: (2♂), MYR_DIP_0000116.

**Type locality**. Nepal, Gorkha Distr. (ca 28°17'N, 84°41'E), Chuling Khola R. valley, Meme Kharka (not found locality).

**Label data**. *Usbekodesmus sacer* / Golovatch, 1986. / С. Головач {S. Golovatch} / Сбор {Collected} J. Martens + / W. Schawaller / Nepal / 2♂ паратипы {paratypes} // *Usbekodesmus sacer* / Golovatch, n. sp. / 2 паратипа ♂ / paratypes / det. С. Головач, 1984 {S. Golovatch} // Nepal, Gorkha distr., Chuling / Khola, Meme Kharka, 3300–/ 3400 m, Wald/Moräne, 5.–6.VIII. / 1983, leg. J. MARTENS & W. SCHAWALLER. № 233.

**Current nomenclature**. *Epanerchodus sacer* (Golovatch, 1987) (Sierwald et al. 2026). Transferred to the genus *Epanerchodus* Attems, 1901 by Golovatch et al. (2011).

**Remarks**. The holotype ♂ and remaining paratypes (14♂ 5♀ 5 juv.) are in SMF. The ZISP paratypes are broken.

***talyschanus* Lohmander, 1936 (*Brachydesmus* )**

*Brachydesmus talyschanus* Lohmander, 1936: 26, figs 9, 10 (D♂).

**Type. *Holotype***: (♂), MYR_DIP_0000265.

**Type locality**. Azerbaijan, Lerik (ca 38°46'N, 48°25'E).

**Label data**. Зоол. Муз. АН {Zoological Museum of the Academy of Sciences} / №273-1909 г. / 781. *Brachydesmus* / *talyschanus* Lohm. / Опр. {Det.} Type. / Сбор {Collected} 12.V.1909 г. / Бакин. г. {Baku Province} / Ленкоранский у. {Lenkoran Distr.} / Лерик {Lerik} / А. Кириченко {A. Kirichenko} // 59 // *Brachydesmus* / *talyschanus* n.sp. / Type Hans Lohmander.

**Current nomenclature**. *Brachydesmus kalischewskyi* Lignau, 1915 (Sierwald et al. 2026). Synonymised with *B. kalischewskyi* by Golovatch et al. (2016).

**Remarks**. The holotype is fragmented.

***vigens* Golovatch, 1987 (*Himalodesmus* )**

*Himalodesmus vigens* Golovatch, 1987: 211, figs 20–22 (D♂).

**Types. *Paratypes***: (2♂), MYR_DIP_0000123.

**Type locality**. Nepal, Ilam Distr., Maipokhari (ca 27°01'N, 87°55'E).

**Label data**. *Himalodesmus vigens* / Golovatch, 1986. / Опр. {Det.} С. Головач {S. Golovatch} / Сбор {Collected} J. Martens & / B. Daams: Nepal / 2♂ paratypes / паратипы // *Himalodesmus vigens* / Golovatch, n. sp. / 2 ♂ паратипы, paratypes / det. S. Golovatch, 1984 // Nepal, Ilam dist., Mai Pokhari, / 2150–2250 m, 23–25.VIII.1983 / leg. J. MARTENS & B. DAAMS 254.

**Current nomenclature**. *Himalodesmus vigens* Golovatch, 1987 (Sierwald et al. 2026).

**Remarks**. The holotype ♂ and remaining paratypes (6♂) are in SMF.

### Family Trichopolydesmidae

***asceticus* Golovatch, 1986 (*Sholaphilus* )**

*Sholaphilus asceticus* Golovatch, 1986a: 365, figs 41–44 (D♂♀).

**Types. *Paratypes***: (1♂ 1♀), MYR_DIP_0000119.

**Type locality**. Nepal, Kaski Distr., between Lumle (ca 28°23'N, 83°50'E) and Khare (ca 27°50'N, 86°18'E).

**Label data**. *Sholaphilus asceticus* / Golovatch, 1985. / Опр. {Det.} С. Головач {S. Golovatch} / Сбор {Collected} J. Martens / Nepal / 1♂ 1♀ паратипы / paratypes // *Sholaphilus asceticus* / Golovatch, n. sp. / 1♂ 1♀ paratypes / det. S. Golovatch, 1983 // Nepal, Southern Annapurna / Himal, between Lumle & Khare, / 1650 m, secondary forest near stream / 16.XII.1969, leg. J. MARTENS № 4.

**Current nomenclature**. *Sholaphilus asceticus* Golovatch, 1986 (Sierwald et al. 2026).

**Remarks**. The holotype ♂ and remaining paratypes (2♂ 5♀) are in SMF.

***martensi* Golovatch, 1986 (*Sholaphilus* )**

*Sholaphilus martensi* Golovatch, 1986a: 361, figs 31–36 (D♂).

**Types. *Paratype***: (1 ♂), MYR_DIP_0000117.

**Type locality**. Nepal, Lamjung Distr., Senghe (= Syange) to Jagat (ca 28°24'N, 84°24'E).

**Label data**. *Sholaphilus martensi* / Golovatch, 1985 / Опр. {Det.} С. Головач {S. Golovatch} / Сбор {Collected} J. MARTENS & / A. AUSOBSKY; Nepal / 1♂ паратип / paratype // *Sholaphilus martensi* / Golovatch, n. sp. / 1♂ Paratype, gonopods / in slide // 137 Manang Dist., Marsyandi / 2100 m, under Bagarchap / *Pinus excels*-Wald 12 Apr 80 / MARTENS & AUSOBSKY leg. // Polydesmida.— Micropreparation: *Sholaphilus martensi* / Golovatch, n. sp. / 1♂ paratype, gonopods / Nepal, Manang distr., / Marsyandi Valley, below / Bagarchap, sparse *Pinus* / *excelsa* forest, 2100 m, / 12.IV.1980, J. MARTENS / & A. AUSOBSKY, № 137 // 0000117.

**Current nomenclature**. *Sholaphilus martensi* Golovatch, 1986 (Sierwald et al. 2026).

**Remarks**. The holotype ♂ and remaining paratype (1♂) are in SMF.

***perarmata* Golovatch, 1986 (*Hingstonia* )**

*Hingstonia perarmata* Golovatch, 1986a: 357, figs 25–28 (D♂♀).

**Types. *Paratype***: (1♂), MYR_DIP_0000121.

**Type locality**. Nepal, Mustang Distr., Thaksang above Tukuche (ca 28°47'N, 83°34'E).

**Label data**. *Hingstonia perarmata* / Golovatch, 1985 / Опр. {Det.} С. Головач {S. Golovatch} / Сбор {Collected} J. Martens / Nepal / 1 ♂ паратип / paratype // *Hingstonia perarmata* / *Golovatch*. n. sp. / 1♂ паратип / paratype / Взято за обработку из / Зенкенбергского Музея {taken for identification from Senckenberg Museum} / det. С.И. Головач {S.I. Golovatch}, 1983 // Nepal: Thakkhola: Kali Gandaki / Valley between Annapurna and / Dhaulagiri Himal: Thaksang, / above Tukche, 3150 m, 2–4 July 1973 / leg. J. Martens, 37.

**Current nomenclature**. *Hingstonia perarmata* Golovatch, 1986 (Sierwald et al. 2026).

**Remarks**. The holotype ♂ and the remaining paratypes (6♂ 3♀) are in SMF.

***variata* Golovatch, 1987 (*Hingstonia* )**

*Hingstonia variata* Golovatch, 1987: 215, figs 32–37 (D♂♀).

**Types. *Paratypes***: (1♂ 1♀), MYR_DIP_0000113.

**Type locality**. Nepal, Taplejung Distr., Deurali Pass (ca 28°24'N, 83°43'E).

**Label data**. *Hingstonia variata* / Golovatch, 1986 / Опр. {Det.} С. Головач {S. Golovatch} / Сбор {Collected} J. Martens / & B. Daams: Nepal / 1♂ 1♀ паратипы / paratypes // Nepal // *Hingstoma variata* / Golovatch, n. sp. / 1♂ 1♀ паратипы / paratypes / det. S. Golovatch, 1984 // 280 Taplejung Dist., Anda / Deorali zw. Simbua u. Gunsa / Khola 4250–4500 m alt. Matten / 9 Sep 83 J. MARTENS & B. DAAMS.

**Current nomenclature**. *Hingstonia variata* Golovatch, 1987 (Sierwald et al. 2026).

**Remarks**. The holotype ♂ and the remaining paratypes (4♂ 4♀ and 12 juv.) are in SMF.

### Family Xystodesmidae

***marinae* Golovatch, 1978 (*Rhysolus* )**

*Rhysolus marinae* Golovatch, 1978: 677, figs 1.1–2 (D♂).

**Type. *Holotype***: (♂), MYR_DIP_0000130.

**Type locality**. Japan, Kyushu, apparently Obama Onsen (ca 32°44'N, 130°12'E).

**Label data**. № 57–1909 / голотип ♂ / *Rhysolus* / *marinae* Golovatch / Опр. {Det.} Golovatch / Сбор {Collected} Япония {Japan} / 5.VIII.1907 / leg. Я. Черский {Ya. Chersky} // *Rhysolus marinae* / Golovatch / ♂ голотип {holotype}, det. / С. Головач {S. Golovatch} 1975 // № 57–1909 / Японiя, / О^в^ Кiy-Ciy / Oбaм..? [illegible text] {Japan, Kiu-Siu island, Obam..?} / 5.VIII.1907 / нов. ст. Я. Черский {Ya. Chersky}.

**Current nomenclature**. *Riukiaria marinae* (Golovatch, 1978) (Sierwald et al. 2026). Shinohara (1977) revived *Riukiaria* Attems, 1938 as a subjective senior synonym of *Rhysolus* Chamberlin & Wang, 1953.

**Remark**. The species was described from the holotype only.

***spatuliformis* Golovatch, 2015 (*Riukiaria* )**

*Riukiaria spatuliformis* Golovatch, 2015: 1026, figs 3–4 (D♂).

**Type. *Paratype***: (1♂), MYR_DIP_0000186.

**Type locality**. China, Sichuan, N of Lanan (30°3'29"N, 102°14'0"E) (Golovatch 2015).

**Label data**. *Riukiaria spatuliformis* / Golovatch, n.sp. / 1♂ paratype / det. S. Golovatch, 2015 // China, Sichuan Prov. / N. Luding city, N Lanan / 30°3'29"N, / 102°14'0"E/ 18.05.2014, H = 2525 m / Belousov I. & Kabak I. leg.

**Current nomenclature**. *Riukiaria spatuliformis* Golovatch, 2015 (Sierwald et al. 2026).

**Remarks**. The holotype ♂ and remaining paratype (1♂) are in ZMMU.

### Order Polyzoniida

**Family Polyzoniidae**

***amurensis* Gerstfeldt, 1858 (*Platydesmus* )**

*Platydesmus amurensis* Gerstfeldt, 1858: 273 (♀?).

**Type. *Syntype***: (♀?, badly fragmented), MYR_DIP_0000322.

**Type locality**. China, the mouth of Songhua River (ca 47°41N, 132°30'E).

**Label data**. 109. *Craspedosoma* / *amurensis* Gerstf. / Опр. {Det.} Spec. orig. / Сбор {Collected} // *Craspedosoma amurensis* Gerstf. // 109. *Polyzonium* sp. / Опр. {Det.} det. С. Головач {S. Golovatch} / Сбор {Collected}.

**Current nomenclature**. *Angarozonium amurense* (Gerstfeldt, 1858) (Sierwald et al. 2026); this is a trans-Siberian species (e.g., Mikhaljova 2017).

**Remarks**. The syntype is badly damaged and fragmented into small pieces.

### Order Sphaerotheriida

**Family Sphaerotheriidae**

***compressum* Brandt, 1833 (*Sphaerotherium* )**

*Sphaerotherium compressum* Brandt, 1833: 198 (D♂♀).

**Type. *Paralectotype***: (♀), MYR_DIP_0000015.

**Type locality**. South Africa, Cape of Good Hope (no exact locality).

**Label data**. *Sphaerotherium compressum* / Опр. {Det.} Brandt / Сбор {Collected} Caput bonae spei / Brandt / Lectotype / Лектотип.

**Current nomenclature**. *Sphaerotherium compressum* Brandt, 1833 (Sierwald et al. 2026). The lectotype was designated by VandenSpiegel et al. (2002).

**Remarks**. Two specimens, the lectotype ♂ and one paralectotype (♀), are in ZMB. The ZISP paralectotype is broken: its half is kept in one vial and some detached legs in another.

***elongatum* Brandt, 1833 (*Sphaerotherium* )**

*Sphaerotherium elongatum* Brandt, 1833: 199 (D♂♀).

**Type. *Paralectotype***: (1♂), MYR_DIP_0000037.

**Type locality**. South Africa, Cape of Good Hope (no exact locality).

**Label data**. *Sphaerotherium elongatum* / Brandt, 1833. / syntype, Caput Bonae Spei // lectotypus // *Sp. elongatum* / *C. bonae* Sp. Br.

**Current nomenclature**. *Sphaerotherium elongatum* Brandt, 1833 (Sierwald et al. 2026). The lectotype was designated by VandenSpiegel et al. (2002).

**Remarks**. Five specimens, the lectotype ♂ and remaining paralectotypes (1♂ 3♀) are in ZMB. The ZISP sample contains a single specimen and a separate tube with detached telopods.

***klugii* Brandt, 1841 (*Sphaerotherium* )**

*Sphaerotherium klugii* Brandt, 1841b: 361 (D♀).

**Type. *Lectotype***: (♀), MYR_DIP_0000017.

**Type locality**. South Africa, Cape of Good Hope (no exact locality).

**Label data**. *Sphaeroperium klugii* / Brandt / Опр. {Det.} Brandt / Сбор {Collected} Caput bonae spei / leg. Brandt / типовой экз. {type specimen} / Lectotypus design. S. // sph. klugii / M. / *C. bonea* spei / Brandt Golovatch.

**Current nomenclature**. *Sphaerotherium compressum* Brandt, 1833 (Sierwald et al. 2026). The lectotype was designated by Golovatch and Hoffman (2000). Synonymised with *S. compressum* by VandenSpiegel et al. (2002).

**Remarks**. The lectotype dried and was moistened on 26.XI.2025; it is broken into two pieces, with some detached legs being kept in a separate microvial.

***lichtensteinii* Brandt, 1833 (*Sphaerotherium* )**

*Sphaerotherium lichtensteinii* Brandt, 1833: 199 (D♀).

**Type. *Lectotype***: (♀), MYR_DIP_0000016.

**Type locality**. South Africa, Cape of Good Hope (no exact locality).

**Label data**. *Sphaeroperium lichtensteinii* / Brandt, 1833 / Опр. {Det.} Brandt / Сбор {Collected} Caput bonae spei / leg. Brandt / Lectotypus ♀ / design. S. Golovatch // lectotypus // sph. Lichtenstein / Nob.

**Current nomenclature**. *Sphaerotherium lichtensteinii* Brandt, 1833 (Sierwald et al. 2026). The lectotype was designated by VandenSpiegel et al. (2002).

**Remarks**. The paralectotype ♀ is in ZMB. The ZISP sample contains a single broken specimen, with detached legs being kept in a separate tube.

***microstictum* Brandt, 1841 (*Sphaerotherium* )**

*Sphaerotherium microstictum* Brandt, 1841b: 362 (D♂♀).

**Types. *Lectotype***: (♀), MYR_DIP_0000041. ***Paralectotypes***: (2♂ 1♀ 3 juv.), MYR_DIP_0000014; (1♂), MYR_DIP_0000051.

**Type locality**. South Africa, Cape of Good Hope (no exact locality).

**Label data**. MYR_DIP_0000041: *Sphaerotherium microstictum* / Brandt / Опр. {Det.} Brandt / Сбор {Collected}. Caput Bonae spei / Brandt / ♀ lectotype лектотип // *Sphaer*. / microstictum / Nob. / *C. bonae* spei / Brandt.

MYR_DIP_0000014: *Sphaerotherium microstictum* / Brandt / Опр. {Det.} Brandt / Сбор {Collected} Caput bonae spei / Brandt / паралектотипы / paralectotypes // af. *S. insignis* / det. / loc. / rec. / R.G. Mus. Afr. Centr.

MYR_DIP_0000051: *Sphaerotherium elongatum* / from *S. microstictum* / paralectotypus.

**Current nomenclature**. *Sphaerotherium elongatum* Brandt, 1833 (Sierwald et al. 2026). The lectotype was designated by VandenSpiegel et al. (2002), who also synonymised this species with *S. elongatum*.

**Remarks**. The remaining paralectotypes (3♀) are in ZMB. The lectotype dried out and was moistened on 17.X.2024. The sample MYR_DIP_0000014 contains six specimens and two separate microvials with detached telopods and other body parts; all adults in this sample are broken into two parts. The sample MYR_DIP_0000051 has no geographic label; it contains a broken specimen and a separate microvial with telopods.

***punctulatum* Brandt, 1841 (*Sphaerotherium* )**

*Sphaerotherium punctulatum* Brandt, 1841b: 362 (D♂♀).

**Types. *Lectotype***: (♂), MYR_DIP_0000036. ***Paralectotype***: (1♀), MYR_DIP_0000013.

**Type locality**. South Africa, Cape of Good Hope (no exact locality).

**Label data**. MYR_DIP_0000036: *Sphaerotherium* / punctulatum Brandt / Опр. {Det.} Brandt / Сбор {Collected} Caput Bonae spei / Brandt / ♂ lectotype // *sph. punctulatum* / C.B. Spei Brandt лектотип {lectotype}.

MYR_DIP_0000013: *Sphaerotherium* / punctulatum Brandt / Опр. {Det.} Brandt / Сбор {Collected} Caput bonae spei / Brandt / ♀ paralectotypes / паралектотип.

**Current nomenclature**. *Sphaerotherium punctulatum* Brandt, 1841 (Sierwald et al. 2026). The lectotype was designated by Golovatch and Hoffman (2000), but see Wesener (2016: 60).

**Remarks**. This species was described from two specimens (1♂ 1♀). The lectotype ♂ dried out and was moistened on 17.X.2024; the sample contains a broken specimen and a separate microvial with detached body parts. The paralectotype is broken, with detached legs being placed in a separate tube.

***rugulosum* Brandt, 1841 (*Sphaerotherium* )**

*Sphaerotherium rugulosum* Brandt, 1841b: 363 (D♀).

**Type. *Lectotype***: (♀), MYR_DIP_0000012.

**Type locality**. South Africa, Cape of Good Hope (no exact locality).

**Label data**. *Sphaeroperium rugulosum* / Brandt / Опр. {Det.} Brandt / Сбор {Collected} Caput bonae spei / leg. Brandt / типовой экз. {type specimen} // *Sphaeropherium* / rugulosum / *С. bonae* spei Brandt // 66.

**Current nomenclature**. *Sphaerotherium rugulosum* Brandt, 1841 (Sierwald et al. 2026). The lectotype was designated by Golovatch and Hoffman (2000), but see Wesener (2016: 60). The validity of this species name is doubtful, with a potential synonymy with *S. punctatum* Brandt, 1833; see VandenSpiegel et al. (2002) for further details.

**Remarks**. The lectotype is fragmented, with detached legs and other body parts being kept in two microvials.

***titanus* Brandt, 1841 (*Sphaerotherium* )**

*Sphaerotherium titanus* Brandt, 1841b: 361 (D♀).

**Type. *Holotype***: (♀), MYR_DIP_0000011.

**Type locality**. Madagascar (no exact locality).

**Label data**. *Sphaerotherium titanus* / Brandt / Опр. {Det.} Brandt / Сбор {Collected} Patria ignota, / leg. Dupont, 1838 / голотип / holotype // *Sphaerotherium* / *titanus* Br. / Dupont P (?) atia ? / 1838.

**Current nomenclature**. *Sphaerotherium titanus* Brandt, 1841 (Sierwald et al. 2026).

**Remarks**. It was mistakenly assumed that the holotype of *S. titanus* had been lost (Wesener 2016: 93), but it is available. The holotype has its legs detached and kept in a separate microvial.

### Family Zephroniidae

***insignis* Brandt, 1833 (*Sphaeropoeus* )**

*Sphaeropoeus insignis* Brandt, 1833: 200 (D♂♀).

**Type. *Lectotype***: (♂), MYR_DIP_0000026.

**Type locality**. Indonesia, Java (no exact locality).

**Label data**. *Sphaeropoeus insignis* / Brandt, 1833 / Опр. {Det.} Brandt / Сбор {Collected} Java Fritze / Dr. Bartels / 1 ♂ лектотип / lectotypus // Fritze Java // Sph. Insignis / Java Dr. Bartels // Lectotypus / design. S. Golovatch.

**Current nomenclature**. *Castanotherium insignis* (Brandt, 1833) (Sierwald et al. 2026). Transferred to the genus *Castanotherium* Pocock, 1895 by Jeekel (2001).

**Remarks**. According to Golovatch and Hoffman (2000: 238), the ZISP collection should hold a series of five type specimens (2♂ 2♀ 1 juv.), but only the lectotype ♂ is currently available.

### Order Spirobolida

**Family Pachybolidae**

***elegans* Brandt, 1840 (*Julus* )**

Fig. 3

*Julus elegans* Brandt, 1840c: 125 (D♂♀).

**Types. *Lectotype***: (♂), MYR_DIP_0000019. ***Paralectotype***: (1♀), MYR_DIP_0000050.

**Type locality**. South Africa, Cape of Good Hope (no exact locality).

**Label data**. MYR_DIP_0000019: *Chersastus elegans* (Brandt), 1841 / Опр. С.И. Головач {Det. S.I. Golovatch} / Сбор Южная Африка {Collected South Africa} / leg. Brandt / 1♂ лектотип {lectotype} // Julus (Spirobolus) / *elegans* Brandt, 1841 / Опр. {Det.} Brandt / Сбор {Collected} Afrique austr. / leg. Brandt.

MYR_DIP_0000050: Julus (Spirobolus) / *elegans* Brandt, 1841 / Опр. {Det.} Brandt / Сбор {Collected} Africa austr., / Caput Bonae Spei / leg. Brandt / 1♀ синтип {syntype} // Paralectotypus ♀ // 10 // *Chersastus* sp. / 1♀ / Опр. С.И. Головач {Det. S.I. Golovatch}, 1979 / Сбор {Collected} Мыс Доброй Надежды {Cape of Good Hope} / leg. Brandt / (обозначен как синтип *Spirobolus* / *elegans* Brandt, 1841, но таковым / не является) {designated as the syntype of *Spirobolus elegans* Brandt, 1841, but it is not}.

**Current nomenclature**. Listed as a junior synonym of *Centrobolus anulatus* (Attems, 1934) by Sierwald et al. (2026), contrary to Golovatch and Hoffman (2000: 235) who treated it as a valid species, considering it is at least much older and different from *C. elegans* in the sense of Porat (1872), also transferring this species to *Centrobolus* Cook, 1897.

**Remarks**. In the original description, Brandt (1840a: 126) mentioned that the type series contained “numerosa specimina”, of which currently only three specimens are available: two in ZISP (see above) and one incomplete paralectotype in ZMB (see Golovatch and Hoffman 2000). The lectotype is fragmented, its body pieces are kept in one vial and its gonopods in another (Fig. 3A). The paralectotype is an intact specimen which in fact belongs to another species of *Centrobolus*.

### Family Rhinocricidae

***grandis* Brandt, 1840 (*Julus* )**

Fig. 4

*Julus grandis* Brandt, 1840c: 123 (D♀).

**Type. *Lectotype***: (♀), MYR_DIP_0000023.

**Type locality**. Brazil (no exact locality).

**Label data**. *Julus (Spirostreptus) grandis *(sic.) / Brandt, 1841 / Опр. {Det.} Brandt / Сбор {Collected} Brasilia / 1♀ lectotypus // *Julus spirostreptus* / Nob. / Fem. / Brasilia.

**Current nomenclature**. Accepted as *Julus grandis* Brandt, 1840 by Sierwald et al. (2026). However, this species seems to actually belong to the genus *Anadenobolus* Silversti, 1897, thus representing a new combination: *Anadenobolus grandis* (Brandt, 1840), comb. nov. ex *Spirobolus* Brandt, 1833 (det. SIG).

**Remarks**. Although Brandt (1840c: 124) wrote that he had “vidi tria specimina”, only one specimen is available in the ZISP collection; the whereabouts of two others and their sex remain unknown. The jar contains three vials with body fragments of the lectotype.

***marcgravii* Brandt, 1840 (*Julus* )**

*Julus marcgravii* Brandt, 1840c: 124 (D♂♀).

**Types. *Syntypes***: (1♀), MYR_DIP_0000039; (1♂), MYR_DIP_0000047.

**Type locality**. Brazil (no exact locality).

**Label data**. MYR_DIP_0000039: *Julus (Spirostreptus)* / *marcgravii* Brandt, 1841 / Опр. {Det.} Golovatch-Kriv[okhatsky] / Сбор {Collected} Riedel / Brasil ♀ / №3 // Bras. / №3.

MYR_DIP_0000047: *Julus (Spirobolus)* / *marcgravii* Brandt, 1841 / Опр. {Det.} Golovatch-Kriv[okhatsky] / Сбор {Collected} Riedel ♂ / Brasil // *Julus (Spirobolus)* / *maximus* Nob. / var. d. / Brasilia Riedel.

**Current nomenclature**. Accepted as *Julus marcgravii* Brandt, 1841 by Sierwald et al. (2026), but Golovatch and Hoffman (2000: 235) provided important notes. This name was introduced by Brandt in 1840, not in 1841. This species actually belongs to the genus *Anadenobolus* Silversti, 1897, thus representing a new combination: *Anadenobolus marcgravii* (Brandt, 1840), comb. nov. ex *Spirobolus* Brandt, 1833 (det. SIG).

**Remarks**. Brandt (1840c: 124) studied three specimens (“tria specimina adulta”), of which only two are currently available.

***olfersii* Brandt, 1833 (*Spirobolus* )**

*Spirobolus olfersii* Brandt, 1833: 202 (D♀).

**Type. *Holotype***: (♀), MYR_DIP_0000021.

**Type locality**. Brazil (no exact locality).

**Label data**. *Julus olfersii* / Brandt, 1833 / Опр. {Det.} Brandt / Сбор {Collected} Brasilia, / Riedel Spei / 1♀ (fragm.) lectotypus / лектотип // n.8*. Julus* (*Spirobolus*) / *olfeus* Nob. / Brasilia. Riedel.

**Current nomenclature**. *Spirobolus olfersii* Brandt, 1833 (Sierwald et al. 2026). However, this species actually belongs to the genus *Anadenobolus* Silversti, 1897, thus representing a new combination: *Anadenobolus olfersii* (Brandt, 1833), comb. nov. ex *Spirobolus* Brandt, 1833 (det. SIG).

**Remarks**. This species was described from the holotype ♀ alone. The holotype represents a body fragment.

### Family Spirobolidae

***bungii* Brandt, 1833 (*Spirobolus* )**

*Spirobolus bungii* Brandt, 1833: 203 (D♀).

**Types. *Syntypes***: (2♀), MYR_DIP_0000032.

**Type locality**. China, around Beijing (ca 39°54'N, 116°25'E).

**Label data**. *Spirobolus bungii* / Brandt / Опр. {Det.} Brandt / Сбор {Collected} China borealis, / proper Peking / 2♀ синтипы / syntypes // *Spirobolus* / *Bungii*.

**Current nomenclature**. *Spirobolus bungii* Brandt, 1833 (Sierwald et al. 2026).

**Remarks**. The sample contains body fragments of two specimens; both syntypes dried out and were moistened on 26.XI.2025.

### Order Spirostreptida

**Family Iulomorphidae**

***rotundatus* Brandt, 1840 (*Julus* )**

Fig. 5

*Julus rotundatus* Brandt, 1840c: 119 (D♂).

**Type. *Holotype***: (♂), MYR_DIP_0000022.

**Type locality**. South Africa (no exact locality).

**Label data**. *Julus (Spirostreptus)* / *rotundatus* Brandt / Опр. {Det.} Brandt / Сбор {Collected} Africa austr. / Riedel Spei / 1♂ голотип {holotype} / гоноподии в пробирке {gonopods are in a vial} // 121 // Holotypus.

**Current nomenclature**. *Spirostreptus rotundatus* (Brandt, 1840) (Sierwald et al. 2026). Transferred to *Spirostreptus* Brandt, 1833 by Hamer (1998), but in fact this species belongs to the South African genus *Iulomorpha* Porat, 1872, thus forming a new combination: *Iulomorpha rotundata* (Brandt, 1840), comb. nov. ex *Julus* Linnaeus, 1758 (det. SIG).

**Remarks**. This species was described from the holotype ♂ only. The jar contains two vials: one with body fragments and the other with the gonopods (Fig. 5).

### Family Harpagophoridae

***attenuatus* Brandt, 1840 (*Julus* )**

*Julus attenuatus* Brandt, 1840c: 108 (D♂).

**Types. *Lectotype***: (♂), MYR_DIP_0000052.

**Type locality**. South Africa (no exact locality).

**Label data**. *Julus (Spirostreptus) */ *attenuatus* Brandt / Опр. {Det.} Brandt / Сбор {Collected}. Afrique merid. / 1♂ голотип {holotype} // 83. // *Harpogophora* / *attenuata* (Brandt, 1841) / Опр. {Det.} Brandt / Сбор {Collected} Afrique merid[dionale] / (Южн. Африка {South Africa}) leg. Brandt / 1♂ lectotypus.

**Current nomenclature**. *Harpagophora attenuata* (Brandt, 1840) (Sierwald et al. 2026). Transferred to the genus *Harpagophora* Attems, 1909 by Hoffman and Golovatch (1998), who also designated the lectotype of this species.

**Remarks**. In the original description (Brandt 1840c: 109), the type series is described as consisting an adult ♂ and a juvenile. However, Hoffman and Golovatch (1998) clarified that the second syntype was indeed a female. At present, this female is absent from the ZISP collection. The ZISP sample contains two vials: one with the broken lectotype ♂, the other with its left gonopod.

***capensis* Brandt, 1840 (*Julus* )**

Fig. 6

**Figure 6.**
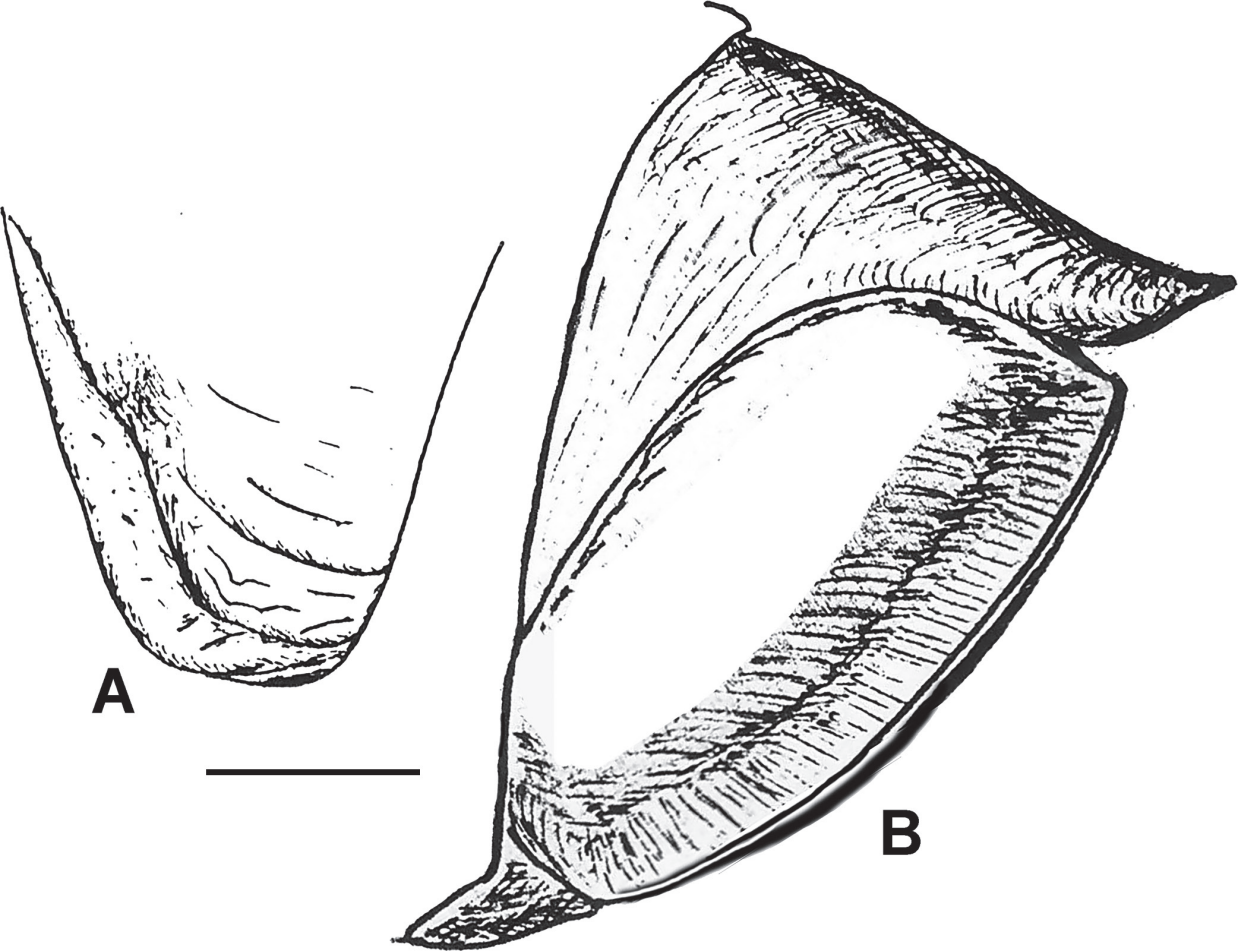
*Poratophilus capensis* (Brandt, 1840), comb. nov. (= Julus (Spirostreptus) capensis Brandt, 1840), ♀ holotype. **A**. Ventral half of collum, lateral view; **B**. Telson, lateral view. Scale bar: 10 mm. SIG del.

*Julus capensis* Brandt, 1840c: 107 (D♀).

**Type. *Holotype***: (♀), MYR_DIP_0000292.

**Type locality**. South Africa, Cape of Good Hope (no exact locality).

**Label data**. Зоол. Муз. АН {Zoological Museum of the Academy of Sciences} / Julus (Spirostreptus) capensis Brandt, 1841 / Опр. {Det.} Brandt J.-F. / Сбор {Collected} / Caput Bonae Spei / leg. Brandt / 1♀ голотип holotype // Зоол. Муз. АН {Zoological Museum of the Academy of Sciences} / *Paratophilus capensis* (Brandt) / Опр. {Det.} С.И. Головач {S.I. Golovatch}, 1979 / Сбор {Collected} / Мыс Доброй Надежды, {Cape of Good Hope} / leg. J.-F. Brandt / 1♀ голотип holotype // Julus (Spirostreptus) / *capensis* Nob. / Ann. iol anali. 44. / Aucticos [illegible] / par. ultinuis 2 apodi / Pars. 79 / Cap. b. sp. Brandt.

**Current nomenclature**. Accepted as *Spirostreptus capensis* (Brandt, 1840) by Sierwald et al. (2026). Transferred to the genus *Spirostreptus* Brandt, 1833, but treated as a nomen obscurum by Hamer (1998). Based on the shapes of the collum and especially the telson (Fig. 6), this species actually belongs to the genus *Poratophilus* Silvestri, 1897), thus marking a new combination: *Poratophilus capensis* (Brandt, 1840), comb. nov. ex *Julus* Linnaeus, 1758 (det. SIG).

**Remarks**. The holotype is dried and pinned. The third label of the holotype is illegible.

***gracilis* Brandt, 1840 (*Julus* )**

Fig. 7

**Figure 7.**
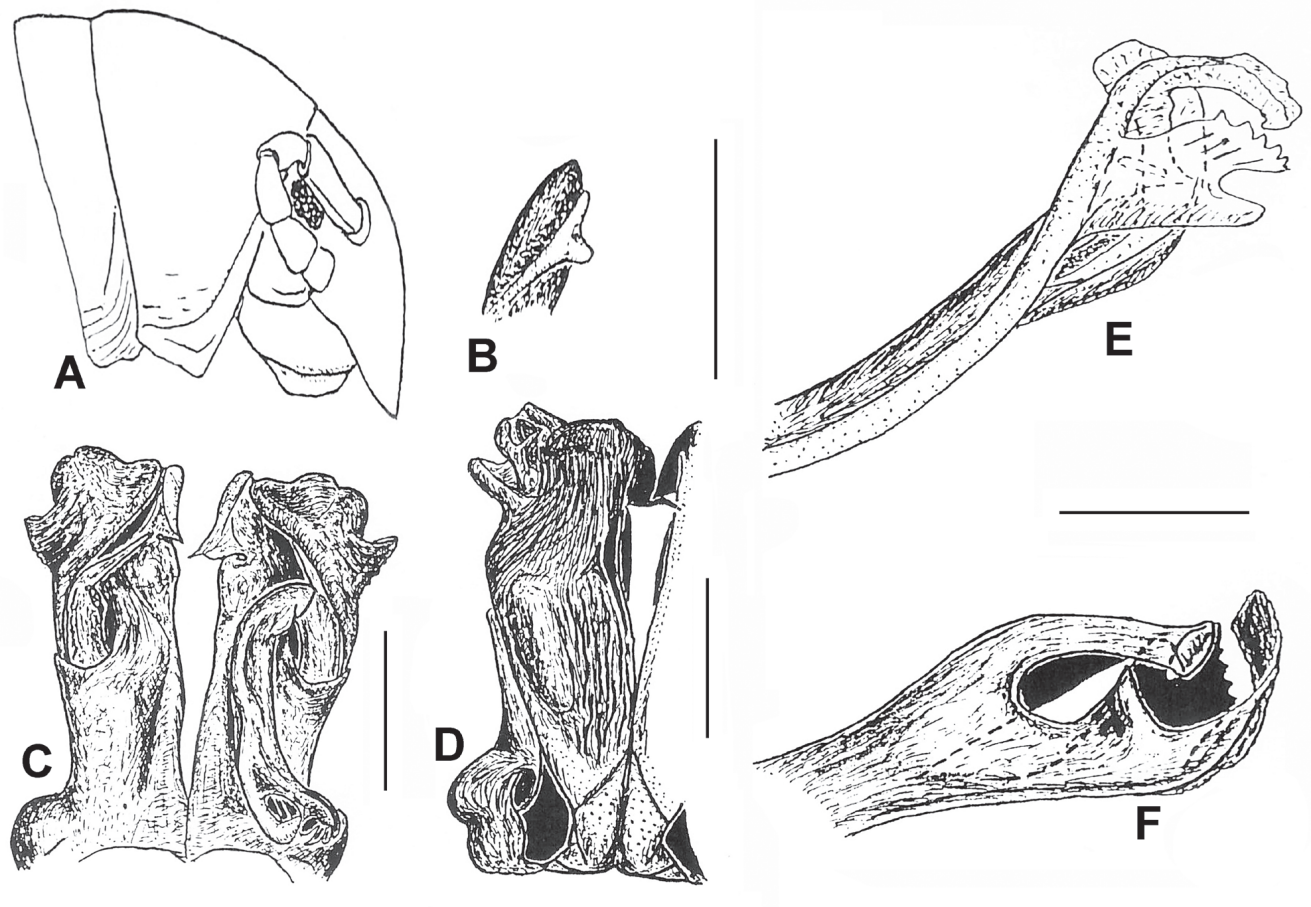
*Zinophora gracilis* Brandt, 1840 (= Julus (Spirostreptus) gracilis Brandt, 1840), ♂ holotype. **A**. Anterior part of body; **B**. Left gena, ventral view; **C, D**. Gonopods, posterior and anterior views, respectively; **E, F**. Tip of gonopodal telopodite, different aspects. Scale bars: 1.0 mm (**A–D**); 0.5 mm (**E, F**). SIG del.

*Julus gracilis* Brandt, 1840c: 108 (D♂).

**Type. *Holotype***: (♂), MYR_DIP_0000053.

**Type locality**. South Africa, Cape of Good Hope (no exact locality), but Redman et al. (2003) fixed the type locality as Lesotho, Masite Mt. (= Masiti) (ca 29°36'S, 27°27'E).

**Label data**. *Julus gracilis* Brandt / Опр. {Det.} det. Brandt / Сбор {Collected} Afrique / Caput Bonae spei. / ♂ голотип // *Harpogophora* / *gracilis* (Brandt, 1841) / Опр. С.И. Головач {Det. S.I. Golovatch} / Сбор {Collected} Мыс Доброй Надежды {Cape of Good Hope} / leg. Brandt / ♂ голотип {holotype} // 94.

**Current nomenclature**. *Zinophora gracilis* (Brandt, 1840) (Sierwald et al. 2026). Transferred to the genus *Zinophora* Chamberlin, 1927 by Redman et al. (2003), based on an unpublished drawing of the holotype gonopods (courtesy of SIG), reproduced here (Fig. 7).

**Remarks**. The species was described from the holotype ♂ alone. The jar contains two vials: one with a broken holotype, the other with its gonopods.

***javanicus* Brandt, 1841 (*Julus* )**

*Julus javanicus* Brandt, 1841a: 92 (D♂♀).

**Types. *Lectotype***: (♂), MYR_DIP_0000029. ***Paralectotype***: (1♀), MYR_DIP_0000031; (1♂), MYR_DIP_0000042; (1♀), MYR_DIP_0000043.

**Type locality**. Indonesia, Java (no exact locality).

**Label data**. MYR_DIP_0000029: *Julus (Spirostreptus)* / *javanicus* №2. / ♂ / Java Fritze // *Thyropygus* / *javanicus* (Brandt), 1841 / Опр. С. Головач {Det. S. Golovatch} / Сбор {Collected} Java, / Fritze / 1♂ лектотип / lectotypus // *Remulopygus javanicum* / (Brandt) / det. Hoffman & Golovatch, 1998.

MYR_DIP_0000031: *Julus (Spirostreptus)* / *javanicus* №6. / Mas / Java Fritze // *Thyropygus* / *javanicus* (Brandt), 1841 / Опр. С. Головач {Det. S. Golovatch} / Сбор {Collected} Java, Fritze / 1♀ паралектотип {paralectotype} // *Remulopygus javanicum* / (Brandt) / det. Hoffman & Golovatch, 1998.

MYR_DIP_0000042: Julus* (Spirostreptus)* / *javanicus* Nob. / fem. / ova adulta // *Thyropygus* / *javanicus* (Brandt), 1841 / Опр. С. Головач {Det. S. Golovatch} / Сбор {Collected} Java / 1♂ паралектотип {paralectotype}.

MYR_DIP_0000043: Зоол. Инст. АН {Zoological Institute of the Academy of Sciences} / *Julus (Spirostreptus)* / *javanicus* Brandt / Опр. {Det.} J.-F. Brandt / Сбор {Collected} Java / Brandt / 1♀ паралектотип {paralectotype} // Зоол. Инст. АН {Zoological Institute of the Academy of Sciences} / *Thyropygus* / *javanicus* (Brandt), 1841 / Опр. С. Головач {Det. S. Golovatch} / Сбор {Collected} Java / Fritze / 1♀ паралектотип {paralectotype} // *Remulopygus javanicus* / (Brandt) / Место {Site} Java / ♀ paralectotype // Paralectotypus / design. S. Golovatch.

**Current nomenclature**. *Remulopygus javanicus* (Brandt, 1841) (Sierwald et al. 2026). Transferred to the genus *Remulopygus* Demange, 1961 by Hoffman and Golovatch (1998).

**Remarks**. The lectotype is fragmented. The sample MYR_DIP_0000042 contains the 7^th^ segment with the gonopods, and the sample MYR_DIP_0000043 contains a single broken specimen and several labels.

***pachysoma* Brandt, 1840 (*Julus* )**

*Julus pachysoma* Brandt, 1840c: 109 (D♂♀).

**Types. *Lectotype***: (♂), MYR_DIP_0000024. ***Paralectotype***: (1♀), MYR_DIP_0000040.

**Type locality**. South Africa, Cape of Good Hope (no exact locality).

**Label data**. MYR_DIP_0000024: синтип {syntype} / Зоол. Инст. АН {Zoological Institute of the Academy of Sciences} / *Julus (Spirostreptus)* / *pachysoma* Brandt. / Опр. {Det.} Brandt 1♂ / Сбор {Collected} Afrique austr. // *Harpagophora attenuatus* / (Brandt) ♂ / Место {Site} Южная Африка, Мыс / Доброй Надежды {South Africa, Cape of Good Hope} / Дата {Date} det. S. Golovatch – С.И. Головaч / Колл. {Collected} lectotype ♀ *Julus (Spirostreptus)* / *pachysoma* Brandt.

MYR_DIP_0000040: *Julus (Spirostreptus) / pachysoma* Brandt. / Опр. {Det.} Brandt. / Сбор {Collected} Afrique austr. / синтип 1♀ {syntype} // *Harpagophora attenuatus* / (Brandt), 1841 1♀ / Опр. С.И. Головач {Det. S.I. Golovatch}, 1979 / Сбор {Collected} Южная Африка / Мыс Доброй Надежды {South Africa, Cape of Good Hope} / leg. Brandt // *Julus (Spirostreptus) / pachysoma* Nob. / ♀ / Afr. australis. Brandt // 82.

**Current nomenclature**. *Harpagophora attenuata* (Brandt, 1840) (Sierwald et al. 2026). Synonymised with *H. attenuata* by Hoffman and Golovatch (1998), who also designated the lectotype.

**Remarks**. In the original description, Brandt (1840c: 109) also mentioned two juveniles as part of the type series. These specimens are currently absent from the ZISP collection. The lectotype was moistened in alcohol and consists of several body fragments, lacking the rear part of the body; its gonopods are in a separate vial.

### Family Odontopygidae

***bicuspidatus* Brandt, 1840 (*Julus* )**

Fig. 8

**Figure 8.**
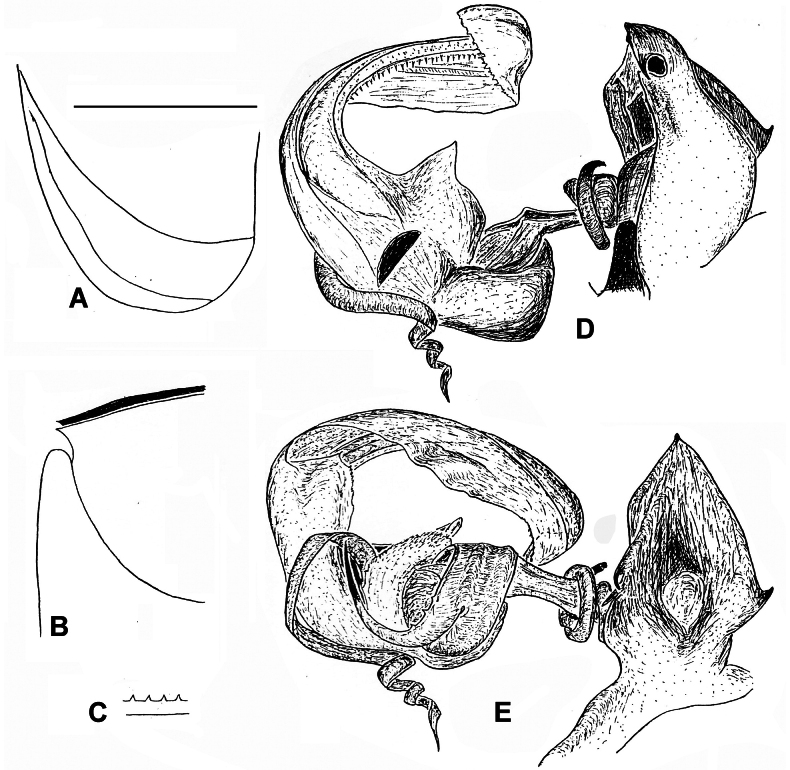
*Spinotarsus bicuspidatus* (Brandt, 1840), comb. nov. (= Julus (Spirostreptus) bicuspidatus Brandt, 1840), ♂ lectotype. **A**. Ventral half of collum; **B**. Dorso-lateral part of telson; **C**. Limbus of a midbody ring; **D, E**. Left gonopod, anterior and posterior views, respectively. Scale bar 1.0 mm (**A, B, D, E**); not to scale (**C**). SIG del.

*Julus bicuspidatus* Brandt, 1840c: 120 (D♂).

**Types. *Lectotype***: (♂), MYR_DIP_0000035. ***Paralectotype***: (1♂), MYR_DIP_0000025.

**Type locality**. South Africa, Cape of Good Hope (no exact locality).

**Label data**. MYR_DIP_0000035: *Julus (Spirostreptus)* / *bicuspidatus* Brandt / Опр. {Det.} Brandt / Сбор {Collected} Africa austr. / 1♂ голотип / (гоноподии в пробирке) {gonopods are ina vial) // *Spinotarsus* / *bicuspidatus* (Brandt), 1841 / Опр. С.И. Головач {Det. S.I. Golovatch} / Сбор Южная Африка {Collected South Africa} / leg. Brandt / 1♂ лектотип / lectotype.

MYR_DIP_0000025: *Julus (Spirostreptus) */ *bicuspidatus* Brandt, 1841 / Опр {Det.} Brandt / Сбор {Collected} Afrique austr. / Caput Bonae spei. / leg. Brandt / 1 ♂ синтип {syntype} // *Spinotarsus lineatus* / Attems, 1928, 1♂ / Опр. С.И. Головач {Det. S.I. Golovatch} / Сбор {Collected} Южная Африка / Мыс Доброй Надежды {South Africa, Cape of Good Hope}/ leg. J.-F. Brandt / (на самом деле этот синтип ♂ / *S. bicuspidatus* к таковому / не относится) {in fact, this syntype ♂ of *S. bicuspidatus* does not belong to [this species]}.

**Current nomenclature**. *Spirostreptus bicuspidatus* (Brandt, 1840) (Sierwald et al. 2026), a nomen dubium still referred to as such (Kraus 1960), vs ?*Odontopyge bicuspidata* Brandt, as suggested by Attems (1914: 219). This species belongs in fact to the genus *Spinotarsus* Attems, 1909, thus forming a new combination: *Spinotarsus bicuspidatus* (Brandt, 1840), comb. nov. ex *Julus* Linnaeus, 1758 (det. SIG, see Fig. 8).

The lectotype was indirectly designated by Golovatch and Hoffman (2000: 236), who mistakenly indicated it as the holotype.

***flavotaeniatus* Brandt, 1840 (*Julus* )**

Fig. 9

**Figure 9.**
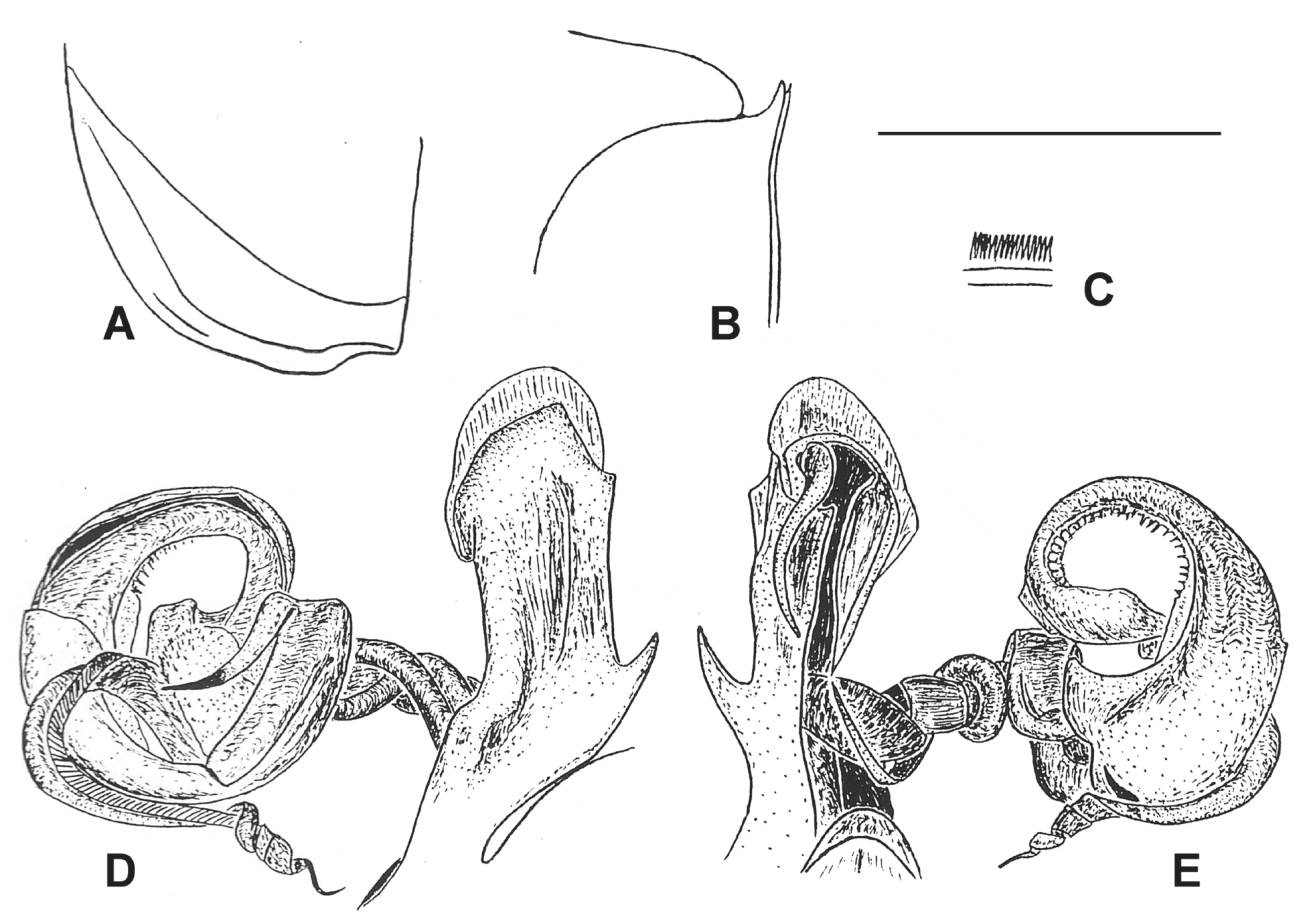
*Spinotarsus flavotaeniatus* (Brandt, 1840), comb. nov. (= Julus (Spirostreptus) flavotaeniatus Brandt, 1840), ♂ lectotype. **A**. Ventral part of collum; **B**. Dorso-caudal part of telson; **C**. Limbus of a midbody ring; **D, E**. Right gonopod, anterior and posterior views, respectively. Scale bar 1.0 mm (**A, B, D, E**); not to scale (**C**). SIG del.

*Julus flavotaeniatus* Brandt, 1840c: 121 (D♂♀).

**Type. *Lectotype***: (♂), MYR_DIP_0000038. ***Paralectotype***: (1♀), MYR_DIP_0000294.

**Type locality**. South Africa, Cape of Good Hope (no exact locality).

**Label data**. MYR_DIP_0000038 (spirit-preserved specimen): *Spinotarsus* / *flavotaeniatus* Brandt, 1841 / Опр. {Det.} С.И. Головач {S.I. Golovatch} / Сбор {Collected}, Южная Африка {South Africa}; Caput Bonae Spei / leg. Brandt / 1♂ лектотип / lectotype // lectotype // *Sp. elongatum* / *C. bonae* Sp. Br // Hololectotypus 1♂ // Annali / 66 / mai ad. / *Jul.* (Spin/r) / *flavotaeniatus* / Africa aadt. Brandt // 118.

MYR_DIP_0000294 (dried and pinned specimen): Paralectotype / design. S. Golovatch // *Julus (Spirostreptus)* / *flavotaeniatus* Brandt, 1841 / Опр. {Det.} Brandt / Сбор {Collected} Africa merid. / Caput Bonae Spei / leg. Brandt / синтип {syntype} // ? *Spinotarsus* sp. 1♀ / (*nec S. flavotaeniatus* (Brandt)) / Опр. {Det.} С.И. Головач {S.I. Golovatch} / Сбор {Collected} Южная Африка {South Africa} / Мыс Доброй Надежды {Cape of Good Hope} / leg. Brandt // *Julus (Spirostreptus) flavo*- / *fasciatus* Nob. / fem / Annal. 69 altia et penalti / neo [illegible] Annuli 5 an / Zes. 1 ped. pasi reliquis 2. / Pedils 131 pat / Africa meridionalis Brandt // 117.

**Current nomenclature**. This species name is considered a nomen nudum by Sierwald et al. (2026). The lectotype was designated by Golovatch and Hoffman (2000). The species was transferred to the genus *Spirostreptus* Brandt, 1833 by Hamer (1998), but in fact it belongs to the genus *Spinotarsus* Attems, 1909, thus forming a new combination: *Spinotarsus flavotaeniatus* (Brandt, 1840), comb. nov. ex *Julus* Linnaeus, 1758 (det. SIG, see Fig. 9).

**Remarks**. The lectotype is broken and its gonopods are kept in a separate vial. The third label of the lectotype is rather illegible.

***triplicatus* Brandt, 1840 (*Julus* )**

Fig. 10

**Figure 10.**
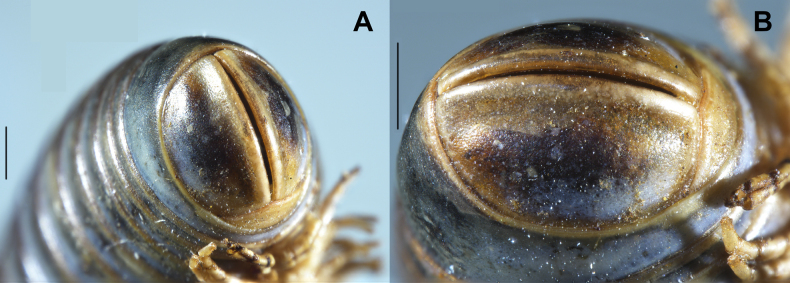
*“Spirostreptus” triplicatus* (Brandt, 1840) (= Julus (Spirostreptus) triplicatus Brandt, 1840), ♀ holotype. **A, B**. Posterior part of body, caudolateral views. Scale bar: 1 mm. Photographed by DVL.

*Julus triplicatus* Brandt, 1840c: 113 (D♀).

**Type. *Holotype***: (♀), MYR_DIP_0000291.

**Type locality**. South Africa, Cape of Good Hope (no exact locality).

**Label data**. Holotype ♀ / Зоол. Муз. АН {Zoological Museum of the Academy of Sciences} / *Julus (Spirostreptus)* / *triplicatus* Brandt, 1841 / Опр. {Det.} Brandt / Сбор {Collected} / Caput Bonae Spei / (Мыс Доброй Надежды) {Cape of Good Hope} / 1♀ голотип {holotype} // *Julus (Spirostreptus) tri*- / *plicatus* Nob. / fem / Annal. inol. anal. 61 Oribur. / post. apagi[illegible], anfesion 5 / pegum. paribus Jolivariis, idi- / suis genuisur. Pad, pars. III / Africa [illegible] Brandt.

**Current nomenclature**. *Spirostreptus triplicatus* (Brandt, 1840) (Sierwald et al. 2026). Transferred to the genus *Spirostreptus* Brandt, 1833 by Hamer (1998), but treated as a nomen obscurum. This agrees well with the structure of the telson, as only the posterior part of the body has survived. Yet the genus *Spirostreptus* is clearly wrong (Fig. 10; det. SIG) and it is best to place the generic assignment in quotation marks as until the correct genus can be determined.

**Remarks**. The holotype is dried and pinned, its head is missing. The second label of the holotype is rather illegible.

### Family Spirostreptidae

***audouini* Brandt, 1833 (*Julus* )**

Fig. 1B

*Julus audouini* Brandt, 1833: 203 (D♀).

**Type. *Holotype***: (♀), MYR_DIP_0000299.

**Type locality**. No locality label.

**Label data**. Holotype / *Julus (Spirostreptus)* / *Audouini* Nob. / Kunstkamer.

**Current nomenclature**. *Spirostreptus audouini* (Brandt, 1833) (Sierwald et al. 2026). However, the true generic assignment and identity of this species remain unknown (Golovatch and Hoffman 2000: 244).

**Remarks**. The holotype is dried, badly fragmented, kept in a carbon box (Fig. 1B), with no locality label. As evident from the enclosed label, this specimen came from the original collection of the Kunstkammer, which later became the ZISP (Slepkova 2008, 2017; Slepkova and Yusupova 2018).

***brevicornis* Brandt, 1840 (*Julus* )**

Fig. 11

**Figure 11.**
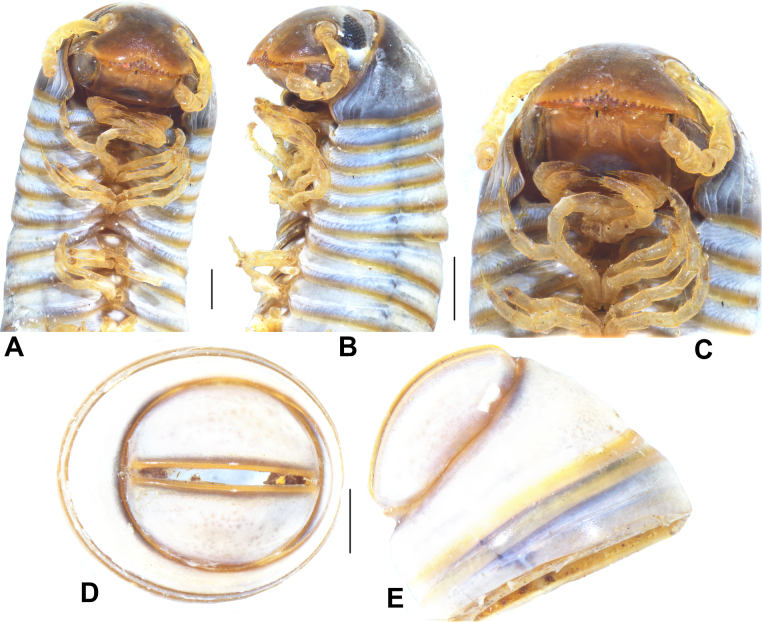
*“Spirostreptus” brevicornis* (Brandt, 1840) (= Julus (Spirostreptus) brevicornis Brandt, 1840), ♀ lectotype. **A–C**. Anterior part of body, ventral, lateral and ventral views, respectively; **D, E**. Posterior part of body, caudal and lateral views, respectively. Scale bar: 1 mm. Photographed by DVL.

*Julus brevicornis* Brandt, 1840c: 114 (D♂).

**Type. *Lectotype***: (♀), MYR_DIP_0000020.

**Type locality**. South Africa, Cape of Good Hope (no exact locality).

**Label data**. *Julus (Spirostreptus)* / *brevicornis* Brandt, / Опр. {Det.} Brandt / Сбор {Collected} Africa austr. / Caput Bonae Spei / leg. Brandt / 1♂ гололектотип / hololectotype // Lectotypus / design. S. Golovatch.

**Current nomenclature**. *Spirostreptus brevicornis* (Brandt, 1840) (Sierwald et al. 2026). Transferred to the genus *Spirostreptus* Brandt, 1833 by Attems (1914), but its identity is still questioned (Fig. 11), and the generic assignment to be put in quotation marks as it is clearly wrong. The lectotype of this species was designated by Hoffman and Golovatch (1998).

**Remarks**. In the original description, Brandt (1840c: 114) described the type series as “specimina dua, masculum adultum et juvenile”. Of these specimens, only the lectotype ♀ is currently available (also listed by Golovatch and Hoffman 2000: 236); the whereabouts of the other specimen remain unknown. The lectotype is broken, with its detached body parts being kept in a separate vial (Fig. 11).

***dubius* Brandt, 1840 (*Julus* )**

*Julus dubius* Brandt, 1840c: 126 (D♀).

**Type. *Syntypes***: (♀), MYR_DIP_0000287; (1♀), MYR_DIP_0000288; (1♀?, only central body segments available), MYR_DIP_0000289.

**Type locality**. Unknown country (*patria ignota*) and locality.

**Label data**. MYR_DIP_0000287: *Julus (Spirostreptus) du*- / *bius* Nob. / Ann. 42, Reg. par. 79 / Ann. Au 5 apt unico peli / qui excepto albimo it jec / nultino apouri..[illegible] vinir / par paribus / Dupont.

MYR_DIP_0000288: *Julus (Spirostreptus) dubius* Nob. / Annul. 42 / Patria ? Dupont.

MYR_DIP_0000289: *Julus (Spirostreptus) dubius* / Mar Nob. / Annul. 43 Reg. par. 75 / Annul. 7, 48, 42 et 43a. / podi. Sexcanter. 7 reg. par. / Patria ? Dupont.

**Current nomenclature**. *Julus dubius* Brandt, 1840 (Sierwald et al. 2026).

**Remarks**. All syntypes are dried and pinned, their handwritten labels are difficult/impossible to read; of the specimen MYR_DIP_0000289 only four central body rings are available.

***erythropareius* Brandt, 1840 (*Julus* )**

Fig. 12

**Figure 12.**
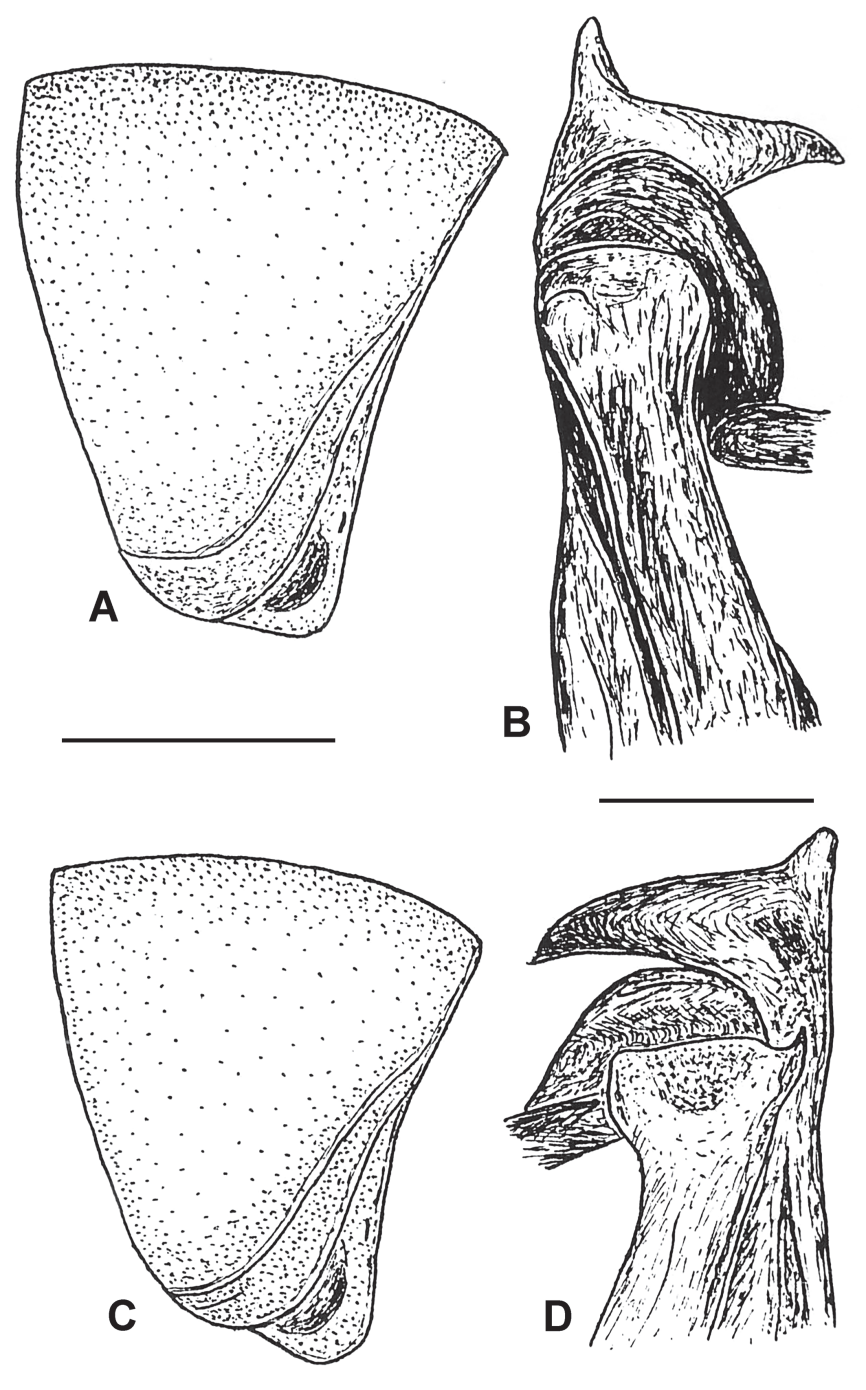
*Orthoporoides erythropareius* (Brandt, 1840) (= Julus (Spirostreptus) erythropareius Brandt, 1840), ♂ holotype (**A, B**) and *Orthoporoides trigonyger* (Brandt, 1840) (= Julus (Spirostreptus) trigonyger Brandt, 1840), ♂ holotype (**C, D**). **A, C**. Collum, lateral view; **B, D**. Tips of gonopods, posterior views. Scale bar: 1 mm. SIG del.

*Julus erythropareius* Brandt, 1840c: 111 (D♂).

**Type. *Holotype***: (♂), MYR_DIP_0000034.

**Type locality**. South Africa, Cape of Good Hope (no exact locality).

**Label data**. *Julus (Spirostreptus)* / *erythropareius* Brandt / Опр. {Det.} Brandt / Сбор {Collected} Caput Bonae Spei / Мыс Доброй Надежды {Cape of Good Hope} / 1♂ голотип holotype // 91 // Holotypus ♂ // *Gymnostreptus* / *erythropareius* (Brandt), 1841 / Опр. С. Головач {Det. S. Golovatch} / Сбор {Collected} Africa australis / 1♂ голотип holotype.

**Current nomenclature**. Accepted as *Spirostreptus erythropareius* (Brandt, 1840) by Sierwald et al. (2026). Hamer (1998: 57) referred to this combination as a Harpagophoridae species incertae sedis. In fact, this species belongs to the genus *Orthoporoides* Krabbe, 1982, thus forming a new combination: *Orthoporoides erythropareius* (Brandt, 1840), comb. nov. ex *Julus* Linnaeus, 1758 (det. SIG, see Fig. 12). Moreover, this is a new subjective senior synonym of *Orthoporoides trigonyger* (Brandt, 1840), syn. nov. (det. SIG).

**Remarks**. The species was described from the single male. The jar contains a vial with body parts and another with the gonopods.

***fischeri* Brandt, 1833 (*Julus* )**

Fig. 1C

*Julus fischeri* Brandt, 1833: 204, plate 5 (D♂).

**Type. *Holotype***: (♂), MYR_DIP_0000300.

**Type locality**. No exact locality.

**Label data**. Holotype / *Julus (Spirostreptus)* / *fischeri* Nob. / Kunstkamer.

**Current nomenclature**. *Spiropoeus fischeri* (Brandt, 1833) (Sierwald et al. 2026), the type species of the genus *Spiropoeus* Brandt, 1833. The true identity of this genus and species was revealed by Golovatch and Hoffman (2000) and Hoffman et al. (2001), even though Brandt (1833) had dissected and lost the gonopods.

**Remarks**. The holotype is dried, badly fragmented, kept in a carbon box (Fig. 1C), with no locality label. Its gonopods were extracted by Brandt but lost (Golovatch and Hoffman 2000: 245). As evident from the enclosed label, this specimen came from the original collection of the Kunstkammer, which later became the ZISP (Slepkova 2008, 2017; Slepkova and Yusupova 2018).

***flavofasciatus* Brandt, 1840 (*Julus* )**

Fig. 13

**Figure 13.**
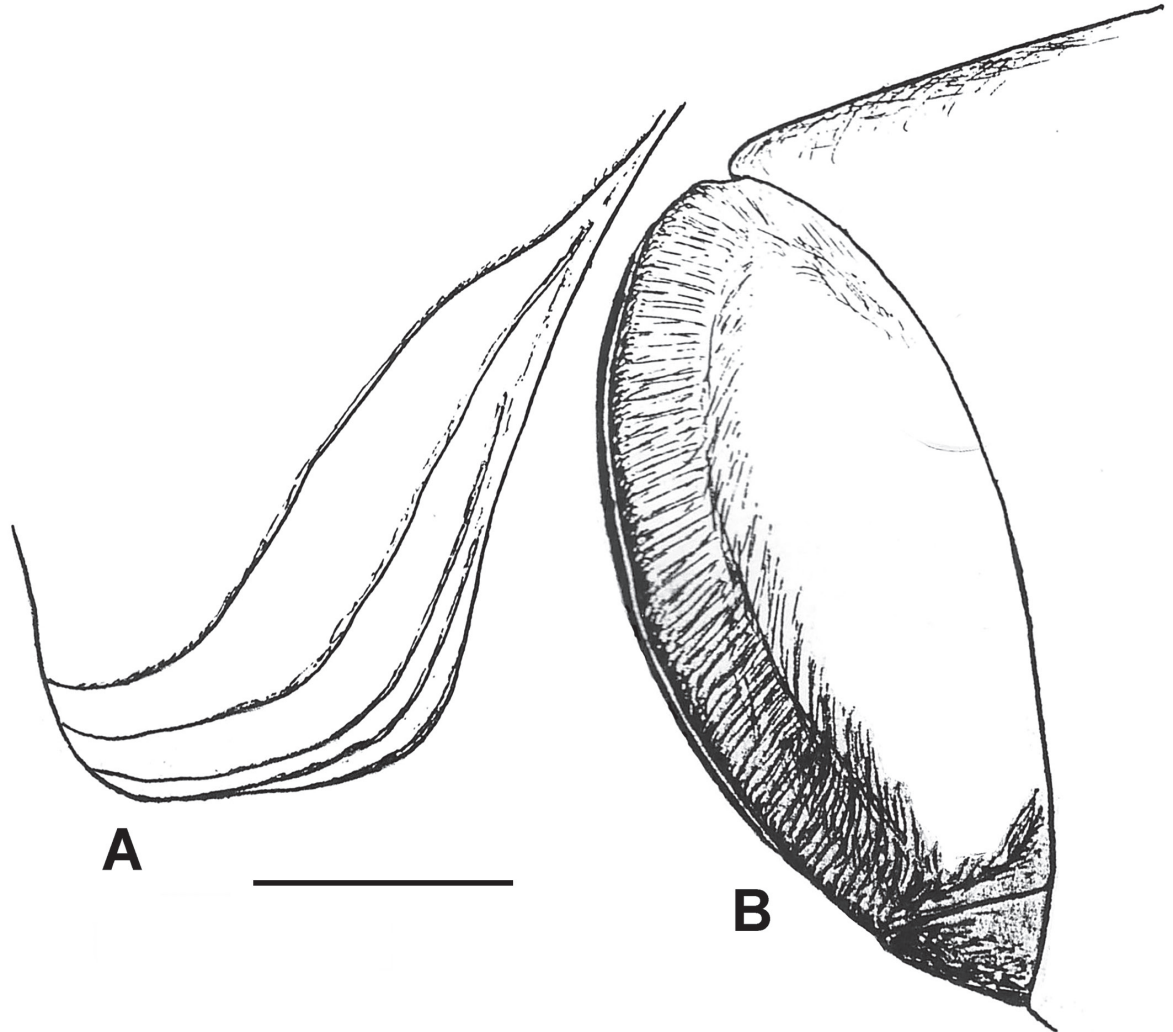
*“Spirostreptus” flavofasciatus* (Brandt, 1840) (= Julus (Spirostreptus) flavofasciatus Brandt, 1840), ♀ holotype. **A**. Ventral half of collum, lateral view; **B**. Telson, lateral view. Scale bar: 10 mm. SIG del.

*Julus flavofasciatus* Brandt, 1840c: 113 (D♀).

**Type. *Holotype***: (♀), MYR_DIP_0000293.

**Type locality**. South Africa, Cape of Good Hope (no exact locality).

**Label data**. Holotype ♀ / Зоол. Муз. АН {Zoological Museum of the Academy of Sciences} / *Julus (Spirostreptus)* / *flavofasciatus* Brandt, 1841 / Опр. {Det.} Brandt / Сбор {Collected} Africa merid., / Caput Bonae Spei / (Южн. Африка, Мыс Доброй Надежды) {South Africa, Cape of Good Hope} / leg. Brandt / 1♀ голотип {holotype} // *Jul. flavofasciatus* / fem. / Ann. an. [illegible] 32, alti- / mis. 2 [illegible] quinqu / ant. 4 qui paria Pei / par. 94. / Africa merid. Brandt // 90.

**Current nomenclature**. *Spirostreptus flavofasciatus* (Brandt, 1840) (Sierwald et al. 2026). Transferred to the genus *Spirostreptus* Brandt, 1833 and treated there as a nomen obscurum by Hamer (1998), but in fact it definitely belongs to a different genus (det. SIG, see Fig. 13).

**Remarks**. The holotype is dried and pinned. The second label of the holotype is rather illegible.

***laticollis* Brandt, 1840 (*Julus* )**

Fig. 14

**Figure 14.**
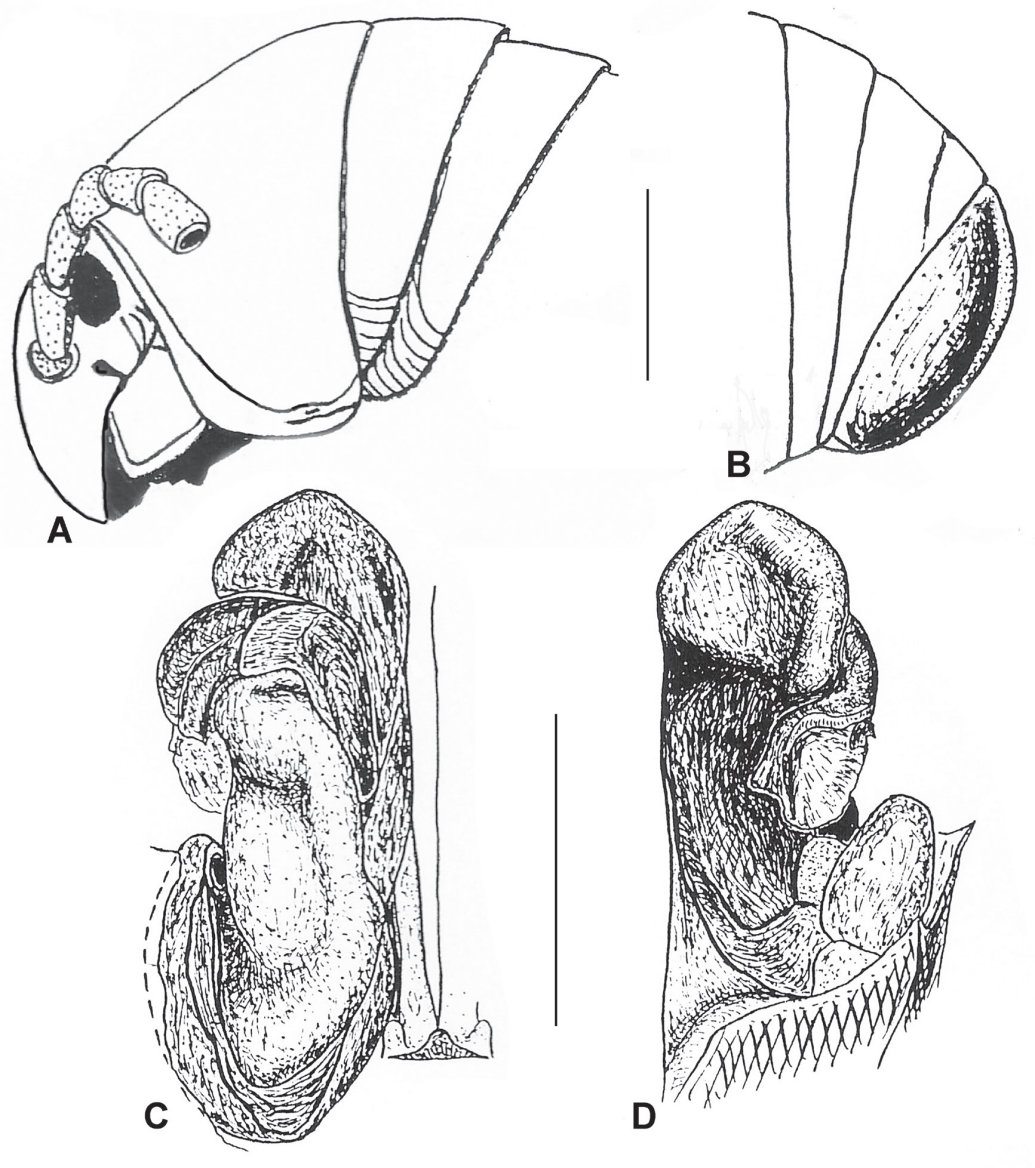
*Camaricoproctus laticollis* (Brandt, 1840), comb. nov. (= Julus (Spirostreptus) laticollis Brandt, 1840), ♂ holotype. **A**. Anterior part of body; **B**. Posterior part of body; **C, D**. Right gonopod, anterior and posterior views, respectively. Scale bars: 2.0 mm (**A, B**); 1.0 mm (**C, D**). SIG del.

*Julus laticollis* Brandt, 1840c: 110 (D♂).

**Type. *Holotype***: (♂), MYR_DIP_0000045.

**Type locality**. South Africa (no exact locality).

**Label data**. *Julus (Spirostreptus)* / *laticollis* Brandt / Опр. {Det.} Brandt / Сбор {Collected} / Africa australis / ♂ голотип {holotype} // *Camaricoproctus* / *laticollis* (Brandt) / Опр. С.И. Головач {Det. S.I. Golovatch} / Сбор {Collected} Южная Африка {South Africa} / leg. Brandt / голотип ♂ {holotype} // 88.

**Current nomenclature**. *Spirostreptus laticollis* (Brandt, 1840) (Sierwald et al. 2026). Transferred to the genus *Spirostreptus* Brandt, 1833 and also listed as incertae sedis by Hamer (1998), but in fact this species belongs to the genus *Camaricoproctus* Attems, 1926, thus forming a new combination: *Camaricoproctus laticollis* (Brandt, 1840), comb. nov. ex *Julus* Linnaeus, 1758 (det. SIG, see Fig. 14).

**Remarks**. The species was described from the holotype ♂ alone. The jar contains two vials: one with the broken holotype (moistened in alcohol), the other with its gonopods (labelled as ‘*Spirostreptus laticollis* Brdt gonopods’).

***melanopygus* Brandt, 1840 (*Julus* )**

Fig. 15

**Figure 15.**
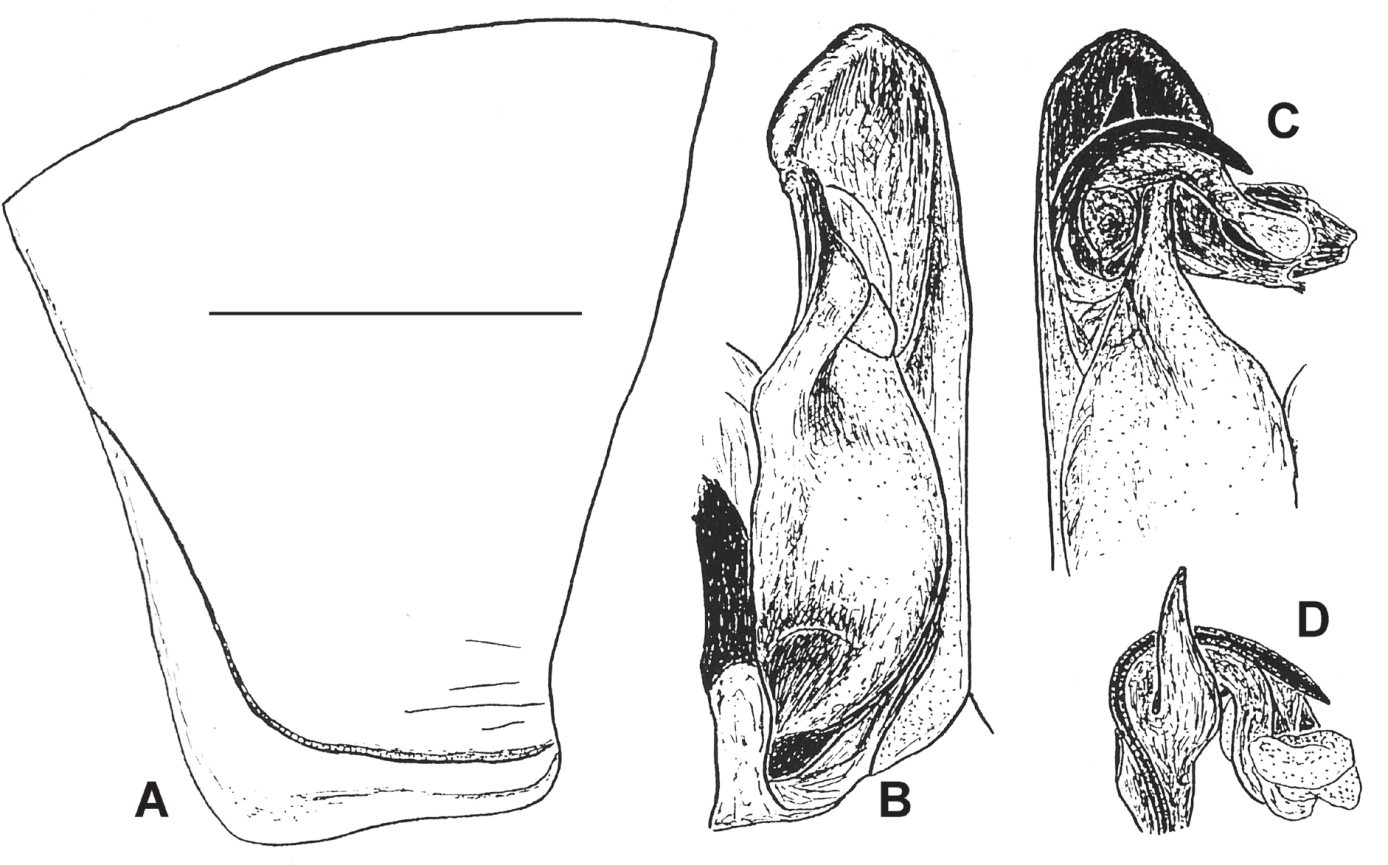
*Camaricoproctus melanopygus* (Brandt, 1840), comb. nov. (= Julus (Spirostreptus) melanopygus Brandt, 1840), ♂ holotype. **A**. Collum, lateral view; **B**. Left gonopod, anterior view; **C**. Distal part of left gonopod, posterior view; **D**. Terminal part of left gonopo telopodite, anterior view. Scale bar: 1.0 mm. SIG del.

*Julus melanopygus* Brandt, 1840c: 110 (D♂).

**Type. *Holotype***: (♂), MYR_DIP_0000044.

**Type locality**. South Africa, Cape of Good Hope (no exact locality).

**Label data**. Holotypus // *Camaricoproctus* / *melanopygus* (Brandt) / Опр. С.И. Головач {Det. S.I. Golovatch} / Сбор {Collected} Africa austr. / leg. Brandt / голотип ♂.

**Current nomenclature**. *Spirostreptus melanopygus* (Brandt, 1840) (Sierwald et al. 2026). Transferred to *Spirostreptus* Brandt, 1833 by Hamer (1998), but in fact this species belongs to the genus *Camaricoproctus* Attems, 1926, thus forming a new combination: *Camaricoproctus melanopygus* (Brandt, 1840), comb. nov. ex *Julus* Linnaeus, 1758 (det. SIG, see Fig. 15).

**Remarks**. The holotype is broken, with its gonopods being kept in a separate vial.

***sebae* Brandt, 1833 (*Julus* )**

Fig. 1D

*Julus sebae*Brandt 1833: 203, plate 5 (D♀).

**Type. *Holotype***: (♀), MYR_DIP_0000298.

**Type locality**. No exact locality, apparently from the Afrotropical Region (Hoffman et al. 2001).

**Label data**. Holotype / *Julus (Spirostreptus)* / *sebae* Nob. / Kunstkamer.

**Current nomenclature**. *Spirostreptus sebae* (Brandt, 1833), the type species of the genus *Spirostreptus* Brandt, 1833 (Sierwald et al. 2026). The identity of the genus and species was revealed by Hoffman et al. (2001), who demonstrated its Afrotropical origin.

**Remarks**. The holotype is dried, badly fragmented, kept in a carbon box (Fig. 1D), with no locality label, but the collector (Seba 1734) indicated it as ‘Oriental’; see Golovatch and Hoffman (2000) for further details. As evident from the enclosed label, this specimen came from the original collection of the Kunstkammer, which later became ZISP (Slepkova 2008, 2017; Slepkova and Yusupova 2018).

***sebae* var. *acutangula* Brandt, 1840 (*Julus* )**

Julus sebae var. acutangula Brandt, 1840c: 115 (D♀).

**Type. *Holotype***: (♀), MYR_DIP_0000290.

**Type locality**. Madagascar (no exact locality).

**Label data**. Holotype ♀ / Зоол. Муз. АН {Zoological Museum of the Academy of Sciences} / *Julus (Spirostreptus)* / *sebae* var. *acutangula* Brandt, 1841 / Опр. {Det.} Brandt / Сбор {Collected} Patria ignota / (родина неизвестна) / leg.? Dupont, Paris / 1♀ голотип {holotype} // *Julus (Spirostreptus) */ *sebae* / var. *acutan* / *gula* Brandt, 1841 / Patria ? Dupont.

**Current nomenclature**. *Spirostreptus acutangulus* (Brandt, 1840) (Sierwald et al. 2026), but the true generic assignment and identity of this species remain unknown (Golovatch and Hoffman 2000: 245).

**Remarks**. The holotype is dried and pinned; see Golovatch and Hoffman (2000) for its description.

***surinamensis* Brandt, 1840 (*Julus* )**

*Julus surinamensis* Brandt, 1840c: 118 (D♀).

**Type. *Syntype***: (1♀), MYR_DIP_0000296.

**Type locality**. Surinam (no exact locality).

**Label data**. *Julus (Spirostreptus)* / *surinamensis* Nob. / fem. gunios / Surinam Brandt.

**Current nomenclature**. *Spirostreptus surinamensis* (Brandt, 1841) (Sierwald et al. 2026). Transferred to the genus *Spirostreptus* Brandt, 1833 by Jeekel (1963), albeit with qualifications. Still referred to as such, although it may well be a species of *Gymnostreptus* Brölemann, 1902 (Jeekel 2009).

**Remarks**. This species was described from two females (Brandt 1840c: 119), so the ZISP specimen should be considered a syntype (Golovatch and Hoffman 2000); the whereabouts of the second female syntype is unknown. The syntype is dried and pinned, with the third part of its body missing.

***trigonyger* Brandt, 1840 (*Julus* )**

*Julus trigonyger* Brandt, 1840c: 119 (D♂).

**Type. *Holotype***: (♂), MYR_DIP_0000048.

**Type locality**. South Africa (no exact locality).

**Label data**. Зоол. Инст. АН {Zoological Institute of the Academy of Sciences} / *Julus (Spirostreptus)* / *trigonyger* / Опр. {Det.} Brandt / Сбор {Collected}. Afrique mer. / leg. Brandt / holotype / голотип // Holotypus ♂ // 92. // *Gymnostreptus* / *erythropareius* (Brandt) / Опр. С. Головач {Det. S. Golovatch} / Сбор {Collected} Южная Африка {South Africa} / leg. Brandt.

**Current nomenclature**. Accepted as *Spirostreptus trigonyger* (Brandt, 1840) by Sierwald et al. (2026). Transferred to *Spirostreptus* Brandt, 1833 by Hamer (1998), but this name is a subjective junior synonym of *Orthoporoides erythropareius* (Brandt, 1840), syn. nov. (det. SIG, Fig. 12; see also above).

**Remarks**. This species was described from the holotype ♂ only. The jar contains two vials: one with the broken holotype male consisting of several body fragments, another with its gonopods.

***validus* Brandt, 1840 (*Julus* )**

Fig. 16

**Figure 16.**
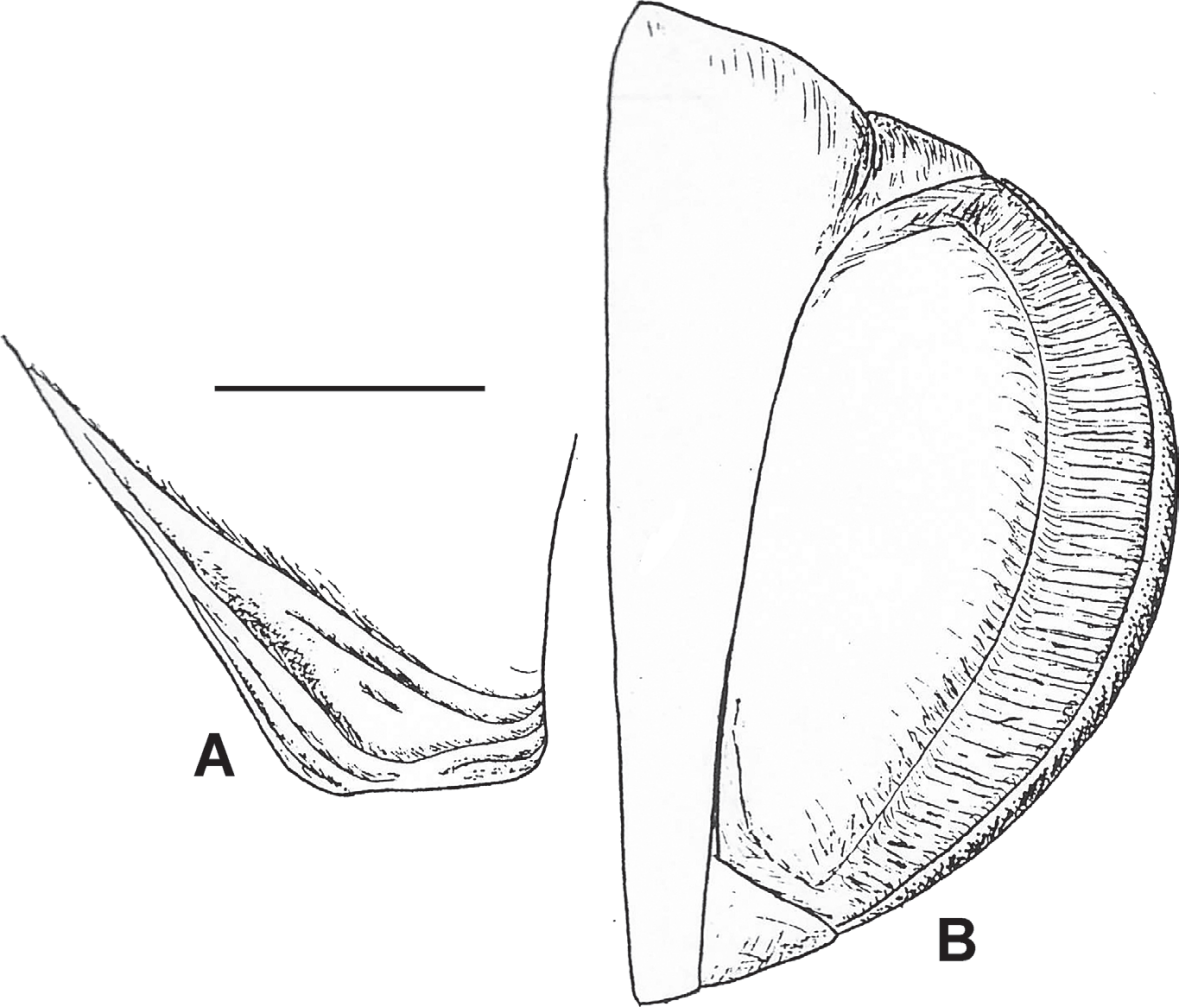
*“Spirostreptus” validus* (Brandt, 1840) (= Julus (Spirostreptus) validus Brandt, 1840), ♀ holotype. **A**. Ventral half of collum, lateral view; **B**. Telson, lateral view. Scale bar: 20 mm. SIG del.

*Julus validus* Brandt, 1840c: 116 (D♀).

**Type. *Holotype***: (♀), MYR_DIP_0000297.

**Type locality**. South Africa, Cape of Good Hope (no exact locality).

**Label data**. Голотип / Holotype ♀ / Зоол. Муз. АН {Zoological Museum of the Academy of Sciences} / *Julus (Spirostreptus) validus* Brandt, 1841 / Опр. {Det.} Brandt / Сбор {Collected} / Южная Африка, {South Africa} / Мыс Доброй Надежды {Cape of Good Hope} // *Julus (Spirostreptus)* / *validus* Nob. / Ann[illegible] 63 Red. p. 116 / fem. / Afrique Brandt.

**Current nomenclature**. *Spirostreptus validus* (Brandt, 1840) (Sierwald et al. 2026). Transferred to the genus *Spirostreptus* Brandt, 1833 by Hamer (1998) that is a nomen obscurum.

**Remarks**. The holotype is dried and pinned. The second label is partly illegible.

***walkenaerii* Brandt, 1841 (*Julus* )**

*Julus walkenaerii* Brandt, 1841c: 366 (D♂♀).

**Type. *Paralectotype***: (♀), MYR_DIP_0000033.

**Type locality**. Guiana (no exact locality).

**Label data**. *Julus (Spirostreptus)* / *walkenaerii* Brandt, 1841 / ♀ lectotype / design. S. Golovatch // *Julus (Spirostreptus)* / *walkenaerii* / Gwana // Lectotypus / design.

**Current nomenclature**. Accepted as *Spirostreptus walkenaerii* (Brandt, 1841) by Sierwald et al. (2026), even though Golovatch and Hoffman (2000: 237) had transferred this species name to the genus *Orthoporus* Silvestri, 1897.

**Remarks**. The lectotype ♂ is in ZMB. The ZISP paralectotype is broken, with its body parts being kept in a separate vial.

### Missing types

Below we present a list of Diplopoda species described mainly by H. Lohmander and J. Gulička based on specimens deposited in or borrowed from the ZISP collection (as evidenced by the content of their published papers). For various reasons, these types are absent from ZISP, and their current whereabouts remain unknown.

***caucasicus* Lohmander, 1932 (*Isobates* )**

*Isobates caucasicus* Lohmander, 1932b: 171, figs 1–3 (D♂♀).

**Remarks**. Based on the original description (Lohmander 1932a: 172), the type series consisted of 5♂ and 6♀ and collected from Georgia, Borjomi (ca 41°50'N, 43°23'E) on 7–9.VII.1929. Of these specimens five syntypes of unspecified sex (5 torsos MYRIA000014742, and 3 micropreparations MYRIA000014743) are kept in GMNH (O. Gran, pers. comm., 5.II.2026). The whereabouts of the remaining six syntypes remains unknown. However, the ZISP collection contains three samples with *I. caucasicus* collected from the so-called Terskaya Oblast, Nalchik Distr. (now the Republic of Kabardino-Balkaria) in 1917 and 1921, and identified by H. Lohmander.

***densecavum* Gulička, 1972 (*Altajosoma* )**

*Altajosoma densecavum* Gulička, 1972: 37, fig. 1.2 (D♂).

**Remarks**. Based on the original description (Gulička 1972: 38), the type series consisted of the holotype ♂ alone; it is currently absent from the ZISP collection.

***ferganus* Lohmander, 1933 (*Cylindroiulus* )**

*Cylindroiulus ferganus*Lohmander 1933: 67, figs 67, 68 (D♂♀).

**Remarks**. A series of three syntypes (1♂ 1♀ 1 juv. ♀) was studied by Lohmander (1933: 70), but none is currently available in the ZISP collection. The GMNH collection retains three micropreparations under this name (2♂ 1♀; MYRIA000014843 and MYRIA000014843, correspondingly), but it is unclear whether they indeed belong to the types (O. Gran, pers. comm., 5.II.2026). This species was re-examined by Read (1994), who did not mention any type specimens.

***furcatus* Lohmander, 1936 (*Brachydesmus* )**

*Brachydesmus furcatus* Lohmander, 1936: 16, figs 3–4 (D♂).

**Remarks**. A series of two syntypes (1♂ 1 juv.) was studied by Lohmander (1936: 18), but it is absent from the ZISP collection; none of the syntypes is available in GMNH (O. Gran, pers. comm., 5.II.2026).

***georgicus* Lohmander, 1932 (*Amblyiulus* )**

*Amblyiulus georgicus* Lohmander, 1932b: 180, figs 10–12 (D♂♀).

**Remarks**. A series of two syntypes (1♂ 1♀) from Borjomi (Georgia) was studied by Lohmander (1932b: 182), but it is absent from the ZISP collection; none of the syntypes is available in GMNH (O. Gran, pers. comm., 5.II.2026).

***kygae* Gulička, 1972 (*Ghilarovia* )**

*Ghilarovia kygae* Gulička, 1972: 39, fig. 2 (D♂♀).

**Remarks**. This species was described from a series composed of the holotype ♂ and several paratypes (5♀), of which the holotype was originally kept in the author’s collection (Gulička 1972: 38), whereas the paratypes were claimed to have been deposited in the Laboratory for Soil Zoology and General Entomology (former Lab of Soil Zoology) of IPEE. Unfortunately, only the holotype torso was returned after a scandal, whereas all paratypes have remained embezzled in the SMNPS collection. However, the current whereabouts of the holotype ♂ (MYR_DIP_0000060) remains unknown. No type specimens of this species are available in ZMMU (A. Schileyko, pers. comm., 4.II.2026).

***legitimus* Golovatch, 1980 (*Ansiulus* )**

*Ansiulus legitimus* Golovatch, 1980: 51, figs 3–8 (D♂♀).

**Remarks**. The holotype ♂ and all other paratypes (2♂ 4♀) are in HMNH. The paratypes (1♂ 1♀; MYR_DIP_0000146) that were supposed to be stored at ZISP are missing; no type specimens of this species are available in ZMMU (A. Schileyko, pers. comm., 4.II.2026).

***montanum* Gulička, 1972 (*Teleckophoron* )**

*Teleckophoron montanum* Gulička, 1972: 41, fig. 3.1–4 (D♂).

**Remarks**. Based on the original description (Gulička 1972: 42), the type series consisted of the holotype ♂ alone; it is currently absent from the ZISP collection and is kept embezzled in SMNPS.

***orientalis* Read, 1994 (*Cylindroiulus* )**

*Cylindroiulus orientalis* Read, 1994: 129, figs 16, 28–33 (D♂♀).

**Remarks**. Based on the original description (Read 1994: 129), a series of four paratypes (2♂ 2♀) should have been deposited in the ZISP collection, but none is currently available; apparently, all of them are now in ZMMU.

***pallida* Gulička, 1972 (*Altajella*)**

*Altajella pallida* Gulička, 1972: 42, figs 4.1–5 (D♂).

**Remarks**. This species was described from a series composed of the holotype ♂, which was originally kept in the author’s collection (Gulička 1972: 38). Only the holotype torso was returned to IPEE after a scandal, whereas the gonopods remain embezzled in the SMNPS collection. The current whereabouts of the holotype torso, which was supposed to be at ZISP (MYR_DIP_0000057), remain unknown. It is absent from ZMMU (A. Schileyko, pers. comm., 4.II.2026).

***phoenica orientalis* Lohmander, 1932 (*Brolemannia* )**

*Brolemannia phoenica orientalis* Lohmander, 1932a: 5, figs 2–4 (D♂).

**Remarks**. Only the holotype ♂ was studied by Lohmander (1932a: 6), but it is currently absent from the ZISP collection; it is not available in GMNH either (O. Gran, pers. comm., 5.II.2026).

***pinetorum* Gulička, 1972 (*Altajosoma* )**

*Altajosoma pinetorum* Gulička, 1972: 37, fig. 1.1 (D♂).

**Remarks**. Based on the original description (Gulička 1972: 38), the type series consisted of the holotype ♂ and three paratypes of unspecified sex; none of these specimens is currently available in the ZISP collection, apparently still being kept embezzled in SMNPS.

***sajanica* Gulička, 1972 (*Altajosoma* )**

*Altajosoma sajanica* Gulička, 1972: 37, fig. 1.3 (D♂).

**Remarks**. Based on the original description (Gulička 1972: 39), the type series consisted of the holotype ♂ only, which is currently absent from the ZISP collection, apparently still being kept embezzled in SMNPS.

***sokolowi* Lignau, 1924 (*Polyxenus* )**

*Polyxenus sokolowi* Lignau, 1924: 193, figs 7–13 (D♂♀).

**Remarks**. The type series likely consisted of several specimens of both sexes (Lignau 1924: 193), but it is currently absent from the ZISP collection. Short et al. (2020: 320) synonymised this species name with *Polyxenus argentifer* Verhoeff, 1921, and also noted that the original type series was probably lost during World War II.

***zarudnyi* Lohmander, 1932 (*Amblyiulus* )**

*Amblyiulus zarudnyi* Lohmander, 1932a: 23, figs 21–25 (D♂♀).

**Remarks**. Based on the original description (Lohmander 1932a: 23), the type series consisted of 3♂ and 8♀, of which only two female syntypes (3 torsos and 4 micropreparations; MYRIA000014630 and MYRIA000014630, respectively) are kept in GMNH (O. Gran, pers. comm., 5.II.2026). The whereabouts of the remaining nine syntypes are unknown, and none of them is in the ZISP collection.

### Nomina nuda

***kubanus paucisulcata* Lohmander, 1936 (*Unciger* )**

**Types. *Holotype***: (♀), MYR_DIP_0000264.

**Type locality**. Russia, Karachay-Cherkessia, Aksaut R. Valley (ca 43°54'N, 41°42'E).

**Label data**. Зоол. Муз. АН {Zoological Museum of the Academy of Sciences} / №270-1910 г. / 813. *Unciger kubanus* / *F. paucisulcatus* Lohm. / Опр. {Det.} Type. / Сбор {Collected} 4.IX.1910 г. / Куб. oб. {Kubanskaya Oblast} / Аксаутская караулка {Aksautskaya guardhouse} / под камнями {under stones}, А. Бируля {A. Birula} // *Unciger kubanus* / *F. paucisulcatus* / n.sp. / Type Hans Lohmander.

**Current nomenclature**. Unavailable as this taxon has never been described.

**Remarks**. This specimen has a standard label stating that it is a type for the subspecies *paucisulcata* Lohmander, 1936 of *U. kubanus* (see above). However, we failed to find its description or any reference in Lohmander (1936), nor does this name appear in Sierwald et al. (2026).

***maculicornis* Brandt (*Polydesmus* )**

**Types. *Syntypes***: (12♂ 10♀), MYR_DIP_0000261.

**Type locality**. Georgia, Tbilisi (ca 41°42'N, 44°48'E).

**Label data**. 421. *Polydesmus* / *maculicornis* n.sp. / Опр. {Det.} Brandt ? / Сбор {Collected} Тифлис {Tiflis} // *Polydesmus* / *maculicornis*, n.sp. / Tiflis Brdt. // 421 // *Brachydesmus assimilis* / Lohmander, 1936 / 12♂10♀ / det. S.I. Golovatch, 2015.

**Current nomenclature**. *Brachydesmus assimilis* Lohmander, 1936 (Sierwald et al. 2026). Golovatch et al. (2016) argued that Brandt had failed to describe “*Polydesmus maculicornis* Brandt” and hence this name was to be treated as a nomen nudum; these authors also identified Brandt’s series of specimens as *Brachydesmus assimilis* Lohmander, 1936 (see above).

**Remarks**. Some specimens are fragmented.

***pallens* Brandt (*Strongylosoma* )**

**Types. *Syntypes***: (12♀/juv.?), MYR_DIP_0000262.

**Type locality**. Georgia, Tbilisi (ca 41°42'N, 44°48'E).

**Label data**. 507. *Strongylosoma* / *pallens* n.sp. / Опр. {Det.} Brandt ? / Сбор {Collected} Тифлис. {Tiflis} // *Strongylosoma* / *pallens*, n.sp. / Tiflis Brdt. // 507.

**Current nomenclature**. Apparently, a nomen nudum; to date only two *Strongylosoma* species are known from the entire Caucasus (Evsyukov and Golovatch 2016).

**Remarks**. Some specimens are fragmented.

## Additional information

### Artificial Intelligence (AI) use

The authors accept full responsibility for the content of the manuscript, including the disclosure of any use of AI.

No AI tools were used in the preparation of this manuscript.

### Author ORCIDs

D.V. Logunov https://orcid.org/0000-0002-1983-7535

S.I. Golovatch https://orcid.org/0000-0001-7159-5484

### Data availability

All of the data that support the findings of this study are available in the main text.

## References

[B1] Anonymous (2026) [Nikolai Georgievich Lignau]. https://visacon.ru/stati/44606-lignau-nikolay-georgievich.html

[B2] Antić DŽ, Makarov SE (2016) The Caucasus as a major hotspot of biodiversity: Evidence from the millipede family Anthroleucosomatidae (Diplopoda, Chordeumatida). Zootaxa 4211(1): 1–205. 10.11646/zootaxa.4211.1.1 28006792

[B3] Antić DŽ, Reip HS (2020) The millipede genus *Leucogeorgia* Verhoeff, 1930 in the Caucasus, with descriptions of eleven new species, erection of a new monotypic genus and notes on the tribe Leucogeorgiini (Diplopoda: Julida: Julidae). European Journal of Taxonomy 713: 8–14. 10.5852/ejt.2020.713

[B4] Antić DŽ, Golovatch SI, Spelda J (2019) Identity of the millipede genus *Jaxartes* Verhoeff, 1930 (Diplopoda: Polydesmida: Polydesmidae), with descriptions of two new species from Central Asia. Arthropoda Selecta 28(4): 479–489.

[B5] Attems CMT Graf von (1914) Afrikanische Spirostreptiden nebst Überblick über die Spirostreptiden orbis terrarum. Zoologica 25(65–66): 1–235. https://www.biodiversitylibrary.org/page/10232973

[B6] Attems CMT Graf von (1937) Myriapoda 3. Polydesmoidea I. Fam. Strongylosomidae. Das Tierreich 68: 1–300. 10.1515/9783111567099

[B7] Bogachik TA, Dyakov VA, Ryasikov LV (2005) Life and activity of the Professor-Zoologist Nikolai Georgievich Lignau (to mark the 65^th^ anniversary of his death). I. I. Mechnikov Odessa National University. https://dspace.onu.edu.ua/server/api/core/bitstreams/3dd6a5da-f72b-4cb1-8d5d-7d51b339ac62/content

[B8] Brandt JF (1833) Tentaminum quorundam monographicorum InsectaMyriapoda Chilognathi Latreillii spectantium prodromus. Bulletin de la Societé Impériale des Naturalistes de Moscou 6: 194–209. http://biodiversitylibrary.org/page/45989137

[B9] Brandt JF (1840a) Remarques critiques sur les espèces qui composent le genre *Glomeris*, suivies de quelques observations sur leur distribution géographique. Bulletin Scientifique publié par l‘Académie Impériale des Sciences de Saint-Pétersbourg 7(4): 37–44.

[B10] Brandt JF (1840b) Remarques générales sur l‘ ordre des Insectes Myriapodes. Bulletin Scientifique publié par l‘Académie Impériale des Sciences de Saint-Pétersbourg 7(20): 293–328.

[B11] Brandt JF (1840c) Generis Juli specierum enumeratio, adjectis plurium, quae hucusque nondum innotuerunt specierum brevibus descriptionibus ad Musei Academiae Scientifiarum Petropolitanae specimina factis. Bulletin Scientifique publié par l‘Académie Impériale des Sciences de Saint-Pétersbourg 8(7): 97–128. http://biodiversitylibrary.org/page/44344298

[B12] Brandt JF (1841a) Recueil de Mémoires Relatifs à L‘ordre des Insectes Myriapodes, et lus à l‘Académie Impériale des Sciences de St.-Pétersbourg. Saint-Pétersbourg, 189 pp.

[B13] Brandt JF (1841b) Remarques critiques sur les espèces qui composent les genres *Spaherotherium* et *Sphaeropoeus*, suivies d‘une description de six espèces nouvelles des Sphaerothéries. Bulletin Scientifique publié par l‘Académie Impériale des Sciences de Saint-Pétersbourg 8(23): 357–365.

[B14] Brandt JF (1841c) Remarques supplémentaires au mémoire: Generis Juli specierum enumeratio, accompagnées de descriptions de trois espèces nouvelles. Bulletin Scientifique publié par l‘Académie Impériale des Sciences de Saint-Pétersbourg 8(23): 365–368.

[B15] Brandt JF (1841d) Note supplémentaire sur les espèces qui composent le genre *Polydesmus*, suivie d‘ une caractéristique de deux espèces nouvelles. Bulletin Scientifique publié par l‘Académie Impériale des Sciences de Saint-Pétersbourg 9(1): 9–11. https://biodiversitylibrary.org/page/44271052

[B16] Chamberlin RV (1921) The Julidae and Isobatidae in North America. Proceedings of the Biological Society of Washington 34: 81–84.

[B17] Enghoff H (1984) A revision of the Nopoiulinae, with notes on the classification of blaniulid millipedes (Diplopoda: Julida: Blaniulidae). Senckenbergiana Biologica 64(4–6): 393–428.

[B18] Enghoff H (1985) The milliped family Nemasomatidae. With description of a new genus, and a revision of *Orinisobates* (Diplopoda: Julida). Entomologica Scandinavica 15: 27–67. 10.1163/187631285x00045

[B19] Enghoff H (1987) Revision of *Nepalmatoiulus* Mauries, 1983 – a Southeast Asiatic genus of millipedes (Diplopoda: Julida: Julidae). Courier Forschungsinstitut Senckenberg 93: 241–331.

[B20] Enghoff H, Moravvej SA (2005) A review of the millipede fauna of Iran (Diplopoda). Zoology in the Middle East 35: 61–72. 10.1080/09397140.2005.10638104

[B21] Evsyukov AP, Golovatch SI (2016) The millipede genus *Strongylosoma* in the Caucasus (Diplopoda: Polydesmida, Paradoxosomatidae). Acta Societatis Zoologicae Bohemicae 80: 7–16. 10.11646/zootaxa.4085.1.1

[B22] Evsyukov AP, Golovatch SI, Reip HS (2018) The millipede genus *Julus* Linnaeus, 1758 in the Caucasus (Diplopoda: Julida: Julidae). Zootaxa 4461(1): 89–117. 10.11646/zootaxa.4461.1.7 30314099

[B23] Evsyukov AP, Golovatch SI, Reip H, VandenSpiegel D (2020) The millipede tribe Leptoiulini in the Caucasus, with notes on its generic classification (Diplopoda: Julida: Julidae). Zootaxa 4778(2): 237–280. 10.11646/zootaxa.4778.2.2 33055820

[B24] Evsyukov AP, Golovatch SI, Antić DŽ (2021) The millipede genera *Amblyiulus* Silvestri, 1896 and *Syrioiulus* Verhoeff, 1914 in the Caucasus, with notes on their distributions (Diplopoda, Julida, Julidae). ZooKeys 1048: 109–143. 10.3897/zookeys.1048.68454 PMC8292276 34316266

[B25] Gerstfeldt G (1858) Uber einige zum Theil neuen Arten Platoden, Anneliden, Myriapoden und Crustaceen Sibirien‘s. Namentlich seines östlichen Theiles und des Amur-Gebietes. Imperial Academy of Sciences: 1–36. https://www.biodiversitylibrary.org/page/37158036

[B26] Golovatch SI (1978) Some new East Asian millipedes (Diplopoda) in the collection of the Zoological Institute of the USSR Academy of Sciences. Entomologicheskoe Obozrenie 57(3): 677–681. [in Russian, English summary]

[B27] Golovatch SI (1979a) The composition and zoogeographic relationship of the diplopod fauna of Middle Asia. Part 1. Zoologicheskii Zhurnal 58(7): 987–1001. [in Russian, English summary]

[B28] Golovatch SI (1979b) A new genus for the USSR of the superfamily Parajuloidea (Diplopoda). Entomologicheskoe Obozrenie 58(4): 904–908. [in Russian, English summary]

[B29] Golovatch SI (1980) A contribution to the millipede fauna of Korea (Diplopoda). Folia Entomologica Hungarica 41(1): 49–58.

[B30] Golovatch SI (1981) Some new forms of millipedes (Diplopoda) from the Caucasus. Annales Zoologici 36(5): 105–116.

[B31] Golovatch SI (1983) On several new Glomeridae (Diplopoda) from Indochina. Annales historico-naturales Musei Nationalis Hungarici 75: 107–116.

[B32] Golovatch SI (1984a) Contributions to the millipede fauna of Vietnam (Diplopoda) I. Chordeumatida. Acta Zoologica Hungarica 29(1–3): 123–127.

[B33] Golovatch SI (1984b) Contributions to the millipede fauna of Vietnam (Diplopoda) II. Acta Zoologica Hungarica 30(1–2): 53–77.

[B34] Golovatch SI (1984c) Some new or less known Paradoxosomatidae (Diplopoda: Polydesmida) from India. Acta Zoologica Hungarica 30(3–4): 327–353.

[B35] Golovatch SI (1986a) Diplopoda from the Nepal Himalayas: Polydesmidae, Fuhrmannodesmidae. Senckenbergiana Biologica 66(4–6): 345–369.

[B36] Golovatch SI (1986b) The first Chordeumatida (Diplopoda) from Tasmania, with description of a new genus and three new species. Zoologische Jahrbücher, Abteilung für Systematik, Ökologie und Geographie der Tiere 113: 251–264.

[B37] Golovatch SI (1987) Diplopoda from the Nepal Himalayas, Opisotretidae, additional Polydesmidae and Furmannodesmidae. Courier Forschungsinstitut Senckenberg 93: 203–217.

[B38] Golovatch SI (1989) Diplopoda of the Caucasus, 2. Glomeridae, with contributions to the fauna of Turkey. Senckenbergiana Biologica 69(4–6): 421–440.

[B39] Golovatch SI (1991) The millipede family Polydesmidae in Southeast Asia with notes on phylogeny (Diplopoda: Polydesmida). Steenstrupia 17(4): 141–159.

[B40] Golovatch SI (2012) On several new or poorly-known Oriental Paradoxosomatidae (Diplopoda: Polydesmida), XII. Arthropoda Selecta 21(1): 1–12. 10.15298/arthsel.21.1.01

[B41] Golovatch SI (2013) On several new or poorly-known Oriental Paradoxosomatidae (Diplopoda: Polydesmida), XIV. Arthropoda Selecta 22(4): 307–332. 10.15298/arthsel.22.4.01

[B42] Golovatch SI (2014a) On several new or poorly-known Oriental Paradoxosomatidae (Diplopoda, Polydesmida), XV. Arthropoda Selecta 23(1): 1–19. 10.15298/arthsel.23.1.01

[B43] Golovatch SI (2014b) The myriapodological legacy of Victor Ivanovich Motschoulsky (1810–1871). ZooKeys 426: 11–16. 10.3897/zookeys.426.8011 PMC4137288 25147455

[B44] Golovatch SI (2015) Two new species of the millipede order Polydesmida from southern China (Diplopoda). Zoologicheskii Zhurnal 94(9): 1023–1028. [in Russian, with English summary] 10.15298/rusentj.24.1.01

[B45] Golovatch SI (2024) A new species and some new records of millipedes (Diplopoda) from southern Vietnam. Euroasian Entomological Journal 33(3): 407–413. 10.15298/rusentj.33.3.14

[B46] Golovatch SI, Hoffman RL (1989) Identity of *Polydesmus hamatus* Brandt 1841, a Malagasy milliped (DiplopodaPolydesmidaDalodesmidae). Tropical Zoology 2(2): 159–164. 10.1080/03946975.1989.10539436

[B47] Golovatch SI, Hoffman RL (2000) On the diplopod taxa and type material of J. F. Brandt, with some new descriptions and identities (Diplopoda). In: Wytwer G, Golovatch S (Eds) Progress in Studies on Myriapoda and Onychophora. Fragmenta Faunistica (Suppl.) 43: 229–249.

[B48] Golovatch SI, Korotayeva AM (2026) Review of the millipede genus *Platydesmus* Lucas, 1843 in Costa Rica, with the description of a new species (Diplopoda, Platydesmida, Platydesmidae). Arthropoda Selecta 35(2): 147–154. 10.15298/arthsel.35.2.01

[B49] Golovatch SI, Mikhaljova EV (1979) Neue Polydesmidea (Diplopoda) des Fernen Ostens. Zoologicheskii Zhurnal 58(6): 830–837. [in Russian, English summary]

[B50] Golovatch SI, Mikhaljova EV, Chang HW (2011) The millipede family Polydesmidae in Taiwan, with descriptions of five new species (Polydesmida, Diplopoda). ZooKeys 93: 9–42. 10.3897/zookeys.93.1167 PMC3095181 21594077

[B51] Golovatch SI, Evsyukov A, Reip H (2016) The millipede family Polydesmidae in the Caucasus (Diplopoda: Polydesmida). Zootaxa 4085(1): 1–51. 10.11646/zootaxa.4085.1.1 27394287

[B52] Golovatch SI, Izadi M, Habashi H, Shayanmehr M, Rahmani R, Rafiee F (2022a) An updated checklist of the millipedes of Iran (Diplopoda), with special emphasis on the fauna of Hyrcania, including noteworthy records of three species from its easternmost part. Arthropoda Selecta 31(3): 265–279. 10.15298/arthsel.31.3.01

[B53] Golovatch SI, Schileyko AA, Mikhailov KG (2022b) The myriapodological legacy of Vyacheslav Stepanovich Muralewicz (1881–1942?). Russian Entomological Journal 31(1): 92–97. 10.15298/rusentj.31.1.19

[B54] Golovatch SI, Korotayeva AM, Martínez-Torres D (2024) Three species of the millipede family Platyrhacidae from Costa Rica: A new *Barydesmus* Cook, 1896 and two oldest *Nyssodesmus* Cook, 1896 (Diplopoda, Polydesmida). Zootaxa 5538(5): 424–438. 10.11646/zootaxa.5538.5.2 39645693

[B55] Gulička J (1963) New millipedes (Diplopoda) from the USSR. Part 1. Zoologicheskii Zhurnal 42(4): 518–524. [in Russian, English summary]

[B56] Gulička J (1972) Some new species of Diplopoda in the USSR. Part II. Zoologicheskii Zhurnal 51(1): 36–45. [in Russian, English summary]

[B57] Hagström T (1992) Lohmander, H. Entomologisk Tidskrift 113(3): 31–35. [In Swedish]

[B58] Haase E (1880) Zur Kenntnis der sibirischen Myriapoden. Zoologischer Anzeiger 3(55): 223–225.

[B59] Hamer ML (1998) Checklist of Southern African millipedes (Myriapoda: Diplopoda). Annals of the Natal Museum 39: 11–82.

[B60] Hoffman RL (1999) Checklist of the millipedes of North and Middle America. Virginia Museum of Natural History, special publication 8: 1–584. https://www.biodiversitylibrary.org/page/63590410

[B61] Hoffman RL, Golovatch SI (1998) Studies on spirostreptid millipeds. XXV. On the status of some harpagophorid millipeds named by C. Attems, in the Vienna Natural History Museum (Spirostreptida: Harpagophoridae). Annalen des Naturhistorischen Museums in Wien (Serie B) 100: 481–488.

[B62] Hoffman RL, Golovatch SI, Hamer M (2001) Identities of the milliped genera *Spirostreptus* Brandt, 1833 and *Spiropoeus* Brandt, 1833 (Diplopoda, Spirostreptida, Spirostreptidae). Myriapodologica 7(5): 35–47.

[B63] ICZN (1999) International Code of Zoological Nomenclature. The Natural History Museum, London, 306 pp. 10.5962/bhl.title.50608

[B64] Jeekel CAW (1963) Diplopoda of Guiana (1–5). Studies on the fauna of Suriname and other Guyanas 4: 1–157.

[B65] Jeekel CAW (1988) The generic position of *Orthomorpha bucharensis* Lohmander and *O. muminabadensis* Gulička, and the taxonomic status of *Hedinomorpha* Verhoeff (Diplopoda, Polydesmida, Paradoxosomatidae). Bulletin Zoölogisch Museum Universiteit van Amsterdam 11(11): 97–104.

[B66] Jeekel CAW (2001) A bibliographic catalogue of the Asiatic Sphaerotheriida (Diplopoda). Myriapod Memoranda 3: 5–38.

[B67] Jeekel CAW (2009) Checklist of the Diplopoda reported from Suriname. Myriapod Memoranda 11: 19–30.

[B68] Korsós Z (2001) Diplopoda from the Nepal Himalaya: towards the clarification of the genus *Anaulaciulus* Pocock 1895 (Diplopoda, Julida, Julidae, Brachyiulini). Senckenbergiana Biologica 81(1–2): 61–86.

[B69] Krabbe E (1982) Systematik der Spirostreptidae (Diplopoda: Spirostreptomorpha). Abhandlungen des Naturwissenschaftlichen Vereins in Hamburg, N.F. 21: 1–476.

[B70] Kraus O (1960) Äthiopische Diplopoden I. Monographie der Odontopygidae-Odontopyginae (Diplopoda, Spirostreptoidea). Annalen van het Koninklijk Museum van Belgisch-Congo (reeks in 8°) 82: 1–207.

[B71] Lang J (1959) To the knowledge of millipedes (Diplopoda) of the USSR territory. Zoolologicheskiy Zhurnal 38(12): 1790–1796. [in Russian, English summary]

[B72] Lignau N (1903) Myriapods of the Black Sea coast of the Caucasus. Die Myriopoden am Kaukasischen Schwarzmeerufer. Mémoires de la Société Impériale des Naturalistes de la Nouvelle-Russie [Zapiski Imperatorskogo Novorossiyskogo obshchestva estestvoispytateley]. Odessa 25(1): 82–149. [in Russian and German]

[B73] Lignau NG (1907) K faune mnogonozhek Kavkaza. To the myriapoda fauna of the Caucasus. Ezhegodnik Zoologicheskogo Muzeya Imperatorskoi Akademii Nauk 12: 195–200. [In Russian]

[B74] Lignau NG (1915 [for 1914]) Vielfüßer aus Abchasien. Annuaire du Musée Zoologique de l‘Académie Imériale des Sciences de St.-Pétersbourg 18: 349–401.

[B75] Lignau NG (1924) [Novye predstaviteli roda *Polyxenus*]. Les nouveaux représentants du genre *Polyxenus*. Journal of Research Chairs in Odessa 1(10–11): 190–199. [In Russian]

[B76] Lignau NG (1929) Zur Kenntnis der zentralasiatischen Myriopoden. Zoologischer Anzeiger 85(5–8): 159–175.

[B77] Lignau NG (1933) Einiges über die Myriopodenfauna der Ukraine. Entomologicheskoe Obozrenie 25(1–2): 134–144.

[B78] Lindroth CH (1961) Lohmander, H. Opuscula Entomologica, Lund 26: 1–3. [In Swedish]

[B79] Lohmander H (1931) *Caspiopetalum*, eine neue Diplopoden-Gattung aus dem russischen Zentralasien. Arkiv för zoologi 22B(1): 1–6.

[B80] Lohmander H (1932a) Neue Diplopoden aus Persien. Göteborgs Kungliga Vetenskaps- och Vitterhets-Samhälles handlingar, 5. Följden, Ser. B 3(2): 1–44.

[B81] Lohmander H (1932b) Neue transkaukasische Diplopoden. 4. Aufsatz über Diplopoden aus der Sowjet-Union. Zoologischer Anzeiger 98(7–8): 171–182.

[B82] Lohmander H (1933) Über Diplopoden aus Zentralasien. Arkiv för Zoologi 25A(6): 1–71.

[B83] Lohmander H (1936) Über die Diplopoden des Kaukasusgebietes. Göteborgs Kungliga Vetenskaps- och Vitterhets-Samhälles handlingar, 5. Följden, Ser. B 5(1): 1–196.

[B84] Lokšina IE, Golovatch SI (1979) Diplopoda of the USSR fauna. Pedobiologia 19(6): 381–389. 10.1016/s0031-4056(23)02243-6

[B85] Martynov AV, Petrenko NA, Korzhova TO, Balashov IA (2021) Rediscovery of the Lohmander’s collection of Diplopoda from Ukraine. Ecologica Montenegrina 44: 19–25. 10.37828/em.2021.44.3

[B86] Mauriès JP (1984) Deux espèces nouvelles de Diplopodes cavernicoles des Cyclades: *Hyleoglomeris beroni* (Glomerida) et *Syrioiulus andreevi* (Iulida). Biologia Gallo-Hellenica 11: 37–49.

[B87] Mikhaljova EV (1982) On the synonymy of two Diplopoda forms from the Soviet Far East. Zoologicheskii Zhurnal 61: 1265–1268. [in Russian, English summary]

[B88] Mikhaljova EV (2002) On some poorly-known millipedes from Siberia (Diplopoda). Arthropoda Selecta 10(3): 201–207. [for 2001] 10.3161/00159301ff2000.43.10.109

[B89] Mikhaljova EV (2017) The millipede fauna (Diplopoda) of the Asian part of Russia. Vladivostok, Dalnauka Publishers, 336 pp. [in Russian, English summary]

[B90] Mikhaljova EV (2019) Identities of the millipede genera *Skleroprotopus* Attems, 1901 and *Ansiulus* Takakuwa, 1940 (Diplopoda: Julida: Mongoliulidae), with emphasis on the postembryonic development of *Skleroprotopus coreanus* (Pocock, 1895). Zootaxa 4551(5): 501–529. 10.11646/zootaxa.4551.5.1 30790791

[B91] Mikhaljova EV, Golovatch SI (1981) Polymorphism in a new species of the genus *Epanerchodus* (Diplopoda, Polydesmidae) from the Soviet Far East. Zoologicheskii Zhurnal 60(8): 1183–1189. [in Russian, English summary]

[B92] Mikhaljova EV, Marusik YuM (2004) New data on taxonomy and fauna of the millipedes (Diplopoda) from the Russian Far East, Siberia and Mongolia. Far Eastern Entomologist 133: 1–12.

[B93] Mikhaljova EV, Nefediev PS, Nefedieva J, Sakhnevich MB, Dyachkov YuV (2014) Review of the millipede genus *Sibiriulus* Gulička, 1972, with descriptions of three new species from Altai, Siberia, Russia (Diplopoda, Julida, Julidae). Zootaxa 3866(1): 30–52. 10.11646/zootaxa.3866.1.2 25283646

[B94] Moritz M, Fischer SC (1978) Die Typen der Myriapoden-Sammlung des Zoologischen Museums Berlin. I. Diplopoda. Teil 4: Polydesmida. Teil 5: Ergänzungen. Mitteilungen aus dem Zoologischen Museum in Berlin 54(1): 99–160. 10.1002/mmnz.19780540106

[B95] Nefediev PS (2018) *Julus ghilarovi* Gulička, 1963 *s.str*.: Time to put an end to confusion (Diplopoda: Julida: Julidae). Arthropoda Selecta 27(4): 284–292. 10.15298/arthsel.27.4.03

[B96] Nguyen DA, Sierwald P (2013) A worldwide catalog of the family Paradoxosomatidae Daday, 1889 (Diplopoda: Polydesmida). Check List 9(6): 1132–1353. 10.15560/9.6.1132

[B97] Olenev NO, Popov VV (1945) In memoriam of Prof. VV Redikorzev (1.IX.187–28.I.1942). Priroda 2: 90–91. [In Russian]

[B98] Porat CO von (1872) Myriopoder från Vestoch Syd-Afrika. Bihang till Kongliga Svenska vetenskaps-akademiens handlingar 18, 4(7): 1–52.

[B99] Read HJ (1992) The genus *Cylindroiulus* Verhoeff 1894 in the faunas of the Caucasus, Turkey and Iran (Myriapoda: Diplopoda: Julidae). Senckenbergiana Biologica 72(4–6): 373–433.

[B100] Read HJ (1994) The milliped genus *Cylindroiulus* Verhoeff, 1894, in Middle Asia (DiplopodaJulidaJulidae). Arthropoda Selecta 3(1–2): 117–132.

[B101] Redman GT, Hamer ML, Barraclough DA (2003) Revision of the Harpagophoridae (Diplopoda, Spirostreptida) of southern Africa, including descriptions of five new species. African Invertebrates 44(2): 203–277. 10.5281/zenodo.7666374

[B102] Seba A (1734) Locupletissima rerum Naturalium Thesauri Accurata Descriptio et Iconibus Artificiosissimis Expressio per Universam Physices Historiam (Tomus 1). Amstelaidami, 178 pp. 10.5962/bhl.title.62760

[B103] Shinohara K (1977) Revaluation on *Riukiaria* (Diplopoda). Acta Arachnologica 27 (special number): 115–119. 10.2476/asjaa.27.specialnumber_115

[B104] Short M, Vahtera V, Wesener T, Golovatch SI (2020) The millipede family Polyxenidae (Diplopoda, Polyxenida) in the faunas of the Crimean Peninsula and Caucasus, with notes on other European Polyxenidae. Zootaxa 4772(2): 306–332. 10.11646/zootaxa.4772.2.4 33055614

[B105] Sierwald P, Decker P, Spelda J (2026) MilliBase. https://www.millibase.org [on 2026-01-4]

[B106] Slepkova N, Yusupova T (2018) The Zoological Museum of the St. Petersburg Academy of Sciences, 1860s – 1900s: from an academic institute to a public museum. Centaurus 60(4): 294–314. 10.1111/1600-0498.12193

[B107] Slepkova NV (2008) Reorganization of the Academy of Sciences in 1929–1931 and the Zoological Museum. In: Afiani VYu, Sorokina MV, Tunkina IA (Eds) Akademicheskij Arhiv v Proshlom i Nastojashhem. Saint-Petersburg: Izd-vo Nestor-Istorija, 292–302. [In Russian]

[B108] Slepkova NV (2013) Catalogues of the Zoological Museum in the personal fund of Academician FF Brandt. In: Tunkina IV (Ed.) Miller’s Readings: To the 285^th^ Anniversary of the Archive of the Russian Academy of Sciences. Collection of Scientific Articles on the Materials of the International Scientific Conference 23 April 25, 2013, St. Petersburg. Nestor-Istoriia, St. Petersburg, 191–198. [In Russian]

[B109] Slepkova NV (2016) Zoological Museum of the Imperial Academy of Sciences in St. Petersburg in the 19^th^ century: Principles of exposure. Studies in the History of Biology 8(1): 29–65. [in Russian, English summary]

[B110] Slepkova NV (2017) The Zoological Museum in St. Petersburg and the development of taxonomy: 300 years of change. Museum – Monument – Inheritance 1: 7–17. [in Russian, English summary]

[B111] Sokolov VE, Shishkin BC (2005) The Development of domestic theriology in the nineteenth century. In: Pavlov DS (Ed.) Progress in Russian theriology in 19^th^ century. Nauka, Moscow, 111–158. [In Russian]

[B112] Stoev P, Enghoff H (2005) A new cave-dwelling millipede of the genus *Bollmania* Silvestri, 1896 from Yunnan, China, with remarks on the reduction of the second female leg-pair (Diplopoda: Callipodida: Caspiopetalidae). Journal of Natural History 39(21): 1875–1891. 10.1080/00222930400025896

[B113] Strasser K (1971) Catalogus Faunae Jugoslaviae. Diplopoda. Consilium Academiarum Scientiarum Rei Publicae Socialisticae Foederativae Jugoslaviae. Academia Scientiarum et Artium Slovenica, Ljubljana, 48 pp.

[B114] Strauch AA (1889) Zoological Museum of the Imperial Academy of Sciences: The fiftieth anniversary of its existence. Tipografiia Imperatorskoi Akademii nauk, St. Petersburg, 372 pp. [In Russian]

[B115] Stuxberg A (1876a) Myriopoder fran Sibirien och Waigatsch ön samlade under Nordenskiöldska expeditionen 1875. Öfversigt af Kongliga Vetenskaps-Akademiens förhandlingar 1876(2): 11–38.

[B116] Stuxberg A (1876b) On the Myriapoda, from Siberia and Waigatsch Island, collected during the Expedition of Prof. Nordenskiöld, 1875. Annals and Magazine of Natural History, ser. 4. 17(100): 306–318. 10.1080/00222937608681955

[B117] Timotheew TE (1897) Two new species of Diplopoda. Trudy Obshchestva ispytateley prirody pri Kharkovskom universitete 31: 1–20. [In Russian]

[B118] Vagalinski B (2020) A new species of *Syrioiulus* Verhoeff, 1914 from Iran, with remarks on the taxonomy of the genus (Diplopoda: Julida: Julidae). Revue Suisse de Zoologie 127(1): 83–94. 10.35929/RSZ.0008

[B119] Vagalinski B, Golovatch SI (2021) The millipede tribe Brachyiulini in the Caucasus (Diplopoda, Julida, Julidae). ZooKeys 1058: 1–127. 10.3897/zookeys.1058.68628 PMC8421324 34557053

[B120] Vagalinski B, Lazányi E (2018) Revision of the millipede tribe Brachyiulini Verhoeff, 1909 (Diplopoda: Julida: Julidae), with descriptions of new taxa. Zootaxa 4421(1): 1–142. 10.11646/zootaxa.4421.1.1 30313518

[B121] VandenSpiegel D, Golovatch SI (2023) On two poorly-known species of the millipede family Polydesmidae (Diplopoda: Polydesmida) from Central Asia. Arthropoda Selecta 32(1): 15–22. 10.15298/arthsel.32.1.02

[B122] VandenSpiegel D, Golovatch SI, Hamer ML (2002) Revision of some of the oldest species in the millipede genus *Sphaerotherium* Brandt, 1833 (Diplopoda, Sphaerotheriida, Sphaerotheriidae), with new synonymies. In: 12^th^ International Congress of Myriapodology, Mtunzini, South Africa, July 2002. University of Natal, Pietermaritzburg, Republic of South Africa, 39 pp.

[B123] Wesener T (2016) The Giant Pill-Millipedes, order Sphaerotheriida – An annotated species catalogue with morphological atlas and list of apomorphies. Bonn Zoological Bulletin Supplementum 63: 1–107. 10.4126/frl01-006408913

[B124] Wesener T, Akkari N, Golovatch SI (2025) Revision of the millipede family Dalodesmidae in Madagascar, with descriptions of two new Malagasy species of *Dalodesmus* Cook, 1896 (Diplopoda, Polydesmida). ZooKeys 1223: 185–220. 10.3897/zookeys.1223.139346 PMC11736306 39822655

